# Structure Prediction and Potential Inhibitors Docking of Enterovirus 2C Proteins

**DOI:** 10.3389/fmicb.2022.856574

**Published:** 2022-04-29

**Authors:** Daoqun Li, Leiliang Zhang

**Affiliations:** ^1^Department of Clinical Laboratory Medicine, The First Affiliated Hospital of Shandong First Medical University and Shandong Provincial Qianfoshan Hospital, Jinan, China; ^2^Department of Pathogen Biology, School of Basic Medical Sciences, Shandong First Medical University and Shandong Academy of Medical Sciences, Jinan, China; ^3^Medical Science and Technology Innovation Center, Shandong First Medical University and Shandong Academy of Medical Sciences, Jinan, China

**Keywords:** enteroviruses, non-structural 2C protein, structure modeling, antiviral inhibitors, molecular docking

## Abstract

Human enterovirus infections are mostly asymptomatic and occasionally could be severe and life-threatening. The conserved non-structural 2C from enteroviruses protein is a promising target in antiviral therapies against human enteroviruses. Understanding of 2C-drug interactions is crucial for developing the potential antiviral agents. While functions of enterovirus 2C proteins have been widely studied, three-dimensional structure information of 2C is limited. In this study, the structures of 2C proteins from 20 enteroviruses were simulated and reconstructed using *I-TASSER* programs. Subsequent docking studies of the known 22 antiviral inhibitors for 2C proteins were performed to uncover the inhibitor-binding characteristics of 2C. Among the potential inhibitors, the compound hydantoin exhibited the highest broad-spectrum antiviral activities with binding to 2C protein. The anti-enteroviral activity of GuaHCL, compound 19b, R523062, compound 12a, compound 12b, quinoline analogs 12a, compound 19d, N^6^-benzyladenosine, dibucaine derivatives 6i, TBZE-029, fluoxetine analogs 2b, dibucaine, 2-(α-hydroxybenzyl)-benzimidazole (HBB), metrifudil, pirlindole, MRL-1237, quinoline analogs 10a, zuclopenthixol, fluoxetine, fluoxetine HCl, and quinoline analogs 12c showed a trend of gradual decrease. In addition, the free energy with 22 compounds binding to EV 2C ranged from −0.35 to −88.18 kcal/mol. Our *in silico* studies will provide important information for the development of pan-enterovirus antiviral agents based on 2C.

## Introduction

Human enteroviruses (EVs) are genetically classified to seven viral species, including enterovirus A–D and rhinovirus A–C in the genus enterovirus from the family *Picornaviridae* (Baggen et al., 2018). They cause a wide range of diseases ranging from enteric or respiratory infections, hand-foot-and-mouth disease (HFMD), conjunctivitis to acute flaccid paralysis, viral myocarditis, fulminant pancreatitis, or aseptic meningitis. Infections are often self-limiting but could result in severe complications that are fatal in some cases, especially in the Asia-Pacific region (Gao et al., 2021). Many strategies have been applied to control EV infections. One strategy involves the development of vaccines. Inactivated and life-attenuated vaccines have been developed against poliovirus (PV). Recently, inactivated vaccines against enterovirus A71 (EV-A71) were approved in China (Guan et al., 2020). However, the inactivated EV-A71 vaccine had a weak cross-genotype and long-term protection. Another strategy is the development of potential antivirals. These include both direct-acting antivirals, most of which bind to the viral capsid or the viral protease 3C, and inhibitors that target host factors essential for virus replication. These inhibitors were tested in clinical trials, but their development was halted due to limited efficacy, poor bioavailability, or toxicity issues. To date, no FDA-approved antiviral agent against EVs is licensed for therapeutic use (Zhang et al., 2020). Understanding of viral proteins–drugs interactions is important for virus resistance and potential antiviral targets.

An attractive target for enteroviruses antivirals is the highly conserved and multifunctional non-structural 2C protein. 2C protein is an ATPase associated with diverse cellular activities (AAA+ ATPase) classified within the superfamily 3 (SF3) helicases (Guan et al., 2017). These enzymes couple the hydrolysis of ATP to movement of protein domains which, in turn, drive the unwinding of a nucleic acid substrate. 2C functions as RNA helicase and ATP-independent chaperoning activities for viral RNA replication. 2C fulfills pleiotropic functions during the virus life cycle, including replication organelle formation, genome replication, and encapsidation (Guan et al., 2018). However, the crystal structure information on enteroviruses 2C proteins is limited. Moreover, the spectrum of antiviral activity against enteroviruses 2C, as well as their target domains, remains inconclusive.

In this study, we simulated the three-dimensional (3D) structures of 20 EV 2C proteins (EV-A71, EV-D68, EV-D70, PV-1, PV-2, PV-3, CV-A6, CV-A9, CV-A10, CV-A16, CV-A21, CV-A24, CV-B3, HRV-A, HRV-A2, HRV-B, HRV-B14, HRV-C, Echovirus E11, and Echovirus E30). These 2C proteins were docked onto 22 known and newly reported 2C inhibitory compounds (Vance et al., 1997; Ulferts et al., 2013; Bauer et al., 2020; Wang et al., 2020). These compounds showed potential broad-spectrum anti-EV activity and inhibited contemporary strains of emerging EVs of public health concern, which was consistent with reported data. In addition, we calculated and predicted the target-binding sites of new drug compounds interacting with 2C, which provided a perspective for the understanding of drug design and mechanism of action for EV 2C protein. Importantly, these results indicate that the predicted model of 2C complex displays high quality and lacks any serious steric clashes. Therefore, this model could pave the way for further structural, functional, and therapeutic studies, specifically concerned with *in silico* studies. Additionally, our study can help in future mutational studies to pinpoint the roles of various amino acid residues and to predict the effects of mutations on 2C structure, and its biological functions.

## Materials and Methods

### Sequence Alignment and Homology Modeling of Enteroviruses 2C

Alignment of the deduced amino acid sequence of enteroviruses 2C proteins is downloaded from EV-A71 (GenBank no. ADV76475.1), EV-D70 (GenBank no. QJA10589.1), EV-D68 (GenBank no. AJI77528.1), PV-1 (GenBank no. AAG27168.2), PV-2 (GenBank no. AUF49673.1), PV-3 (GenBank no. AUF49621.1), CV-A6 (GenBank no. QBM01047.1), CV-A9 (GenBank no. AFN25851.1), CV-A10 (GenBank no. QJA28496.1), CV-A16 (GenBank no. AWU78870.1), CV-A21 (GenBank no. AXF50737.1), CV-A24 (GenBank no. ABM54549.1), CV-B3 (GenBank no. AFD33642.1), HRV-A (GenBank no. AFM84630.1), HRV-A2 (GenBank no. QGA30984.1), HRV-B (GenBank no. AFM84628.1), HRV-B14 (GenBank no. NP_041009.1), HRV-C (GenBank no. AET25077.2), Echovirus E11 (GenBank no. CAE12182.1), and Echovirus E30 (GenBank no. AUF49665.1). Multiple sequence alignment is analyzed by MEGA-X. The conserved domains were annotated by National Center for Biotechnology Information (NCBI) Conserved Domain Database (CDD) as described earlier. The full-length crystal structure of EVs 2C protein is not available in the Protein Data Bank (PDB) (*https://www.rcsb.org/)*. Hence, based on the resolved enterovirus EV-A71 2C protein crystal structure (PDB ID:5GQ1 and 5GQB, resolved at 2.49Å), we expanded the structural coverage of these 2C proteins and modeled these 3D structures of the full-length sequence protein model of enterovirus 2C protein using *I-TASSER* programs (as “Zhang-Server,” *https://zhanglab.ccmb.med.umich.edu/I-TASSER/*). Models built using I-TASSER were assessed with normalized DOPE scores along with their C-scores, predicted TM scores, and RMSD scores provided by the webserver (Roy et al., 2010). Then, Ramachandran plot evaluated the quality of the resultant models by SAVES server (*http://servicesn.mbi.ucla.edu/SAVES/)*. Best models were selected and processed with Discovery Studio (v2016) to dock analysis.

### Antiviral Agents and Structures

A total of 22 known antiviral compounds and structure of GuaHCl (CB6677329), fluoxetine (CB3361058), fluoxetine HCl (CB1335280), TBZE-029 (CB84668594), HBB (CB6375287), MRL-1237(CB14668232), dibucaine (CB1396856), zuclopenthixol (CB1875393), pirlindole (CB0671512), metrifudil (CB0396480), N^6^-benzyladenosine (CB4339416), and hydantoin (CB2352752) were retrieved from *Chemical Book* database (*https://m.chemicalbook.com/)*. The 2D structure of and chemical compounds: thiophene-2-carboxylic acid benzyl-pyridin-2-yl-amide (R523062), 1-(3-phenyl-3-(4-(trifluoromethyl) phenoxy)propyl)guanidine (fluoxetine analog 2b), 2-Butoxy-N-(2-(dimethylamino) ethyl)quinoline-4-carboxamide (dibucaine derivatives 6i), N-(4-Fluorobenzyl)-N-(4-methoxyphenyl)-1H-pyrrole-3-carboxamide (compound 12a), N-(4-Fluorobenzyl)-N-phenyl-1H-pyrrole-3-carboxamide (compound 12b), N-(4-Fluorobenzyl)-N-phenylfuran-2-carboxamide (compound 19b), N-phenyl-N-(4-(trifluoromethyl)benzyl)furan-2-carboxamide (compound 19d), N-(2-(dimethylamino) ethyl)-2-phenylquinoline-4-carboxamide (quinoline analogs 10a), N-[2-(dimethylamino)ethyl]-2-(thiophen-2-yl) quinolone-4-carboxamide (quinoline analogs 12a), and N-[2-(pyrrolidin-1-yl)ethyl]-2-(thiophen-2-yl) quinolone-4-carboxamide (quinoline analogs 12c) were constructed using *ChemDraw* professional software (v20) in Medical Discovery Leader (MDL). *mol* format and were imported for generation of chemical structure.

### Molecular Docking

Ligand and protein optimizations were done using PyMOL (v2.3.3). All water molecules and the co-crystallized compound were removed from the structure. The 22 antiviral chemical ligands were optimized by prepare ligand tool Discovery Studio 2016 (Dassault Systèmes BIOVIA^1^, 2016; El Hassab et al., 2020). The 3D structure was optimized using CHARMm charge force field and further minimized using RMS gradient energy with 0.001 kcal/mol and all the other parameters at default. The docking and active sites of the target protein were analyzed by binding site module of DSv2016. Binding modes were obtained out of which three best volumes were selected for further analysis. A small molecule library was built up and ligand–protein interaction was carried out using LibDock. LibDock possesses the physicochemical properties of the ligands that guide docking according to corresponding features present within the protein-binding sites. The docking analysis revealed that twenty-two chemical compounds as the best scoring molecule that can effectively bind and inhibit the 2C domain. There are several types of interactions that can be monitored in this study by Discovery Studio 2016 (Autumn et al., 2002; Ringer et al., 2007; Bissantz et al., 2010). The categories of interactions are listed in Supplementary Table 1.

### Pharmacokinetic Properties and Drug-Likeliness Prediction

Lipinski's rule of five is one of the significant parameters in drug discovery. The best interacting ligand molecules were subjected to Lipinski's rule (*http://www.scfbio-iitd.res.in/software/drugdesign/lipinski.jsp)*. The absorption, distribution, metabolism, excretion, and toxicity (ADMET) provides absorption, distribution, metabolism, excretion, and toxicity of the given compounds. The pharmacokinetic and pharmacodynamic properties of the selected spice bioactives were evaluated by the ADMETlab2.0 server (*https://admetmesh.scbdd.com/)*. The acquired categorical and numerical values were transformed into qualitative units based on ADMETlab2.0 server explanation and interpretation.

## Results and Discussion

### Homology Modeling of Enterovirus 2C Proteins and Sequence Alignments

**Figure 1:**
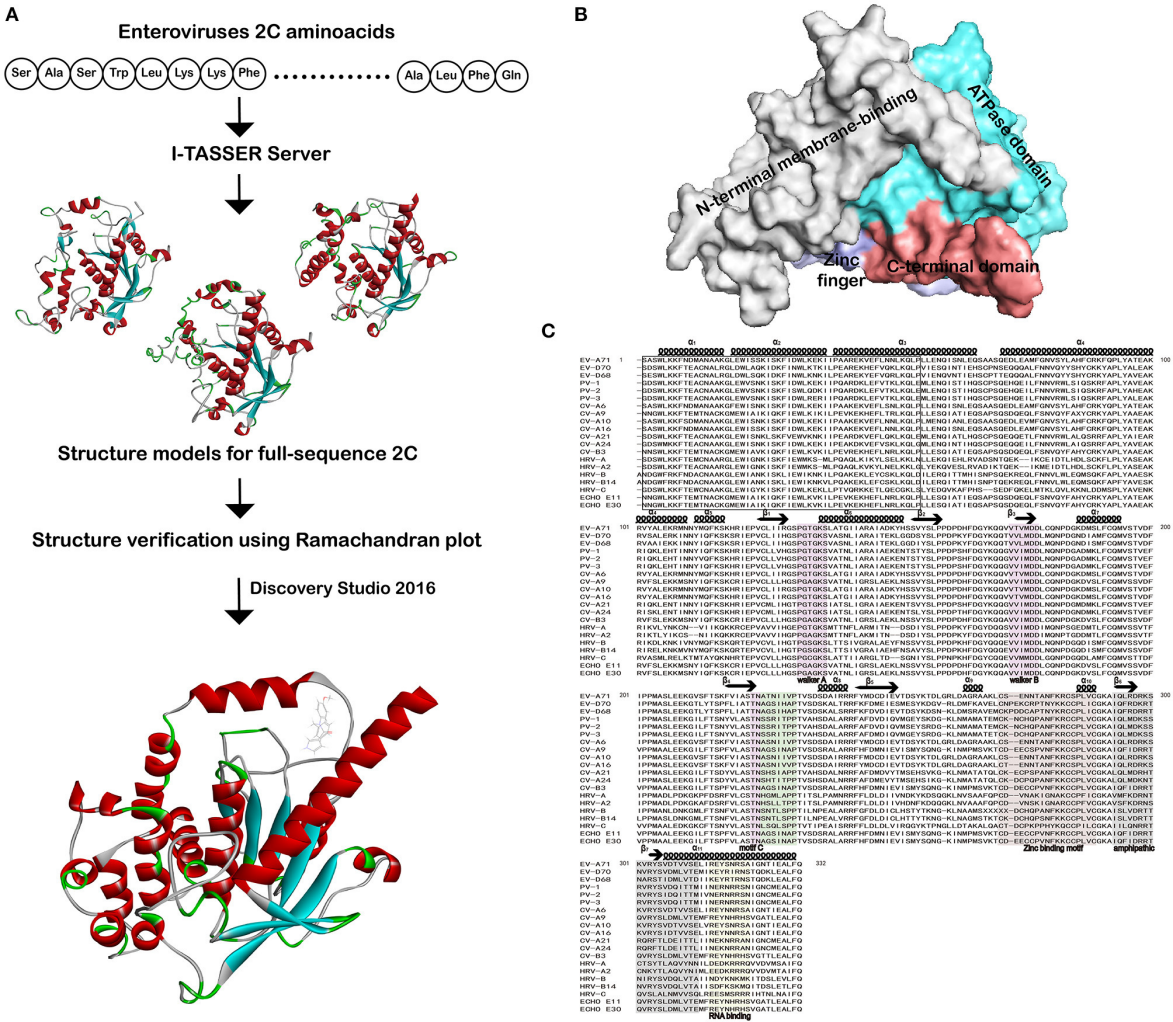
Workflow of homology modeling, docking, and sequences alignment of 2C from enteroviruses. **(A)** A schematic workflow of homology modeling, protein–ligand complex formation by I-TASSER and Discovery Studio. **(B)** Solvent-accessible surface representation of 2C is depicted. The N-terminal membrane-binding domain, ATPase domain, zinc finger domain, and C-terminal domain are shown in the solvent accessible surface representation with gray, aquamarine, light blue and deepsalmon, respectively. **(C)** The 2C was from EV-A71 (ADV76475.1), EV-D70 (QJA10589.1), EV-D68 (AJI77528.1), PV-1 (AAG27168.2), PV-2 (AUF49673.1), PV-3 (AUF49621.1), CV-A6 (QBM01047.1), CV-A9 (AFN25851.1), CV-A10 (QJA28496.1), CV-A16 (AWU78870.1), CV-A21 (AXF50737.1), CV-A24 (ABM54549.1), CV-B3 (AFD33642.1), HRV-A (AFM84630.1), HRV-A2 (QGA30984.1), HRV-B (AFM84628.1), HRV-B14 (NP_041009.1), HRV-C (AET25077.2), Echovirus E11 (CAE12182.1), and Echovirus E30 (AUF49665.1). The secondary structure shown is the predicted by PSIPRED for 2C N-terminal membrane-binding domain. Similarity and alignment calculations were performed using ClustalW. Residue positions form the membrane-binding, RNA-binding, oligomerization, and amphipathic sites are marked with black box. Walker A, Walker B, and Walker C of ATPase domain key residues forming NTP-binding and hydrolysis, Mg^+^-binding and SF3 helicase sites are marked with pinkish purple. Drug-resistant mutant triad regions are in light green. The zinc finger-binding motifs are in khaki. RNA-binding and amphipathic locations of C-terminal domain are in gray and orange, respectively.

The 3D structures of EV 2C proteins are important to understand how 2C proteins perform their function. Protein structures can be determined at high resolution by either experimental methods or computational analysis. In the absence of crystallographic structure, a variety of advanced homology modeling methods have been developed, which can provide reliable models of proteins that share 30% or more sequence identity with a known structure. Such models also have been proven useful during drug design and allowed the taking of key decisions in compound optimization and chemical systems. Figure 1A shows the overall workflow of *I-TASSER* and Discovery Studio (DS) LibDock algorithm. EV 2C structures were submitted to the 2C structure hotspot methods to predict the protein–ligand-binding sites. Each EV 2C structure consists of four subdomains that include N-terminal membrane -binding, ATPase, zinc finger, and C-terminal domain (Figure 1B). The N-terminal domain exhibits α helix structure fold with five (α1 to α5) (Figure 1C). The Walker A is found between β1 and α6, whereas Walker motif B is found between β3 and α7. The SF3 helicase-specific motif C and drug-resistant mutant triad sites are located between β4 and α8. For drug-resistant mutant triad sites of EV 2C, it was previously reported that the putative binding area of (S)-fluoxetine in a homology model of CV-B3 2C, which was based on the published crystal structure of EV-A71 2C and mutational analysis of potential interacting residues (Guan et al., 2017). Triple mutation A224V-I227V-A229V (AVIVAV mutant) gave cross-resistance toward most of the 2C inhibitors (De Palma et al., 2008), as well as the single mutations I227V, C179F, and F190L conferred resistance toward (S)-fluoxetine (Bauer et al., 2019). Sequence alignment analysis was performed which revealed the EV-A71 2C of 116-329 amino acids as a template with 98.13–99.53% for EV-A, 56.60–64.62% for EV-B, 61.32–67.45% for EV-C, 61.97–62.44% for EV-D, and 46.23–56.13% sequence identity for HRV by Clustal Omega (*https://www.ebi.ac.uk/Tools/msa/)* (Figure 1C and Supplementary Table 2). The consistency between each enterovirus 2C sequence and EV-A71 2C template sequence is high, and the similarity varies from 46.23 to 99.53%.

**Figure 2:**
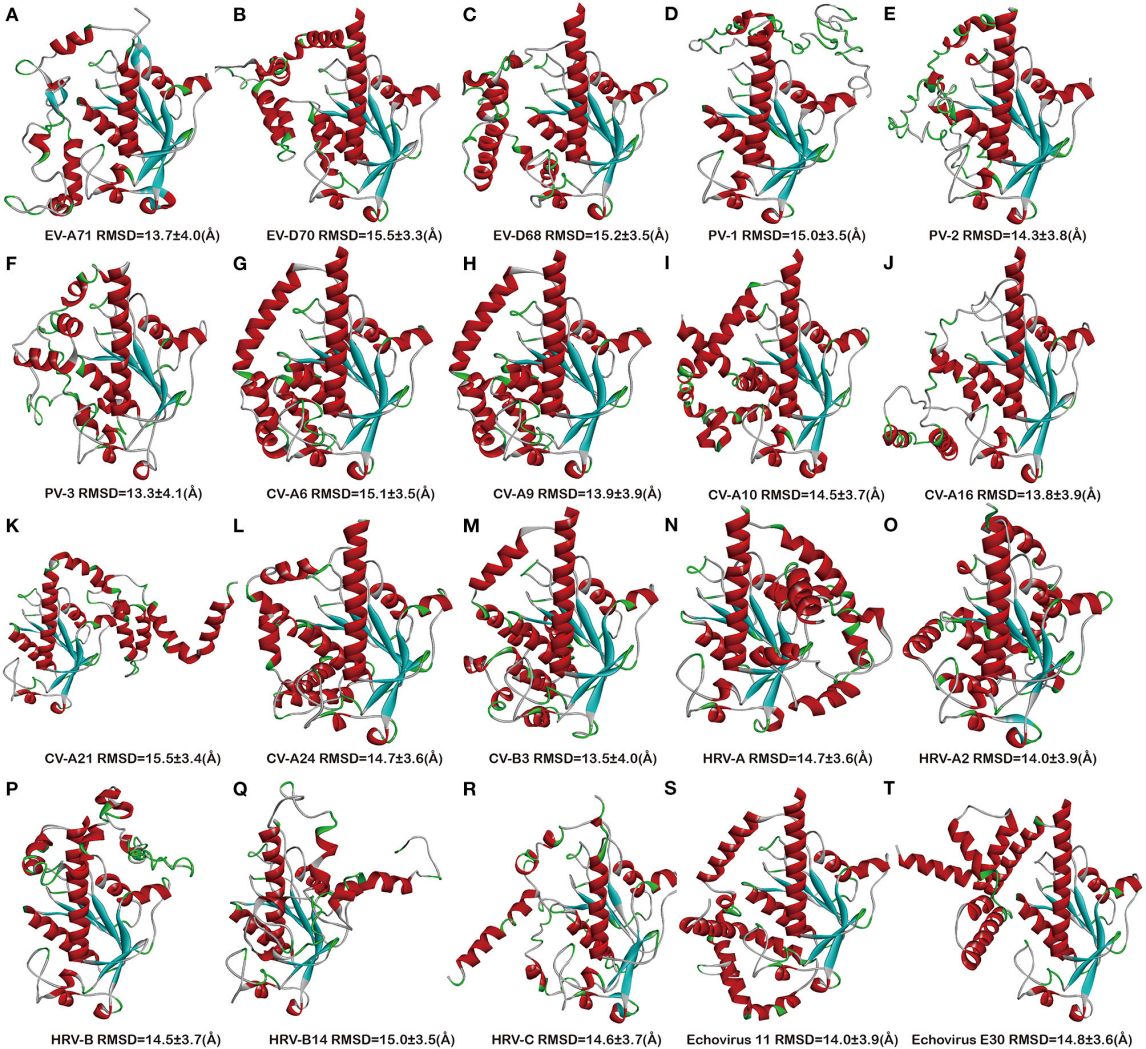
Overview of the 2C protein structures from enteroviruses. **(A)** EV-A71, **(B)** EV-D70, **(C)** EV-D68, **(D)** PV-1, **(E)** PV-2, **(F)** PV-3, **(G)** CV-A6, **(H)** CV-A9, **(I)** CV-A10, **(J)** CV-A16, **(K)** CV-A21, **(L)** CV-A24, **(M)** CV-B3, **(N)** RV-A, **(O)** HRV-A2, **(P)** RV-B, **(Q)** HRV-B14, **(R)** RV-C, **(S)** Echovirus E11, and **(T)** Echovirus E30.

Next, we stimulated the 3D structures of 20 full-length sequence 2C proteins of EVs: EV-A71 (ID: S583037), EV-D70 (ID: S585478), EV-D68 (ID: S584790), PV-1 (ID: S588344), PV-2 (ID: S588735), PV-3 (ID: S588739), CV-A6 (ID: S589030), CV-A9 (ID: S587991), CV-A10 (ID: S589032), CV-A16 (S584480), CV-A21 (ID:S589483), CV-A24 (ID: S589482), CV-B3 (ID: S586681), HRV-A (ID: S585119), HRV-A2 (ID: S589792), HRV-B (ID: S587106), CV-B14 (ID: S589790), HRV-C (ID: S587644), Echovirus E11 (ID: S587655), and Echovirus E30 (ID: S593148) (Figure 2), which were obtained and downloaded from online website through *I-TASSER* programs. The simulated 2C protein structures were applied to the docking study of potential inhibitors, so as to further understand the drug-binding sites and the potential inhibitory effect of inhibitors. The indexes were obtained through C-score, TM-score, and RMSD (Supplementary Table 3). The confidence of each model is quantitatively measured by C-score that is calculated based on the significance of threading template alignments and the convergence parameters of the structure assembly simulations. The C-score is typically in the range of [−5, 2], where the C-score of a higher value signifies a model with a higher confidence. C-score of the models is in the range of [−3.67, −2.89], which indicates a reasonable confidence. The TM-score and RMSD are estimated based on C-score and protein length following the correlation observed between these qualities. The range of TM-score is [0–1]. TM-score varies from 0.21 to 0.52, indicating that the model has correct topology and is reliable. The root mean square deviation (RMSD) ranged from 9.2 to 18.9Å. The root mean square deviation (RMSD) value is closely related to the matching residues (points) between target sequence and template sequence (Li, 2013). High RMSD values (9.2–18.9Å) were obtained. The possible reasons are as follows: N-terminal membrane binding (aa 1-116) of EV 2C proteins accounts for this high variability resulting in weak matching between target sequence and template sequence.

Homology models are imprecise by the definition. Actually, considering the uncertainties involved in homology modeling, it is imperative that the initial 3D model be carefully verified to assess its structural integrity and biological relevance before use in structure-based drug design projects. We used transform-restrained Rosetta (trRosetta) servers (https://yanglab.nankai.edu.cn/trRosetta/) to model the homology structure of full-length sequence of EV 2C protein and verify the results. The trRosetta is an algorithm for fast and accurate protein structure prediction. It builds the protein structure based on direct energy minimizations with a restrained Rosetta. The restraints include inter-residue distance and orientation distributions, predicted by a deep neural network. Homologous templates are included in the network prediction to improve the accuracy further. In benchmark tests on CASP13 and CAMEO-derived sets, trRosetta outperforms all previously described methods. Using the same reference template (PDB entries: 5GQ1 and 5GRB) is used for modeling. The trRosetta results indicated the 116-329 amino acids (aa) modeling structure of 20 EV 2C proteins are relatively consistent with I-TASSER results (Supplementary Figure 1), whereas the amino acid structure of 2C N-terminal membrane-binding domain 1-115aa existed differently. In addition, we evaluated the model of EV 2C protein with ERRAT program by SAVES v6.0 server (https://saves.mbi.ucla.edu/). We found that the overall quality factor by trRosetta results had less weak values than that by I-TASSER. Good high-resolution structures generally produce high values. Previous studies have shown that trRosetta server is weaker than I-TASSER server (Wang and Huang, 2019). We used docking protein–ligand (Genetic Optimization for Ligand Docking (GOLD) modular that is embedded in Discovery Studio, BIOVIA, 2016) to compare with LibDock analysis. This protocol docks ligand using the GOLD program, which uses a genetic algorithm for docking flexible ligands into receptor-binding sites. The simulation needs a lot of time and service cost. We randomly selected two to verify the results. The GOLD analysis shows that N6-benzyladenosine could also bind to EV-A71 2C (binding energy: −48.89 kcal/mol) that in line with LibDock, binding amino acids: PHE8, ASN9, ALA75, MET76, ASN79, HIS85, PHE86, ARG144, ALA145, ASP148, PHE278, LYS279, and ARG280. The binding pose sites are only same in LYS279 and ARG280 with LibDock. For hydantoin, GOLD analysis could bind to PV-1 2C (binding energy: −5.91 kcal/mol), binding amino acids: LYS49, GLN52, GLN59, MET187, LYS188, CYS191, GLN192, SER195, and ARG241. The binding pose sites are only same in LYS49, GLN59, and GLN192 with LibDock. Studies have reported that there are differences between docking methods for accurate poses (Kellenberger et al., 2004). There are three following possible reasons. First, poses are generally inaccurately evaluated when no or very few lipophilic interactions occur between the protein and its ligand. Second, good poses are poorly ranked when the ligand makes no or very few electrostatic interactions (H-bonds or salt bridges) with the protein. Third, docking score is also challenging for complexes whose X-ray structure reveals a mismatch between hydrophobic or electrostatic potentials of the ligand and those of the active site. Thus, herein, it may slightly differ from the peak performance that can be reached by a docking program under optimal conditions.

### Structure Validation of Modeled Protein by Ramachandran Plot

**Figure 3:**
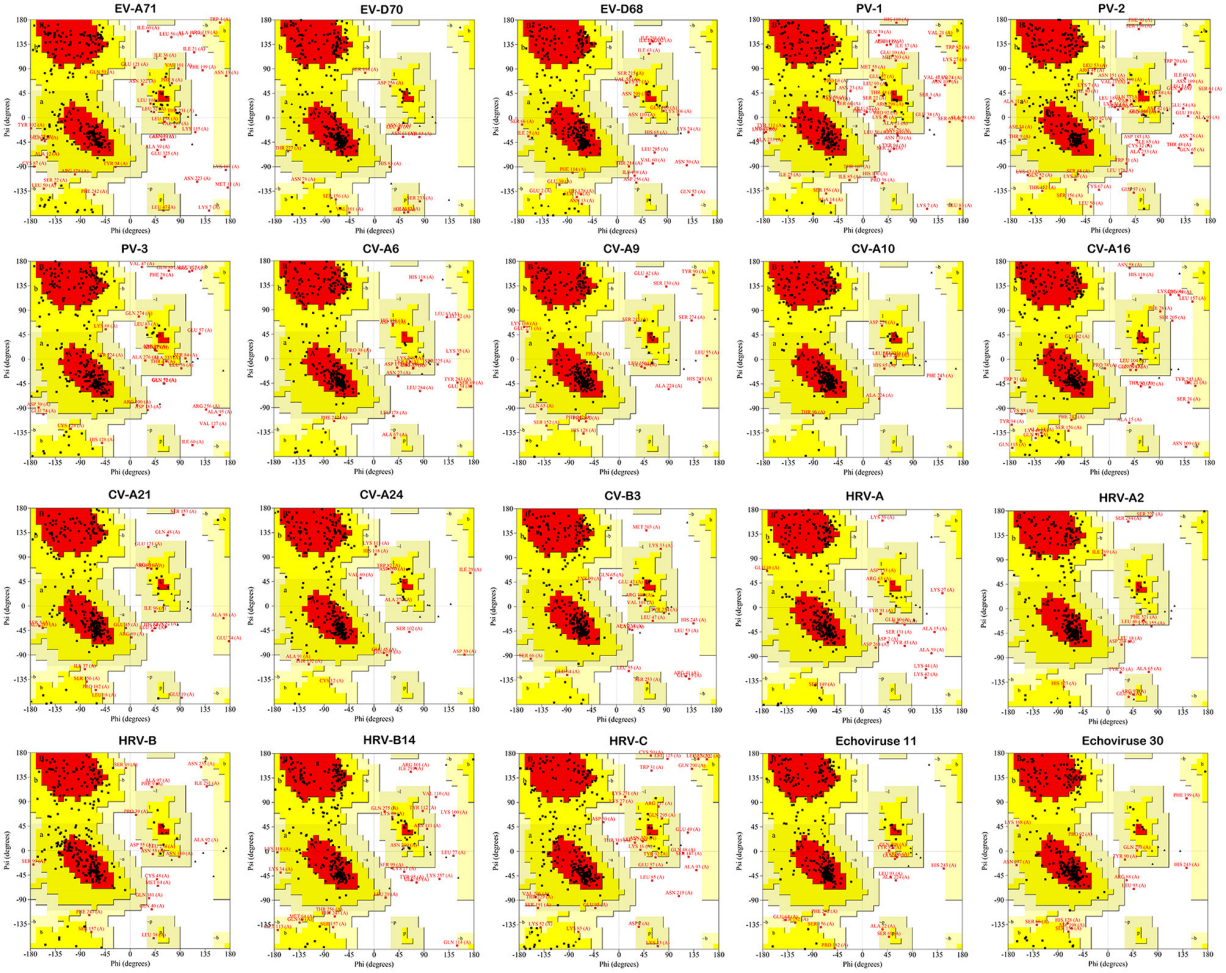
Stereochemical quality of EV 2C protein homology model by Ramachandran plot. The residues occurring red-colored region are in allowed region, and residues in yellow-colored region are in generously allowed.

Ramachandran plot suggested that the amino acid residues of enterovirus 2C protein structure models distributed in the allowable region (red and yellow regions) and the maximum allowable region (light yellow region) accounted for more than 95% of the whole protein except PV-1 and PV-2 (Figure 3). Among them, CV-A10 is the best, reaching 99.7%. PV-1 and PV-2 are 94 and 93.4%, respectively (Supplementary Table 4). Notably, the similarity of the predicted model was evaluated, which affirmed a close homology with the template structure 5GRB (It is from recombinant EV-A71 and comprises amino acid residues 40-329) with an RMSD value of 1.66Å (EV-A71), and 5GQ1 with an RMSD value of 0.48Å (EV-D70), 0.57Å (EV-D68), 0.48Å (PV-1), 0.40Å (PV-2), 0.69Å (PV-3), 1.03Å (CV-A6), 0.59Å (CV-A9), 0.49Å (CV-A10), 1.48Å (CV-A16), 0.56Å (CV-A21), 0.52Å (CV-A24), 0.44Å (CV-B3), 1.21Å (HRV-A), 0.43Å (HRV-A2), 0.52Å (HRV-B), 0.43Å (HRV-B14), 1.60Å (HRV-C), 0.51Å (Echovirus E11), and 0.44Å (Echovirus E30). All the analyses affirmed the reliability of the proposed tertiary structure.

### Drug-Likeness Properties

The investigation of the drug-likeness properties accelerates drug discovery and development procedures. Lipinski's rule-of-five represents the drug-likeness properties (Avdeef and Kansy, 2020). A total of twenty-two drug molecular compounds reported in the literature passed Lipinski's rule-of-five without any violations. These parameters have their principles to identify a bioactive function as an effective drug. The rule of lipophilicity is log*P* ≤ 5, molecular weight: 95.53–400.97 g/mol ≤ 500 Dalton, and the molar refractivity is varied from 40 to 130. Hydrogen bond donors are not more than 5, and there are no more than 10 hydrogen bond acceptors. However, the ADMET property analysis suggests that the inhibitors of novel reported compound 12a, compound 12b, compound 19b, compound 19d, quinoline analogs 12a, dibucaine derivatives 6i, TBZE-029, HBB, MRL-1237, quinoline analogs 10a, zuclopenthixol, fluoxetine, and fluoxetine HCl used have good gastrointestinal absorption in general as evidenced by the F_30%_ and the permeability through the Caco-2 human intestinal cell lines. However, hydantoin and GuaHCl are low (< -5.15 log unit). The P-glycoprotein which is a membrane protein, member of the ATP-binding cassette transporter superfamily, is also known as an important mediator of the efflux of xenobiotics through cells (Kopecka et al., 2020). Several drugs, which include fluoxetine, fluoxetine HCl, compound 12a, quinoline analogs 12c, dibucaine, fluoxetine analogs 2b, pirlindole, and zuclopenthixol, showed a high inhibitory potential on this transporter, thereby indicating that they might interfere with the absorption of drugs.

### Protein-Ligand Docking Analysis

A total of twenty-two inhibitors were docked into EV 2C proteins (EV-A71, EV-D70, EV-D68, PV-1, PV-2, PV-3, CV-B3, CV-A6, CV-A9, CV-A10, CV-A16, CV-A21, CV-A24, HRV-A, HRV-B, HRV-C, HRV-B14, HRV-A2, Echovirus E11, and Echovirus E30) using Discovery Studio LibDock. Among the studied inhibitors, the compound of hydantoin (19 types of EVs) showed the strongest broad-spectrum anti-EV activity. GuaHCL (17 types of EVs), compound 19b (15 types of EVs), R523062 (13 types of EVs), compound 12a (13 types of EVs), compound 12b (13 types of EVs), quinoline analogs 12a (11 types of EVs), compound 19d (12 types of EVs), N6-benzyladenosine (11 types of EVs), dibucaine derivatives 6i (11 types of EVs), TBZE-029 (11 types of EVs), and fluoxetine analogs 2b (11 types of EVs) also exhibited high broad-spectrum antiviral activities with binding to 2C protein (Table 1). In addition, dibucaine, HBB, metrifudil, pirlindole, MRL-1237, quinoline analogs 10a, zuclopenthixol, fluoxetine, and fluoxetine HCl have a weak inhibitory effect on EV 2C protein, respectively. The quinoline analogs 12c displayed the weakest cross-inhibition of EVs and inhibited only EV-D68 and CV-A21. The LibDockscore and free energy of binding energy with 22 compounds binding to EV 2C were ranged from 18.43 to 139.86 and −0.35 to −88.18 kcal/mol. The negative binding energy indicated that those compounds could more easily interact with EV 2C spontaneously. Then, we produced potential inhibitory effect. Generally, the higher the binding-free energy, the more suitable the ligand poses. Apart from VAL118, PHE206, ASP307, and ARG311, hydantoin docked the same binding sites across HRV-A and HRV-A2. In addition, GuaHCL have same binding sites between EV-D68 and EV-D70 except THR221, SER226, and HIS228. This indicates that they may share the same mechanism of action in ATPase domain of 2C protein. All inhibitors mainly bound to the N-terminal membrane-binding and ATPase areas of 2C. In addition to the reported drug-resistant amino acids, we also found many potential affecting drug-binding sites. In the docking-binding modes, diverse interactive and pharmacological action sites of 2C further showed that the drug has a potential cross-inhibitory effect on EVs (Supplementary Figures 2–21).

**Table 1:** Candidate drugs properties screened and Dockscore through LibDock in this study.

**Chem spider ID**	**Compounds**	**Structure**	**Potential treatment**	**Mass (g/mol)**
CB3361058	Fluoxetine	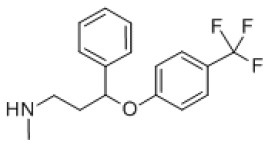	EV-A71,CV-A21,CV-B3,RV-B	309.33
CB6677329	Guanidine hydrochloride	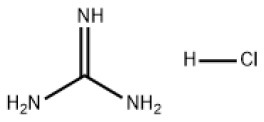	EV-A71,EV-D70,EV-D68,PV-1,PV-3,CV-A6,CV-A9,CV-A16,CV-A21,CV-A24,CV-B3,HRV-A,HRV-A2,HRV-B14,HRV-C,Echovirus E11,Echovirus E30	95.53
CB84668594	TBZE-029	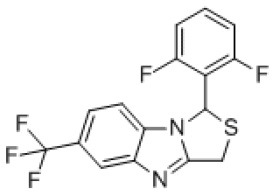	PV-1,PV-3,CV-A6,CV-A10,CV-A16,CV-A21,CV-B3,HRV-B,HRV-C,Echovirus E11,Echovirus E30	356.31
CB2352752	Hydantoin	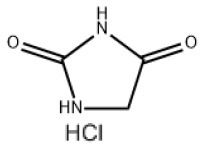	EV-A71,EV-D70,EV-D68,PV-1,PV-2,PV-3,CV-A6,CV-A9,CV-A16,CV-A21,CV-A24,RV-B3,RV-A,HRV-A2,HRV-B,HRV-B14,RV-C,Echovirus E11,Echovirus E30	100.08
CB6375287	HBB	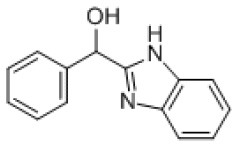	PV-1,PV-2,PV-3,CV-A9,CV-A10,CV-A24,HRV-A2,Echovirus E11,Echovirus E30	224.26
CB1396856	Dibucaine	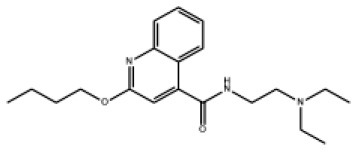	EV-A71,EV-D70,EV-D68,CV-A10,CV-A16,CV-A21,CV-B3,HRV-B,Echovirus E30	343.46
CB0671512	Pirlindole	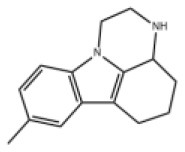	EV-A71,EV-D68,PV-2,PV-3,CV-A16,CV-B3,HRV-B,Echovirus E11	322.42
CB1875393	Zuclopenthixol	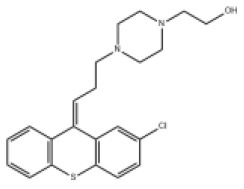	PV-1,PV-3,CV-A10,CV-B3,HRV-C	400.97
CB1335280	Fluoxetine HCl	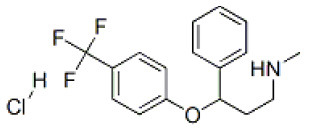	PV-3,CV-A21,CV-B3,HRV-B	345.79
CB14668232	MRL-1237	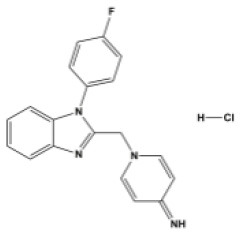	EV-A71,PV-1,CV-A10,CV-A21,CV-A24,CV-B3	354.81
CB0396480	Metrifudil	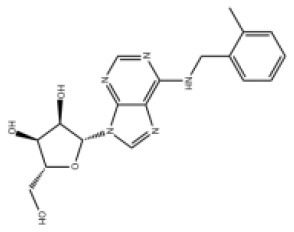	EV-A71,PV-1,PV-2,PV-3,CV-A16,CV-A21,CV-A24,HRV-B,Echovirus E30	371.39
CB4339416	N^6^-benzyladenosine	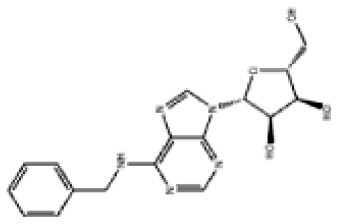	EV-A71,EV-D68,PV-1,CV-A6,CV-A10,CV-A21,HRV-A,HRV-A2,HRV-B,Echovirus E11,Echovirus E30	357.36
-	R523062	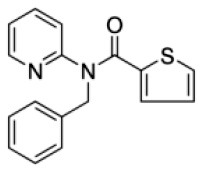	EV-A71,EV-D68,PV-1,PV-3,CV-A6,CV-A10,CV-A21,CV-A24,CV-B3,HRV-A2,HRV-B,HRV-C,Echovirus E30	294.38
-	Dibucaine derivatives 6i	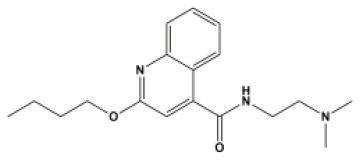	EV-A71,PV-1,PV-2,CV-A6,CV-A10,CV-A21,CV-B3,HRV-A,HRV-B,HRV-C,Echovirus E30	315.42
-	Compound 12a	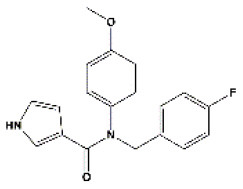	EV-A71,EV-D68,PV-1,PV-2,PV-3,CV-A6,CV-A21,CV-A24,CV-B3,HRV-A2,HRV-B,HRV-B14,Echovirus E30	324.36
-	Compound12b	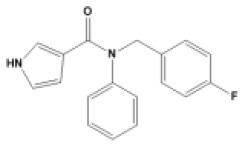	EV-A71,EV-D68,PV-1,PV-2,PV-3,CV-A21,CV-A24,CV-B3,HRV-A,HRV-A2,HRV-B, HRV-B14,Echovirus E30	294.33
-	Compound19b	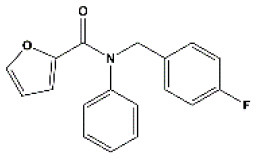	EV-A71,EV-D68,PV-1,PV-2,PV-3,CV-A6,CV-A10,CV-A16,CV-A21,CV-A24,CV-B3, HRV-A2, HRV-B,HRV-B14,Echovirus E30	295.31
-	Compound19d	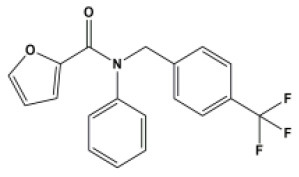	EV-A71,EV-D68,PV-1,PV-2,CV-A10,CV-A21,CV-A24,CV-B3,HRV-A2,HRV-B,HRV-B14,Echovirus E30	345.32
-	Fluoxetine analogs 2b	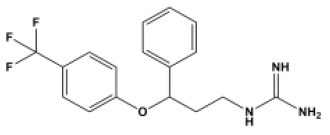	EV-A71,EV-D68,PV-1,CV-A21,CV-B3,RV-A,HRV-A2,HRV-B,HRV-B14,Echovirus E11,Echovirus E30	337.35
-	Quinoline analogs10a	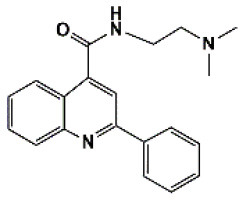	EV-A71,EV-D68,PV-1,CV-A21,HRV-C,Echovirus E30 6	319.41
-	Quinoline analogs12a	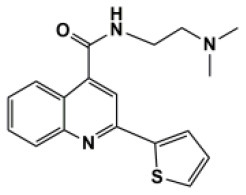	EV-A71,EV-D68,PV-1,PV-2,CV-A16,CV-A21,HRV-A,HRV-A2,HRV-B,HRV-B14,HRV-C,Echovirus E30	325.43
-	Quinoline analogs12c	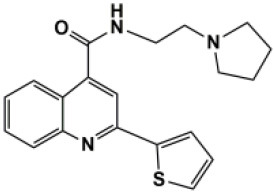	EV-D68,CV-A21	351.47

*-, not available*.

**Figure 4:**
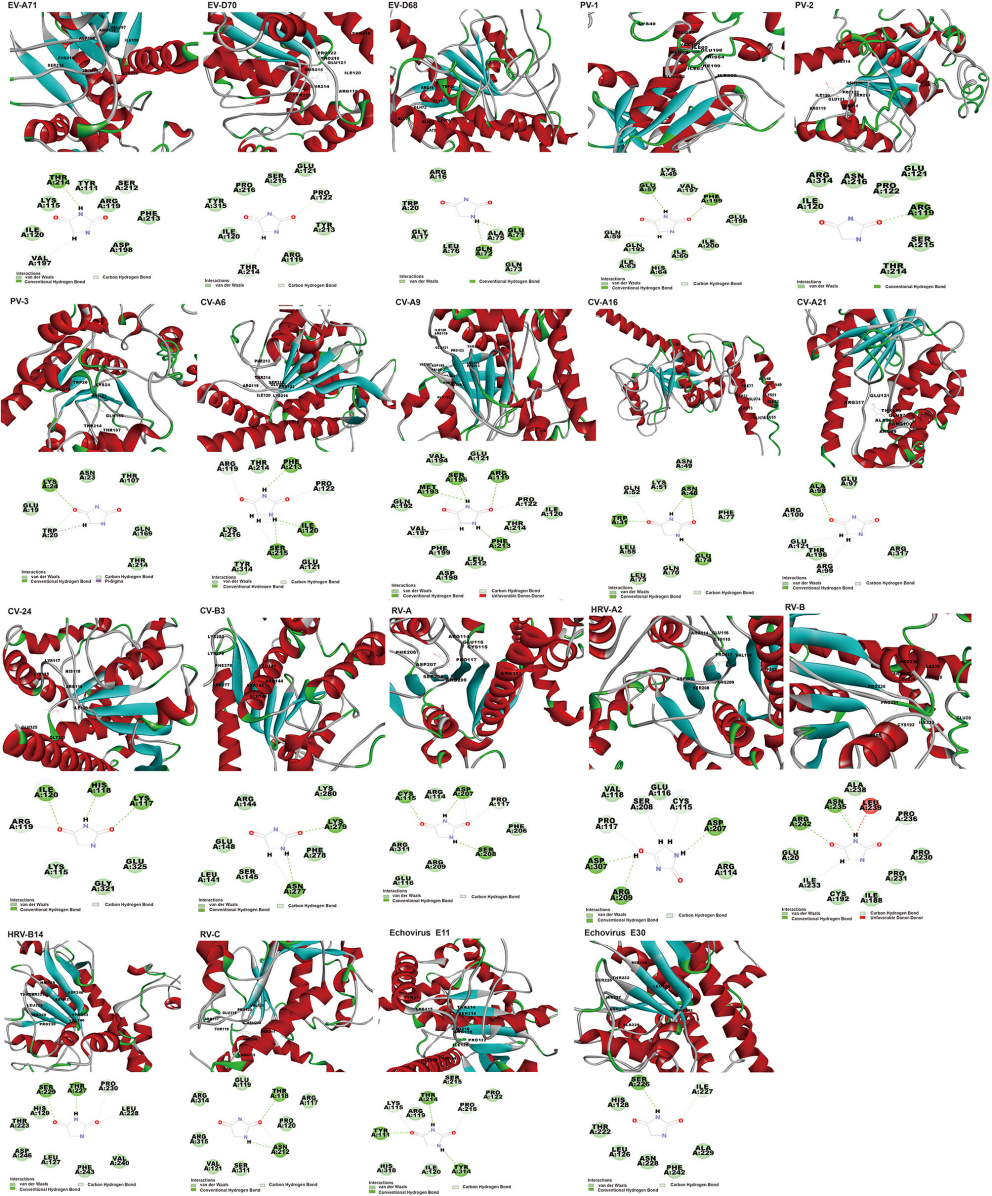
The poses with minimum free energy of the hydantoin compounds along with their corresponding interactions plots within the active site of 2C.

**Figure 5:**
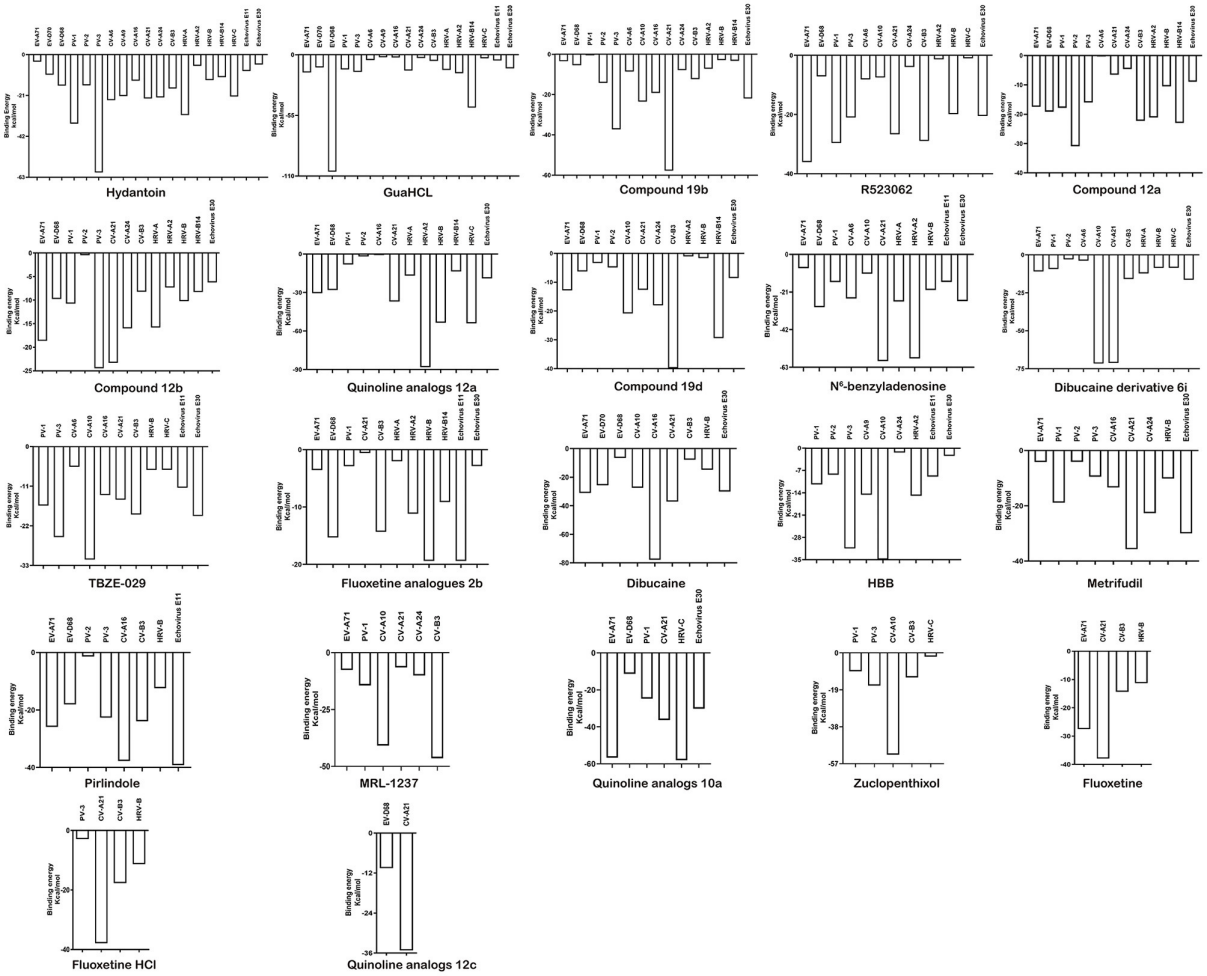
The binding energy of the interactions between EV 2C proteins and 22 compound inhibitors.

Of the twenty EVs, nineteen can interact with hydantoin, binding energy ranging from −60.57 to −3.70 kcal/mol (Supplementary Table 5). However, docking scores appear a little low, varying from 41.30 to 65.31. Docked structure of hydantoin binds firmly at the active site of the EV-A71 2C region of the domain ATPase (Figure 4). Although the binding site is not at the traditional EV-A71 2C protein triple mutation ALA224-ILE227-AIA229 (Hu et al., 2020), the results show that THR214 formed conventional hydrogen bond, and VAL197 and SER212 formed carbon-hydrogen bond interactions in this study. The carbon-hydrogen bond interactions are considered hydrogen bonds where the donor is a polarized carbon atom. A carbon atom is considered to be a donor if it is either in an acetylene group or if it is adjacent to an oxygen or nitrogen atom (Pierce et al., 2002). It also plays an important role in the binding interaction of EV 2C protein and ligands. In the previous experiments, a derivative of hydantoin of compound 5-(3,4-dichlorophenyl) methylhydantoin could effectively inhibit PV-1, PV-2, PV-3, CV-A21, CV-B3, and HRV-14, which was consistent with the results in our study (Vance et al., 1997). In addition, the binding energy between hydantoin and 2C of poliovirus [PV-3 (−60.57 kcal/mol) >PV-1 (−35.50 kcal/mol) >PV-2 (−15.78 kcal/mol)] (Figure 5) was consistent with the plaque formation experiments. Besides the reported viruses, it can also potentially inhibit EV-A71, EV-D68, EV-D70, CV-B3, CV-A6, CV-A9, CV-A16, CV-A24, HRV-A, HRV-A2, HRV-B, HRV-C, Echovirus E11, and Echovirus E30. In addition, the higher binding affinity of hydantoin is attributed to the multiple non-covalent interactions such as *van der Waals* with other amino acid residues (Supplementary Table 6) at the active site of EV-A71 2C. In molecular physics, the *van der Waals* force is a distance-dependent interaction between atoms or molecules. *Van der waals* interactions are classified as the London dispersion forces between “instantaneously induced dipoles,” Debye forces between permanent dipoles and induced dipoles, and the Keesom force between permanent molecular dipoles whose rotational orientations are dynamically averaged over time (Autumn et al., 2002). They differ from covalent and ionic bonds since they are caused by correlations in the fluctuating polarizations of nearby particles. Unlike ionic or covalent bonds, these attractions do not result from a chemical electronic bond and they are comparatively weak and therefore more susceptible to disturbance. Despite being the weakest of the weak chemical forces, it may still support an integral structural load when multitudes of interactions between EV 2C protein and the ligands are present. For Echovirus E30, hydantoin docked mainly between β4 and α3 of ILE227-AIA229 sites, and with residues of ATPase domain. ILE227 can form carbon-hydrogen bond, and SER226 formed conventional hydrogen bond interactions in this study. The hydantoin-binding sites of EV-D68, CV-A16, CV-A21, PV-1, and PV-3 are mainly in the α-helix of 2C N-terminal membrane-binding domain. It may weaken the viral infection and proliferation by interfering the localization and function of 2C protein. Hydantoin binds to EV-D68 firmly at the active site residues ARG16, GLY17, TRP20, GLU71, GLN72, GLN73, ALA75, and LEU76. They formed non-covalent interactions and conventional hydrogen bond with GLU71 and GLN72. The conventional hydrogen bond interactions were the most important factors for the specific binding of ligand to its receptor (Wang et al., 2002). It can exist between a hydrogen bond donor atom and an acceptor atom which are the stronger forces than *van der Waals* interactions (Sweetman et al., 2014). It strengthens the binding energy between drug ligand and EV-D68 2C protein. CV-A16 formed non-covalent interaction with *van der Waals* residues ASN49, LYS51, GLN52, LEU55, GLN70, LEU73, GLU74, and PHE77, conventional hydrogen bond with TRP31, ASN48, and GLU74, and carbon-hydrogen bond with GLU52. CV-A21 formed non-covalent interaction with *van der Waals* residues GLU97, THR196, and ARG317, conventional hydrogen bond with ALA98, and carbon-hydrogen bond with ARG99, ARG100, and GLU121. PV-1 formed non-covalent interaction with *van der Waals* residues LYS49, ILE60, ILE63, HIS64, GLN192, VAL197, GLU198, and ILE200, conventional hydrogen bond with GLU57 and PHE199, and carbon-hydrogen bond with GLN59. PV-3 formed non-covalent interaction with *van der Waals* residues GLU19, ASN23, THR107, GLN169, and THR214, conventional hydrogen bond with LYS24, and carbon-hydrogen bond and Pi-sigma with TRP20. The Pi-sigma contact belongs to hydrophobic interactions between a hydrogen and a Pi ring system, so as to improve the solubility and drug performance of hydrophobic (Udofia et al., 2021). The important interacting residues in the ligands and 2C active sites for other EV-D70, PV-2, CV-B3, CV-A6, CV-A9, CV-A24, HRV-A, HRV-B, HRV-C, RV-B14, HRV-A2, and Echovirus E11 are listed in Supplementary Table 6.

**Figure 6:**
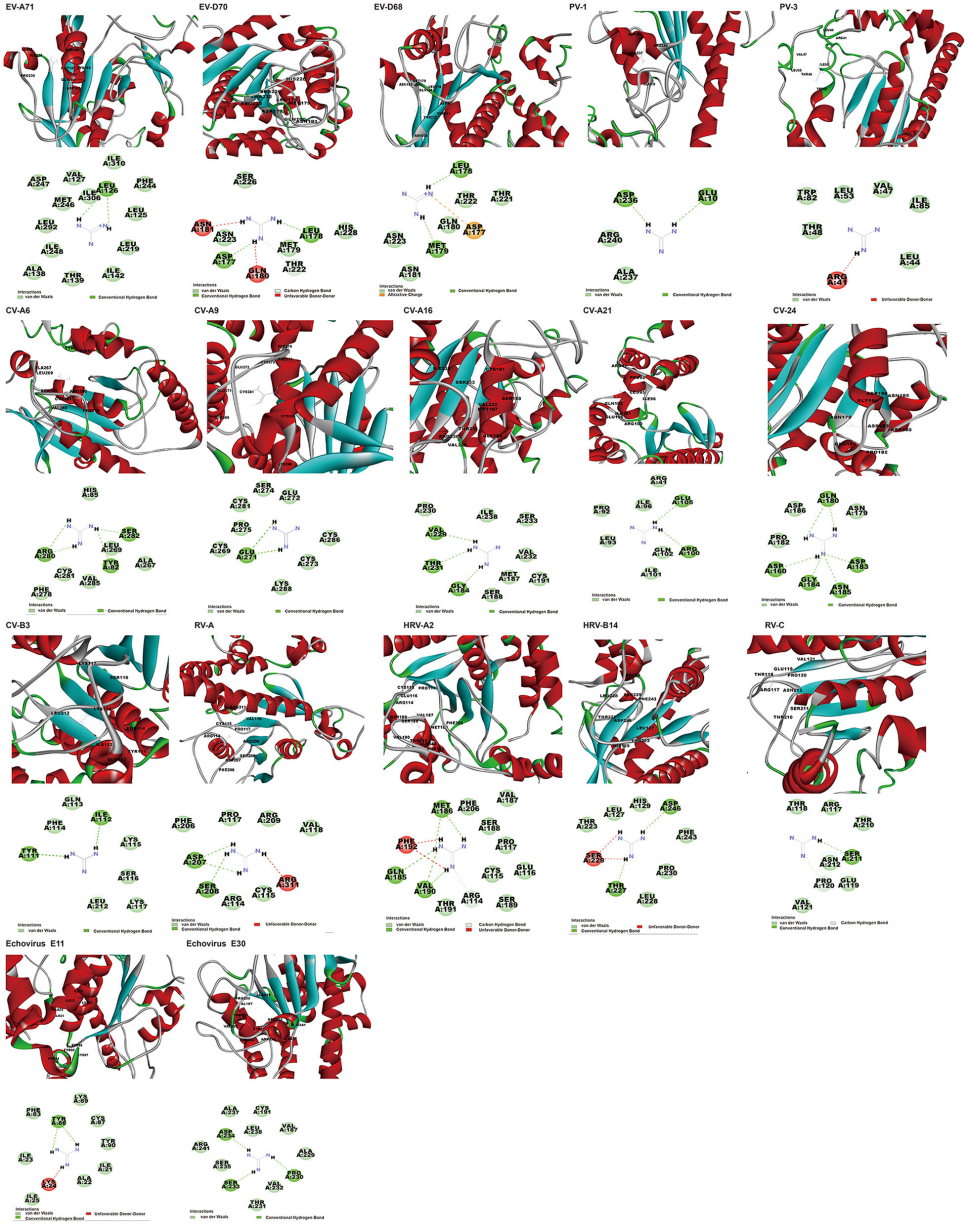
The poses with minimum free energy of the GuaHCL compounds along with their corresponding interactions plots within the active site of 2C proteins.

In addition to the reported PV-1, PV-3, and CV-B3 (Murray and Nibert, 2007; Bauer et al., 2019), GuaHCL was also found to have potential antiviral effect on other enteroviruses: EV-A71, EV-D68, EV-D70, CV-A6, CV-A9, CV-A16, CV-A21, CV-A24, HRV-A, HRV-C, HRV-B14, HRV-A2, Echovirus E11, and Echovirus E30 in this study. Although the docking score is low, its binding energy varies from −106.14 to −2.31 kcal/mol (Supplementary Table 5 and Figure 5), which exhibited a strong binding affinity. The dock score of GuaHCL binding at the active sites of the 2C and their important interactions with various amino acid residues is summarized in Figure 6 and Supplementary Table 6. Previous studies have shown that ILE142, ALA143, Asn179, MET187, SER225, ILE227, ALA233, ALA224, ILE227, and ALA229 were targeted the PV and CV-B3 of 2C. Here, it is found that GLU10, ARG41, LEU44, VAL47, THR48, LEU53, TRP82, ILE85, TYR111, ILE112, GLN113, PHE114, LYS115, SER116, LYS117, LEU212, ASP236, ALA237, and ARG240 may be involved in the interaction between 2C protein and GuaHCL. For EV-A71, EV-D68, EV-D70, CV-A6, CV-A9, CV-A16, CV-A21, CV-A24, HRV-A, HRV-C, RV-B14, HRV-A2, Echovirus E11, and Echovirus E30, we found that the N-terminal domain and ATPase domain of 2C are the major targets for GuaHCL.

Recently, Ma et al. (2020) reported a new small molecule compound, which can effectively bind 2C protein and inhibit the replication and proliferation of EV-D68. However, its target needs to be further studied. The docking studies have shown that R523062 can be used as the potential drugs for infection treatment of EV-A71, EV-D68, PV-1, PV-3, CV-A6, CV-A10, CV-A21, CV-A24, RV-B3, HRV-A2, HRV-B, HRV-C, and Echovirus E30, based on the estimated binding energy (ΔG) value, followed by EV-A71 (−36.08 kcal/mol) > PV-3 (−21.01 kcal/mol) > CV-B3 (−28.92 kcal/mol) > CV-A21 (−26.68 kcal/mol) > PV-3 (−21.01 kcal/mol) > Echovirus E30 (−20.47 kcal/mol) > HRV-B (−19.90 kcal/mol) > CV-A6 (−8.09 kcal/mol) > CV-A10 (−7.43 kcal/mol) > EV-D68 (−7.05 kcal/mol)> CV-A24 (−3.90 kcal/mol) > HRV-A2 (−1.33 kcal/mol) > HRV-C (−0.95 kcal/mol) (Figure 5).

Bauer et al. (2020) have reported that four compounds (12a, 12b, 19b, and 19d) showed broad-spectrum EV and RV activities and inhibited contemporary strains of emerging EV-A71, CV-A24, and EV-D68. The targets of these compounds and the regions producing drug-resistant mutations were mainly in, or adjacent to, the α2 helix of 2C. We further speculated on potential drug-binding target sites. We found that four compounds (19b, 12b, 12a, and 19d) showed broad-spectrum EV activity (EV-A71, EV-D68, PV-1, CV-A24, CV-B3, HRV-A2, and HRV-B14) that was consistent with previous reports and mainly targeted ATPase domain (Supplementary Figures 3–5, 11). It also has potential inhibitory effect on other enteroviruses. For the novel potent enterovirus 2C helicase inhibitors compound dibucaine derivatives 6i, we also found same PRO159 and ASP177 sites of EV-A71 consistent with previous reports (Tang et al., 2020). Besides the two conventional hydrogen bonds ASP160 and ASP186, carbon-hydrogen bond interaction was observed in the residue GLN180. Also, a *van der Waals* interaction was determined in the domain of ATPase (ASP160, PRO161, CYS179, ASN181, PRO182, ASP183, GLY184, LYS185, ASP186, and MET187), which might make a contribution to the enhanced binding force and inhibitory activity. Docking results showed that dibucaine derivative 6i also had binding energy and potential inhibitory effects on PV-1, PV-2, CV-A6, CV-A10, CV-A21, CV-B3, HRV-A, HRV-B, HRV-C, and Echovirus E30 (Figure 5). Besides EV-D68, quinoline analogs compound 12a also showed inhibitory effects on other 11 enteroviruses (EV-A71, EV-D68, PV-1, PV-2, CV-A16, CV-A21, HRV-A, HRV-A2, HRV-B, HRV-B14, HRV-C, and Echovirus E30) (Musharrafieh et al., 2019). Quinoline analogs 10a showed inhibitory effects on CV-A21, Echovirus E30, PV-1, EV-A71, and HRV-C apart from EV-D68. Quinoline analogs 12c only showed inhibitory effects on EV-D68 and CV-A21. In drug targets, we found 2C ILE227 that involves Pi-alkyl hydrophobic interaction, which leads to favorable protein–ligand interactions (Venugopal et al., 2020). Furthermore, the missing amino acid residues (1-116) were also found in pose of quinoline analogs (12a, 10a, and 12c) showing various non-covalent interactions targeted for 2C (Supplementary Table 6 and Supplementary Figures 6, 17, 21).

For fluoxetine and its derivatives, it not only has potential drug-binding effect on CV-B3 (Zuo et al., 2012), but fluoxetine analog 2b shows potential therapeutic effect on extra enteroviruses EV-A71, EV-D68, PV-1, CV-A21, HRV-A, HRV-A2, HRV-B, HRV-B14, Echovirus E11, and Echovirus E30 (Supplementary Figure 10). Among them, fluoxetine analog 2b showed better broad-spectrum resistance than fluoxetine (Supplementary Figure 19) and fluoxetine HCL (Supplementary Figure 20), that is consistent with previous reports (Manganaro et al., 2020). The binding region is mainly located in the N-terminal membrane-binding domain and ATPase domain of 2C (Supplementary Table 6).

In reported dibucaine, TBZE-029, HBB, metrifudil, MRL-1237, N^6^-benzyladenosine, pirlindole, and zuclopenthixol, we found that dibucaine can effectively bind to the key sites of ILE227 and ALA229 of 2C. The interactions between dibucaine and EV-A71 2C involve carbon-hydrogen bond (MET187), Pi-sigma and alkyl (VAL232), Pi-alkyl (CYS191, PRO230 and ILE238), and *van der Waals* (CYS179, ASP183, GLY184, SER188, ILE227, ILE228, ALA229, THR231, SER233, ASP234, and ARG241). Besides EV-A71, EV-D68, and CV-B3 (Ulferts et al., 2016), dibucaine can also effectively interact with CV-A21, CV-A16, Echovirus E30, CV-A10, EV-D70, and HRV-B. Besides studied CV-B3(De Palma et al., 2008), it is suggested that TBZE-029 is effective binding to PV-1, PV-3, CV-A6, CV-A10, CV-A16, CV-A21, RV-B, RV-C, Echovirus E11, and Echovirus E30. HBB showed a good inhibitory effect on poliovirus (PV-1, PV-2, and PV-3) and could also bind to CV-A9, CV-A10, CV-A24, HRV-A2, Echovirus E11, and Echovirus E30.

As previously reported, we also found that metrifudil and N^6^-benzyladenosine have anti-EV71 activity (Arita et al., 2008). The binding energy was −4.13 kcal/mol (LibDockscore:89.99) and −7.70 kcal/mol (LibDockscore:122.59), respectively (Figure 5). The interactions of amino acid sites that mainly involved with LEU5, LYS7, PHE8, ASN9, PHE28, LYS33, ILE36, GLU45, LEU47, LEU53, LEU56, GLU57, ASN58, ILE60, SER61, GLU64, MET76, ASN79, HIS85, PHE86, ARG144, ASP148, LEU269, CYS270, SER271, ASN273, PHE278, LYS279, ARG280, CYS281, SER282, and VAL285 distributed in the N-terminal membrane-binding and ATPase domains of 2C. Simultaneously, the docking showed that metrifudil and N^6^-benzyladenosine had the potential binding and inhibitory effects on enterovirus PV-1, PV-2, PV-3, EV-D68, CV-A6, CV-A10, CV-A16, CV-A21, CV-A24, HRV-A, HRV-A2, HRV-B, Echovirus E11, and Echovirus E30.

MRL-1237 could inhibit PV-1 replication by binding ATPase domain of 2C (Shimizu et al., 2000). The interaction for MRL-1237 is also involved with carbon-hydrogen bond (CYS191 and GLN192), Pi-cation (LYS49 and ARG241), Pi-alkyl (VAL197 and ALA237), Pi-sigma (GLN192), and *van der Waals* (PHE46, VAL47, GLU57, GLN59, ILE60, SER195, PHE199, VAL232, HIS234, and LEU238) in this study. In addition, we found that MRL-1237 has potential and inhibitory effects on others: EV-A71, CV-A10, CV-A21, CV-A24, and CV-B3.

Pirlindole is an inhibitor of monoamine oxidase A, an enzyme involved in the metabolism of monoamines, including serotonin, melatonin, adrenaline, and noradrenaline (Boland et al., 2002). Zuclopenthixol is an antagonist of D2 dopamine receptors and used for the treatment of psychotic disorders (Faure et al., 2021). These two compounds showed inhibitory effects on CV-B3 and PV-3 (Ulferts et al., 2016). For zuclopenthixol, it is also an effective binding energy of inhibition of CV-A10 (−52.38 kcal/mol), PV-1 (−9.46 kcal/mol), and HRV-C (−1.99 kcal/mol) (Figure 5). Pirlindole was found that may act as a novel disincentive to EV-A71 (−25.90 kcal/mol), EV-D68 (−18.03 kcal/mol), PV-2 (−1.42 kcal/mol), CV-A16 (−37.72 kcal/mol), HRV-B (−12.43 kcal/mol), and Echovirus E11(−39.18 kcal/mol) (Figure 5).

Although some of the top-ranked drugs such as compounds 12a, 12b, 19b, quinoline analogs 12a, fluoxetine, and fluoxetine analogs 2b did not show direct interaction with ALA224-ILE227-AIA229, these drugs fitted well into the same binding pocket and showed good binding affinity because of direct or indirect kinds of interactions with other binding site residues (Figure 5). This indicates the importance of other amino acids as well. Other types of interactions or indirect prevailed by these drugs for their stabilization into the ATPase-binding pocket are water-mediated H-bonding, polar, electrostatic, and hydrophobic interactions. Altogether, the homology modeling of EV 2C protein structure may serve as a starting point for further medicinal chemistry guided through structure-based drug design tools, which may lead to the discovery of novel candidate leads for the EV treatment.

## Conclusions

Until now, there is still no drug that can effectively control the occurrence and development of enteroviruses, mainly symptomatic treatment and supportive treatment. Experimental verifications with *in vivo* assays are currently in progress to test the inhibitors targeting to 2C protein. One of the obstacles is that the full-length crystal structure of 2C protein has not been obtained, which leads to the lack of understanding of the structural information of 2C. Particularly, less information is known about the potential binding pockets and the possible binding modes, which our current work is aiming to address. In this study, we first established the structures of full-length enterovirus 2C proteins. The N-terminal membrane binding of 2C of α helix domain may be an important drug inhibition binding region, whereas ATPase domain has been reported. Some new hits were obtained from focused docking which showed a good combination of blind dockings and focused dockings in molecular approach. The key residues involved in stabilizing the complex or participating in the fusion process were identified. These interactions of hits obtained from docking results with the protein functions of 2C provided a promising approach for drug discovery and drug design to inhibit the virus replication by targeting 2C. Our study showed that the current docking protocol utilizing DSv2016 was a robust strategy with sufficient accuracy. Further experimental work such as determination of catalytic binding activity for more compounds should be performed to validate the current *in silico* study.

## Data Availability Statement

The original contributions presented in the study are included in the article/Supplementary Material, further inquiries can be directed to the corresponding author/s.

## Author Contributions

DL performed the study and drafted the manuscript. LZ conceived the work and modified the manuscript. All authors read and approved the final manuscript.

## Funding

This work was supported by the grants from the National Natural Science Foundation of China [82072270 and 81871663] and Academic Promotion Program of Shandong First Medical University [2019LJ001].

## Conflict of Interest

The authors declare that the research was conducted in the absence of any commercial or financial relationships that could be construed as a potential conflict of interest.

## Publisher's Note

All claims expressed in this article are solely those of the authors and do not necessarily represent those of their affiliated organizations, or those of the publisher, the editors and the reviewers. Any product that may be evaluated in this article, or claim that may be made by its manufacturer, is not guaranteed or endorsed by the publisher.

## Footnotes

^1^BIOVIA, a brand of Dassault Systèmes of the 3D EXPERIENCE platform, develops BIOVIA Discovery Studio 2016 software which provides a scientific collaborative environment for advanced the fields of pharmaceutical sciences, precision medicine and health care in the digital age
