# Supplementary material for: Structure Prediction and Potential Inhibitors Docking of Enterovirus 2C Proteins
Source: Front Microbiol. 2022 Apr 29;13:856574. doi: 10.3389/fmicb.2022.856574 (PMC9100428; doi:10.3389/fmicb.2022.856574)
Supplement: Supplementary file 1 [file Table_1.DOCX]

**Table S1. The criteria of the interactions listed in this study by Discovery Studio.**

| **Type of interactions** | **Criteria** |
| --- | --- |
| *Van der Waals* | *Van der Waals* interaction is there if the distance between atoms or molecules is less than 4angstrom (Å). |
| Conventional Hydrogen Bond | Conventional Hydrogen Bond interaction is there if the distancebetween a hydrogen bond donor atom and an acceptor atom (If both atoms are NorO) is less than 3.4Å. |
| Carbon Hydrogen Bond | Carbon Hydrogen Bond interactions are considered weaker hydrogen bonds where the donor is a polarized carbon atom. It is there if the distance between a hydrogen bond donor atom and an acceptor atom (If both atoms are N or O) is less than 3.8Å. |
| Pi Donor Hydrogen Bond | Pi Donor Hydrogen Bondinteractionis there if the distance between a hydrogen bond donor atom and the centroid of each Pi ring is less than 4.2 Å. |
| Water Mediated Hydrogen Bond, Water Hydrogen Bond | The Water monitor is useful for identifying bridging water molecules (when a water molecule is hydrogen bonded to two different molecules such as a protein and a ligand or two domains of a protein) and to visualize water networks. |
| Pi-Cation | Pi-Cation interactions can exist between a positively charged atom and the electrons of a delocalized Pi system.  The Pi-Cation interaction is there if the distance between the centroid of a Pi ring and cation is less than 5.0 Å. Also, cations are considered to be atoms that have a formal charge of at least +0.5. |
| Pi-Anion | Pi-Anion interactions can exist between a negatively charged atom and the electrons of a delocalized Pi system. The Pi-Cation interaction is there if the distance between the centroid of a Pi ring and cation is less than 5.0 Å. And, anions are considered to be atoms that have a formal charge of at least -0.5. |
| Salt Bridge | Salt Bridge interactions are relatively strong non-bonded interactions between pairs of oppositely charged groups where hydrogen bonding also occurs. Salt Bridge interactionis classified as salt bridges for pairs of atoms closer than 4.1 Å. |
| Pi-Sigma | Pi-Sigma interaction (also called CH-π interaction) is there if the distance between a hydrogen and a Pi ring system is less than 4.1 Å. |
| Pi-Alkyl | Pi-Alkyl interactionis there if the distance between the centroids of a Pi ring and an alkyl group is centroid less than 5.5 Å. |
| Alkyl | Alkyl interaction is there if the distance between groups' centroids of the Alkyl centroid is less than 5.5 Å. |
| Pi-Pi Stacked | Pi-Pi stacked interaction is there if the distance between the centroid of each Pi ring pairwithin the Pi-Pi closest atoms is less than 4.5 Å. The angle ωwithin the Pi-Pi theta Stacked must be between 0° and 50°. The angle γ must be between 0° and 35°. |
| Pi-Pi T-Shaped | Pi-Pi T-Shaped interaction is there if the distance between the centroid of each Pi ring pair within the Pi-Pi closest atoms is less than 4.5 Å. The angle ω withinPi-Pi T-Shapedmust be between 0° and 30°. The angle γ must be not less than55°. |
| Amide-Pi Stacked | Amide-Pi Stacked interactionis there if the distance between the centroid of the amide group and the Pi rings is less than 4.5 Å. |
| Halogen (Fluorine) | Halogen Fluorine interaction is there if the distance between the center of acarbon-bound halogen and Fluorine is less than 3.7 Å. |
| Halogen (Cl, Br, I) | Halogen (Cl, Br, I) interaction is there if the distance between the center of a carbon-bound halogen and Cl, Br, I is less than 3.7 Å. |
| Pi-Sulfur | Pi-Sulfur interaction is there if the distance between each such sulfur atom and the centroid of each Pi ring within the Pi-Sulfur is less than 6.0 Å. |
| Sulfur-X | Sulfur-X interactions are found between divalent sulfur and N, O, or S atoms. Sulfur-X interactions is there if the distance between each such sulfur atom and the centroid of X (N, O, or S atoms) is less than 6.0 Å. |
| Pi-Lone Pair | Pi-Lone Pair interaction is there if the distance between the acceptor and the Pi ring centroid within the Pi-Lone Pair isless than3.0 Å. |
| Metal-Acceptor | Metal-Acceptor interactions are analogous to hydrogen bonds and can exist between metal cations and hydrogen bond acceptors. Metal-Acceptor interaction is there if the distance between metal cations and hydrogen bond acceptors is less than 3.4 Å. |
