# Supplementary material for: Structure Prediction and Potential Inhibitors Docking of Enterovirus 2C Proteins
Source: Front Microbiol. 2022 Apr 29;13:856574. doi: 10.3389/fmicb.2022.856574 (PMC9100428; doi:10.3389/fmicb.2022.856574)
Supplement: Supplementary file 2 [file Table_2.DOCX]

**Table S2. Sequence similarity between EV-A71 and other EV 2C proteins by Clustal Omega.**

| ­Enteroviruses | % Similarity |
| --- | --- |
| EV-D70 | 61.97 |
| EV-D68 | 62.44 |
| PV-1 | 67.45 |
| PV-2 | 66.51 |
| PV-3 | 66.98 |
| CV-A6 | 99.53 |
| CV-A9 | 64.15 |
| CV-A10 | 98.13 |
| CV-A16 | 98.13 |
| CV-A21 | 61.79 |
| CV-A24 | 61.32 |
| CV-B3 | 64.15 |
| HRV-A | 46.23 |
| HRV- A2 | 46.70 |
| HRV-B | 56.13 |
| HRV-B14 | 56.60 |
| HRV-C | 51.66 |
| Echoviruse 11 | 64.62 |
| Echoviruse 30 | 64.15 |
