# Supplementary material for: Structure Prediction and Potential Inhibitors Docking of Enterovirus 2C Proteins
Source: Front Microbiol. 2022 Apr 29;13:856574. doi: 10.3389/fmicb.2022.856574 (PMC9100428; doi:10.3389/fmicb.2022.856574)
Supplement: Supplementary file 3 [file Table_3.DOCX]

| ­Enteroviruses | C-score | TM-score | RMSD(Å) | Stuctural analogs | IDEN | TM-score | RMSD(Å) | Coverage |
| --- | --- | --- | --- | --- | --- | --- | --- | --- |
| EV-A71 | -3.02 | 0.37±0.13 | 13.7±4.0 | 5grbA | 0.972 | 0.614 | 1.66 | 0.641 |
| EV-D70 | -3.67 | 0.31±0.10 | 15.5±3.3 | 5gq1A | 0.592 | 0.621 | 0.48 | 0.624 |
| EV-D68 | -3.57 | 0.32±0.11 | 15.2±3.5 | 5gq1A | 0.597 | 0.620 | 0.57 | 0.624 |
| PV-1 | -3.50 | 0.33±0.11 | 15.0±3.5 | 5gq1A | 0.631 | 0.623 | 0.48 | 0.626 |
| PV-2 | -3.24 | 0.35±0.12 | 14.3±3.8 | 5gq1A | 0.626 | 0.624 | 0.40 | 0.626 |
| PV-3 | -2.89 | 0.39±0.13 | 13.3±4.1 | 5gq1A | 0.626 | 0.620 | 0.69 | 0.626 |
| CV-A6 | -3.55 | 0.32±0.11 | 15.1±3.5 | 5grbA | 0.976 | 0.629 | 1.03 | 0.641 |
| CV-A9 | -3.08 | 0.37±0.12 | 13.9±3.9 | 5gq1A | 0.612 | 0.621 | 0.59 | 0.626 |
| CV-A10 | -3.32 | 0.35±0.12 | 14.5±3.7 | 5gq1A | 0.971 | 0.626 | 0.49 | 0.626 |
| CV-A16 | -3.05 | 0.37±0.13 | 13.8±3.9 | 5grbA | 0.962 | 0.619 | 1.48 | 0.641 |
| CV-A21 | -3.67 | 0.31±0.10 | 15.5±3.4 | 5gq1A | 0.587 | 0.622 | 0.56 | 0.626 |
| CV-A24 | -3.38 | 0.34±0.11 | 14.7±3.6 | 5gq1A | 0.573 | 0.623 | 0.52 | 0.626 |
| CV-B3 | -2.95 | 0.38±0.13 | 13.5±4.0 | 5gq1A | 0.612 | 0.623 | 0.44 | 0.626 |
| HRV-A | -3.42 | 0.34±0.11 | 14.7±3.6 | 5grbA | 0.450 | 0.632 | 1.21 | 0.649 |
| HRV- A2 | -3.16 | 0.36±0.12 | 14.0±3.9 | 5gq1A | 0.459 | 0.634 | 0.43 | 0.637 |
| HRV-B | -3.32 | 0.35±0.12 | 14.5±3.7 | 5gq1A | 0.549 | 0.621 | 0.52 | 0.624 |
| HRV-B14 | -3.49 | 0.33±0.11 | 15.0±3.5 | 5gq1A | 0.549 | 0.622 | 0.43 | 0.624 |
| HRV-C | -3.36 | 0.34±0.11 | 14.6±3.7 | 5grbA | 0.498 | 0.611 | 1.60 | 0.641 |
| Echoviruse 11 | -3.15 | 0.36±0.12 | 14.0±3.9 | 5gq1A | 0.621 | 0.623 | 0.51 | 0.626 |
| Echoviruse 30 | -3.43 | 0.34±0.11 | 14.8±3.6 | 5gq1A | 0.617 | 0.623 | 0.44 | 0.626 |

**Table S3. Supreme models predicted scores by I-TASSER and to match the I-TASSER model to structures in the PDB library.**
