# Supplementary material for: Structure Prediction and Potential Inhibitors Docking of Enterovirus 2C Proteins
Source: Front Microbiol. 2022 Apr 29;13:856574. doi: 10.3389/fmicb.2022.856574 (PMC9100428; doi:10.3389/fmicb.2022.856574)
Supplement: Supplementary file 4 [file Table_4.DOCX]

| ­Enteroviruses | Total AAs | Allowed | Disallowed | Allowed percentage (%) |
| --- | --- | --- | --- | --- |
| EV-A71 | 329 | 315 | 14 | 95.3 |
| EV-D70 | 330 | 328 | 2 | 99.3 |
| EV-D68 | 330 | 317 | 11 | 96.3 |
| PV-1 | 329 | 311 | 18 | 94.0 |
| PV-2 | 329 | 309 | 20 | 93.4 |
| PV-3 | 329 | 315 | 14 | 95.3 |
| CVB-A6 | 329 | 323 | 6 | 98.0 |
| CVB-A9 | 329 | 324 | 5 | 98.3 |
| CVB-A10 | 329 | 328 | 1 | 99.7 |
| CVB-A16 | 329 | 316 | 13 | 95.7 |
| CVB-A21 | 329 | 323 | 6 | 98.0 |
| CVB-A24 | 329 | 327 | 2 | 99.3 |
| CV-B3 | 329 | 320 | 9 | 97.0 |
| HRV- A | 322 | 312 | 10 | 96.6 |
| HRV-A2 | 322 | 316 | 6 | 97.9 |
| HRV-B | 330 | 320 | 10 | 96.7 |
| HRV-B14 | 330 | 319 | 11 | 96.3 |
| HRV-C | 322 | 311 | 11 | 96.2 |
| Echoviruse E11 | 329 | 326 | 3 | 99.0 |
| Echoviruse E30 | 329 | 325 | 4 | 98.7 |

**Table S4. Residues in favored regions by PROCHECK (***https://saves.mbi.ucla.edu/***)**
