# Supplementary material for: Structure Prediction and Potential Inhibitors Docking of Enterovirus 2C Proteins
Source: Front Microbiol. 2022 Apr 29;13:856574. doi: 10.3389/fmicb.2022.856574 (PMC9100428; doi:10.3389/fmicb.2022.856574)
Supplement: Supplementary file 5 [file Table_5.DOCX]

**Table S5. Drugs libdockscore through LibDock in this study.**

1) Dibucaine

| Enteroviruses | Binding energy(Kcal/mol) | Libdockscore | Forcefield |
| --- | --- | --- | --- |
| EV-A71 | -30.9821 | 78.593 | CHARMm |
| EV-D70 | -25.6477 | 84.0603 | CHARMm |
| EV-D68 | -6.5812 | 88.2437 | CHARMm |
| CV-A10 | -27.372 | 110.27 | CHARMm |
| CV-A16 | -77.8928 | 84.1919 | CHARMm |
| CV-A21 | -37.0366 | 99.2701 | CHARMm |
| CV-B3 | -7.8389 | 125.74 | CHARMm |
| HRV-B | -14.7885 | 54.7483 | CHARMm |
| Echovirus E30 | -30.0225 | 83.2728 | CHARMm |

2) Fluoxetine analogue 2b

| Enteroviruses | Binding energy (Kcal/mol) | Libdockscore | Forcefield |
| --- | --- | --- | --- |
| EV-A71 | -3.5517 | 102.266 | CHARMm |
| EV-D68 | -15.3035 | 105.723 | CHARMm |
| PV-1 | -2.8722 | 106.167 | CHARMm |
| CV-A21 | -0.5763 | 101.054 | CHARMm |
| CV-B3 | -14.3135 | 117.945 | CHARMm |
| HRV-A | -2.0091 | 103.852 | CHARMm |
| HRV-A2 | -11.1529 | 94.1579 | CHARMm |
| HRV-B | -19.3672 | 90.5876 | CHARMm |
| HRV-B14 | -9.1189 | 63.1607 | CHARMm |
| Echovirus E11 | -19.3598 | 70.5955 | CHARMm |
| Echovirus E30 | -2.8636 | 85.2941 | CHARMm |

3) Fluoxetine

| Enteroviruses | Binding energy(Kcal/mol) | Libdockscore | Forcefield |
| --- | --- | --- | --- |
| EV-A71 | -27.5318 | 89.7202 | CHARMm |
| CV-A21 | -37.9225 | 82.8401 | CHARMm |
| CV-B3 | -14.3135 | 117.945 | CHARMm |
| HRV-B | -11.313 | 88.417 | CHARMm |

4) Fluoxetine HCL

| Enteroviruses | Binding energy(Kcal/mol) | Libdockscore | Forcefield |
| --- | --- | --- | --- |
| PV-3 | -2.9092 | 42.8204 | CHARMm |
| CV-A21 | -37.9225 | 82.8401 | CHARMm |
| CV-B3 | -17.7377 | 112.699 | CHARMm |
| HRV-B | -11.313 | 88.417 | CHARMm |

5) GuaHCL

| Enteroviruses | Binding energy(Kcal/mol) | Libdockscore | Forcefield |
| --- | --- | --- | --- |
| EV-A71 | -16.2162 | 29.1291 | CHARMm |
| EV-D70 | -11.5413 | 43.0958 | CHARMm |
| EV-D68 | -106.143 | 39.5082 | CHARMm |
| PV-1 | -13.4162 | 18.4267 | CHARMm |
| PV-3 | -15.6101 | 31.9259 | CHARMm |
| CV-A6 | -4.7968 | 33.3717 | CHARMm |
| CV-A9 | -2.3094 | 31.0412 | CHARMm |
| CV-A16 | -2.6146 | 34.8776 | CHARMm |
| CV-A21 | -14.4169 | 27.8963 | CHARMm |
| CV-A24 | -3.0425 | 51.5531 | CHARMm |
| CV-B3 | -5.5159 | 32.1822 | CHARMm |
| HRV-A | -13.8695 | 42.2682 | CHARMm |
| HRV-A2 | -16.74 | 45.069 | CHARMm |
| HRV-B14 | -47.9615 | 27.6924 | CHARMm |
| HRV-C | -3.2685 | 38.2426 | CHARMm |
| Echovirus E11 | -5.2516 | 38.8546 | CHARMm |
| Echovirus E30 | -12.3953 | 43.4879 | CHARMm |

6) HBB

| Enteroviruses | Binding energy(Kcal/mol) | Libdockscore | Forcefield |
| --- | --- | --- | --- |
| PV-1 | -11.3987 | 52.8282 | CHARMm |
| PV-2 | -8.3779 | 101.914 | CHARMm |
| PV-3 | -31.4809 | 100.922 | CHARMm |
| CV-A9 | -14.6034 | 83.0964 | CHARMm |
| CV-A10 | -34.9158 | 86.1105 | CHARMm |
| CV-A24 | -1.3762 | 107.576 | CHARMm |
| HRV-A2 | -14.9417 | 68.6151 | CHARMm |
| Echovirus E11 | -8.9272 | 95.6026 | CHARMm |
| Echovirus E30 | -2.4509 | 81.9235 | CHARMm |

7) Hydantoin

| Enteroviruses | Binding energy(Kcal/mol) | Libdockscore | Forcefield |
| --- | --- | --- | --- |
| EV-A71 | -3.6987 | 60.6675 | CHARMm |
| EV-D70 | -10.3913 | 64.155 | CHARMm |
| EV-D68 | -15.9706 | 48.0105 | CHARMm |
| PV-1 | -35.5032 | 56.8712 | CHARMm |
| PV-2 | -15.7787 | 53.5583 | CHARMm |
| PV-3 | -60.5651 | 47.883 | CHARMm |
| CV-A6 | -23.435 | 55.1134 | CHARMm |
| CV-A9 | -21.2833 | 56.4069 | CHARMm |
| CV-A16 | -13.415 | 53.2258 | CHARMm |
| CV-A21 | -22.5263 | 45.3807 | CHARMm |
| CV-A24 | -22.0573 | 41.2966 | CHARMm |
| CV-B3 | -17.4393 | 54.3264 | CHARMm |
| HRV-A | -31.1238 | 65.3118 | CHARMm |
| HRV-A2 | -5.8131 | 53.156 | CHARMm |
| HRV-B | -13.1067 | 52.0698 | CHARMm |
| HRV-B14 | -11.6591 | 42.1455 | CHARMm |
| HRV-C | -21.529 | 52.4491 | CHARMm |
| Echovirus E11 | -8.4872 | 56.6177 | CHARMm |
| Echovirus E30 | -5.1052 | 49.1872 | CHARMm |

8) Compound12a

| Enteroviruses | Binding energy(Kcal/mol) | Libdockscore | Forcefield |
| --- | --- | --- | --- |
| EV-A71 | -17.4693 | 78.4341 | CHARMm |
| EV-D68 | -19.1429 | 117.888 | CHARMm |
| PV-1 | -17.8239 | 81.8148 | CHARMm |
| PV-2 | -30.9531 | 106.159 | CHARMm |
| PV-3 | -16.0299 | 92.8946 | CHARMm |
| CV-A6 | -0.35 | 93.8129 | CHARMm |
| CV-A21 | -6.5558 | 91.0792 | CHARMm |
| CV-B3 | -22.2489 | 114.642 | CHARMm |
| HRV-B | -10.5117 | 78.0505 | CHARMm |
| HRV-B14 | -22.9752 | 66.2268 | CHARMm |
| Echovirus E30 | -8.9495 | 92.3357 | CHARMm |

9) Compound 12b

| Enteroviruses | Binding energy(Kcal/mol) | Libdockscore | Forcefield |
| --- | --- | --- | --- |
| EV-A71 | -18.6606 | 72.1213 | CHARMm |
| EV-D68 | -9.7779 | 85.1334 | CHARMm |
| PV-1 | -10.7518 | 83.3983 | CHARMm |
| PV-2 | -0.4427 | 81.4269 | CHARMm |
| PV-3 | -24.4593 | 86.338 | CHARMm |
| CV-A21 | -23.3191 | 82.0543 | CHARMm |
| CV-A24 | -16.0249 | 81.4029 | CHARMm |
| CV-B3 | -8.2416 | 104.966 | CHARMm |
| HRV-A | -15.8122 | 64.8657 | CHARMm |
| HRV-A2 | -7.3244 | 78.6612 | CHARMm |
| HRV-B | -10.2215 | 73.8197 | CHARMm |
| Echovirus E30 | -6.2455 | 86.1474 | CHARMm |

10) Compound 19b

| Enteroviruses | Binding energy(Kcal/mol) | Libdockscore | Forcefield |
| --- | --- | --- | --- |
| EV-A71 | -3.4432 | 75.1425 | CHARMm |
| EV-D68 | -5.4467 | 89.7952 | CHARMm |
| PV-1 | -0.5593 | 79.7926 | CHARMm |
| PV-2 | -14.2224 | 83.784 | CHARMm |
| PV-3 | -37.2837 | 78.05 | CHARMm |
| CV-A6 | -8.6176 | 92.7559 | CHARMm |
| CV-A10 | -23.479 | 81.3755 | CHARMm |
| CV-A16 | -19.2065 | 75.344 | CHARMm |
| CV-A21 | -57.7773 | 78.2228 | CHARMm |
| CV-B3 | -12.3761 | 90.5128 | CHARMm |
| HRV-B14 | -3.2259 | 70.2034 | CHARMm |
| HRV-B | -2.8516 | 66.2931 | CHARMm |
| Echovirus E30 | -21.9457 | 83.101 | CHARMm |

11) Compound 19d

| Enteroviruses | Binding energy(Kcal/mol) | Libdockscore | Forcefield |
| --- | --- | --- | --- |
| EV-A71 | -12.824 | 79.4657 | CHARMm |
| EV-D68 | -6.2657 | 77.196 | CHARMm |
| PV-1 | -3.3552 | 78.1537 | CHARMm |
| PV-2 | -4.9093 | 83.3394 | CHARMm |
| CV-A10 | -20.8616 | 91.0172 | CHARMm |
| CV-A21 | -12.6583 | 81.332 | CHARMm |
| CV-B3 | -39.8576 | 99.9033 | CHARMm |
| HRV-B | -1.6386 | 70.3662 | CHARMm |
| HRV-B14 | -29.3874 | 60.7408 | CHARMm |
| Echovirus E30 | -8.6068 | 89.537 | CHARMm |

12) Metrifudil

| Enteroviruses | Binding energy(Kcal/mol) | Libdockscore | Forcefield |
| --- | --- | --- | --- |
| EV-A71 | -4.1318 | 89.9945 | CHARMm |
| PV-1 | -18.8334 | 103.171 | CHARMm |
| PV-2 | -4.0954 | 114.28 | CHARMm |
| PV-3 | -9.4574 | 91.8838 | CHARMm |
| CV-A16 | -13.3876 | 77.3614 | CHARMm |
| CV-A21 | -35.7011 | 103.396 | CHARMm |
| CV-A24 | -22.6029 | 114.195 | CHARMm |
| HRV-B | -10.1187 | 80.7697 | CHARMm |
| Echovirus E30 | -29.9616 | 115.291 | CHARMm |

13) MRL-1237

| Enteroviruses | Binding energy(Kcal/mol) | Libdockscore | Forcefield |
| --- | --- | --- | --- |
| EV-A71 | -7.6913 | 76.1991 | CHARMm |
| PV-1 | -14.4108 | 86.3472 | CHARMm |
| CV-A10 | -40.8629 | 98.3146 | CHARMm |
| CV-A21 | -6.5484 | 89.7554 | CHARMm |
| CV-A24 | -10.0959 | 127.131 | CHARMm |
| CV-B3 | -46.433 | 93.0581 | CHARMm |

14) Quinoline analogs 10a

| Enteroviruses | Binding energy(Kcal/mol) | Libdockscore | Forcefield |
| --- | --- | --- | --- |
| EV-A71 | -56.6422 | 66.1358 | CHARMm |
| EV-D68 | -11.3035 | 75.8533 | CHARMm |
| PV-1 | -24.7153 | 87.8322 | CHARMm |
| CV-A21 | -36.2455 | 60.8259 | CHARMm |
| HRV-C | -58.0118 | 65.6073 | CHARMm |
| Echovirus E30 | -30.1275 | 87.5368 | CHARMm |

15) Quinoline analogs 12a

| Enteroviruses | Binding energy(Kcal/mol) | Libdockscore | Forcefield |
| --- | --- | --- | --- |
| EV-A71 | -30.5184 | 87.6718 | CHARMm |
| EV-D68 | -27.987 | 91.1474 | CHARMm |
| PV-1 | -8.1257 | 92.008 | CHARMm |
| PV-2 | -1.8923 | 72.783 | CHARMm |
| CV-A16 | -0.8129 | 80.9258 | CHARMm |
| CV-A21 | -36.9465 | 83.7143 | CHARMm |
| HRV-A | -16.8977 | 95.9293 | CHARMm |
| HRV-A2 | -88.1765 | 94.7369 | CHARMm |
| HRV-B | -53.6069 | 55.9587 | CHARMm |
| HRV-B14 | -13.566 | 66.069 | CHARMm |
| HRV-C | -54.0306 | 79.1399 | CHARMm |
| Echovirus E30 | -18.9365 | 91.0361 | CHARMm |

16) Quinoline analogs 12c

| Enteroviruses | Binding energy(Kcal/mol) | Libdockscore | Forcefield |
| --- | --- | --- | --- |
| EV-D68 | -10.5309 | 86.4501 | CHARMm |
| CV-A21 | -35.1894 | 89.6081 | CHARMm |

17) N^6^-benzyladenosine

| Enteroviruses | Binding energy(Kcal/mol) | Libdockscore | Forcefield |
| --- | --- | --- | --- |
| EV-A71 | -7.6984 | 122.588 | CHARMm |
| EV-D68 | -29.499 | 121.926 | CHARMm |
| PV-1 | -15.4377 | 103.315 | CHARMm |
| CV-A6 | -24.5843 | 94.3033 | CHARMm |
| CV-A10 | -10.8298 | 102.91 | CHARMm |
| CV-A21 | -59.5869 | 109.289 | CHARMm |
| HRV-A | -26.2741 | 128.954 | CHARMm |
| HRV-A2 | -58.093 | 106.692 | CHARMm |
| HRV-B | -19.8761 | 105.13 | CHARMm |
| Echovirus E11 | -15.4164 | 85.5191 | CHARMm |
| Echovirus E30 | -26.0578 | 122.141 | CHARMm |

18) Pirlindole

| Enteroviruses | Binding energy(Kcal/mol) | Libdockscore | Forcefield |
| --- | --- | --- | --- |
| EV-A71 | -25.8967 | 49.3172 | CHARMm |
| EV-D68 | -18.0349 | 79.344 | CHARMm |
| PV-2 | -1.4229 | 61.6671 | CHARMm |
| PV-3 | -22.6848 | 66.5916 | CHARMm |
| CV-A16 | -37.718 | 61.7719 | CHARMm |
| CV-B3 | -23.942 | 82.5111 | CHARMm |
| HRV-B | -12.4276 | 58.4247 | CHARMm |
| Echovirus E11 | -39.1764 | 83.6115 | CHARMm |

19) R523062

| Enteroviruses | Binding energy(Kcal/mol) | Libdockscore | Forcefield |
| --- | --- | --- | --- |
| EV-A71 | -36.0753 | 62.5113 | CHARMm |
| EV-D68 | -7.0474 | 85.3162 | CHARMm |
| PV-1 | -29.6232 | 67.2144 | CHARMm |
| PV-3 | -21.0131 | 75.7279 | CHARMm |
| CV-A6 | -8.0874 | 85.8003 | CHARMm |
| CV-A10 | -7.4326 | 71.4797 | CHARMm |
| CV-A21 | -26.6751 | 79.7579 | CHARMm |
| CV-A24 | -3.8978 | 94.0781 | CHARMm |
| CV-B3 | -28.9161 | 70.6163 | CHARMm |
| HRV-A2 | -1.3306 | 76.5789 | CHARMm |
| RV-B | -19.8988 | 51.4046 | CHARMm |
| HRV-C | -0.9459 | 60.7617 | CHARMm |
| Echovirus E30 | -20.4673 | 69.6916 | CHARMm |

20) Dibucaine derivatives 6i

| Enteroviruses | Binding energy(Kcal/mol) | Libdockscore | Forcefield |
| --- | --- | --- | --- |
| EV-A71 | -10.9067 | 80.5231 | CHARMm |
| PV-1 | -9.3197 | 92.6283 | CHARMm |
| PV-2 | -2.9522 | 84.1158 | CHARMm |
| CV-A6 | -3.8602 | 72.4667 | CHARMm |
| CV-A10 | -71.5551 | 103.764 | CHARMm |
| CV-A21 | -71.1585 | 100.906 | CHARMm |
| CV-B3 | -15.8667 | 102.577 | CHARMm |
| HRV-A | -12.1817 | 99.4951 | CHARMm |
| HRV-B | -8.6397 | 65.8818 | CHARMm |
| HRV-C | -8.5732 | 94.8991 | CHARMm |
| Echovirus E30 | -16.4066 | 94.5382 | CHARMm |

21) TBZE-029

| Enteroviruses | Binding energy(Kcal/mol) | Libdockscore | Forcefield |
| --- | --- | --- | --- |
| PV-1 | -16.3874 | 71.7769 | CHARMm |
| PV-3 | -25.0959 | 86.9829 | CHARMm |
| CV-A6 | -5.6456 | 93.4863 | CHARMm |
| CV-A10 | -31.3599 | 87.8343 | CHARMm |
| CV-A16 | -13.4544 | 51.1749 | CHARMm |
| CV-A21 | -14.7386 | 79.5167 | CHARMm |
| CV-B3 | -18.8795 | 89.1814 | CHARMm |
| HRV-B | -6.4795 | 58.8686 | CHARMm |
| HRV-C | -6.4685 | 139.858 | CHARMm |
| Echovirus E11 | -11.4073 | 95.001 | CHARMm |
| Echovirus E30 | -19.2676 | 102.381 | CHARMm |

22) Zuclopenthixol

| Enteroviruses | Binding energy(Kcal/mol) | Libdockscore | Forcefield |
| --- | --- | --- | --- |
| PV-1 | -9.4639 | 61.1516 | CHARMm |
| PV-3 | -16.8965 | 88.5112 | CHARMm |
| CV-A10 | -52.3841 | 105.773 | CHARMm |
| CV-B3 | -12.6543 | 101.53 | CHARMm |
| HRV-C | -1.9946 | 107.394 | CHARMm |
