# Supplementary material for: Structure Prediction and Potential Inhibitors Docking of Enterovirus 2C Proteins
Source: Front Microbiol. 2022 Apr 29;13:856574. doi: 10.3389/fmicb.2022.856574 (PMC9100428; doi:10.3389/fmicb.2022.856574)
Supplement: Supplementary file 6 [file Table_6.DOCX]

**Table S6. The changes areas of the important interacting residues in the ligands and protease active sites.**

1）Dibucaine

| Enteroviruses | Amino acids |
| --- | --- |
| EV-A71 | CYS179,ASP183,GLY184,MET187,SER188,CYS191,ILE227,ILE228,VAL229,PRO230,THR231,VAL232,SER233,ASP234,ILE238,ARG241 |
| EV-D70 | TYR111,ILE112,PHE114,LYS115,SER118,ARG119,ILE120,LEU150,THR171,THR214,PRO216,TYR315,ASN319 |
| EV-D68 | TRP20,GLN23,LYS24,ILE25,PHE28,PRO54,GLU57,VAL60,ASN61,GLU64,CYS67,THR69,GLN72,GLN73,LEU76 |
| CV-A10 | PRO54,LEU55,MET56,GLU57,ASN58,GLN59,TYR82,LEU83,ALA84,HIS85,PHE86,ARG88,LYS89,PRO92 |
| CV-A16 | LYS24,ILE29,ASP30,TRP31,ASN48,ASN49,GLN52,LEU55,LEU56,GLN59,SER66,GLN70,LEU73,GLU74,PHE77,PRO92 |
| CV-A21 | GLU57,ILE60,ALA61,HIS64,GLN72,GLN73,GLU74,THR75,LEU76,PHE77,ASN78,ASN79,VAL80,TRP82,LEU83,GLN86,PHE90,TYR94,SER235,ASP236,ASN239 |
| CV-B3 | TYR111,ILE112,GLN113,PHE114,LYS115,SER116,LYS117,CYS118,ARG119,ILE120,GLU121,PRO122,LEU212,PHE213,THR214,SER215,PRO216,TYR314,HIS318,GLY321,THR322,GLU325 |
| HRV-B | ILE26,LEU29,ILE30,ILE33,ILE109,TYR112,SER117,GLN119,ARG120,THR121,GLU122,SER197,LYS318,ILE321,THR322,LEU325,GLU326 |
| EchovirusE30 | MET18,TRP20,ILE21,LYS24,ILE25,PHE28,TYR82,PHE83,TYR86,CYS87,MET108,ILE112,ASN151,SER152,SER153,TYR167,LYS168,GLN169,GLN170,ALA171,THR214,SER215,PRO216 |

2）Fluoxetine analogue 2b

| Enteroviruses | Amino acids |
| --- | --- |
| EV-A71 | LEU5,LYS7,PHE8,PHE28,PHE86,ILE141,ARG144,ALA145,ASP148,LYS149,LEU269,ASN277,PHE278,LYS279,ARG280,CYS281,SER282,VAL285 |
| EV-D68 | TRP20,GLN23,LYS24,LYS27,PHE28,PRO54,VAL55,ILE56,GLU57,VAL60,ASN61,GLU64,CYS67, LEU76 |
| PV-1 | LYS49,GLU57,GLN59,ILE60,MET187,LYS188,CYS191,GLN192,SER195,THR196,VAL197,PHE199,ILE200,VAL232,LEU238,ARG241 |
| CV-A21 | GLU57,ILE60,ALA61,HIS64,ASN78,ASN79,TRP82,LEU83,GLN86,PHE90,SER235,ASP236,ASN239,ARG240,HIS296 |
| CV-B3 | TYR111,ILE112,PHE114,LYS115,SER116,LYS117,CYS118,ARG119,ILE120,PRO122,LEU212,THR214,SER215,PRO216,TYR314,HIS318,GLY321,THR322,GLU325 |
| HRV-A | ILE21,LYS24,LYS86,PHE87,LEU90,TYR91,GLU94,ILE98,LEU101,ASN232,ARG233,PHE235,PHE236,LEU237,ASP238,ASP287,ASN289,THR290,GLU306,ARG310 |
| HRV-A2 | LYS112,ARG114,CYS115,GLU116,PRO117,VAL118,ASP144,ASP207,SER208,ARG209,ASP307,ARG311,VAL314,ASP315,THR318 |
| HRV-B | LYS106,ASN107,ILE109,VAL110,MET113,ARG120,THR121,GLN170,GLN171,GLU172,THR215,SER216,ASN217,TYR315,MET319 |
| HRV-B14 | HIS274,GLN275,PRO276,SER277,VAL286,CYS287,GLY288,LYS289,SER303,ASP305,GLN306 |
| Echovirus E11 | GLN26,LYS43,LEU47,LEU50,GLN59,ILE63,PRO68,SER69,GLN70,SER71,GLU74,PRO263,MET264,LYS267 |
| Echovirus E30 | MET18,TRP20,ILE21,LYS24,VAL80,TYR82,PHE83,ALA84,TYR86,CYS87,ARG144,GLU148,ASN151,SER152,SER153,LYS168,GLN169,GLN170 |

3）Fluoxetine

| Enteroviruses | Amino acids |
| --- | --- |
| EV-A71 | PHE8,ASN9,ALA75,MET76,ASN79,LEU83,HIS85,PHE86,ARG144,ALA145,ASP148,LYS149,PHE278,LYS279 |
| CV-A21 | LEU56,GLU57,ILE60,ALA61,HIS64,GLN73,GLU74,ASN78,ASN79,TRP82,LEU83,GLN86,PHE90,TYR94,SER235,ASP236,ASN239,HIS296 |
| CV-B3 | TYR111,ILE112,PHE114,LYS115,SER116,LYS117,CYS118,ARG119,ILE120,PRO122,LEU212,THR214,SER215,PRO216,TYR314,HIS318,GLY321,THR322,GLU325 |
| HRV-B | LEU29,ILE30,ILE33,LYS52,ILE56,ILE109,GLN119,ARG120,THR121,GLU122,SER197,LYS318,ILE321,THR322,LEU325,GLU326 |

4）Fluoxetine HCL

| Enteroviruses | Amino acids |
| --- | --- |
| PV-3 | TRP4,PHE8,ILE25,PHE28,ILE36,ILE37,PRO38,LYS88,PHE90,ARG144,GLU148,ASN151,THR152 |
| CV-A21 | LEU56,GLU57,ILE60,ALA61,HIS64,GLN73,GLU74,ASN78,ASN79,TRP82,LEU83,GLN86,PHE90,TYR94,SER235,ASP236,ASN239,HIS296 |
| CV-B3 | TYR111,ILE112,PHE114,LYS115,SER116,LYS117,CYS118,ARG119,ILE120,VAL197,ASP198,PHE199,LEU212,PHE213,THR214,HIS318 |
| HRV-B | LEU29,ILE30,ILE33,LYS52,ILE56,ILE109,GLN119,ARG120,THR121,GLU122,SER197,LYS318,ILE321,THR322,LEU325,GLU326 |

5）GuaHCL

| Enteroviruses | Amino acids |
| --- | --- |
| EV-A71 | LEU125,LEU126,VAL127,ALA138,THR139,ILE142,LEU219,PHE244,MET246,ASP247,ILE248,LEU292,ILE306,ILE310 |
| EV-D70 | ASP177,LEU178,MET179,GLN180,ASN181,THR222,ASN223,SER226,HIS228 |
| EV-D68 | ASP177,LEU178,MET179,GLN180,ASN181,THR221,THR222,ASN223 |
| PV-1 | GLU10,ASP236,ALA237,ARG240 |
| PV-3 | ARG41,LEU44,VAL47,THR48,LEU53,TRP82,ILE85 |
| CV-A6 | TYR82,HIS85,ALA267,LEU269,PHE278,ARG280,CYS281,SER282,VAL285 |
| CV-A9 | CYS269,GLU271,GLU272,CYS273,SER274,PRO275,CYS281,CYS286,LYS288 |
| CV-A16 | GLY184,MET187,SER188,CYS191,VAL229,PRO230,THR231,VAL232,SER233,ILE238 |
| CV-A21 | ARG41,PRO92,LEU93,ILE96,ARG100,ILE101,GLN102,GLU105 |
| CV-A24 | ASP160,ASN179,GLN180,PRO182,ASP183,GLY184,ASN185,ASP186 |
| CV-B3 | TYR111,ILE112,GLN113,PHE114,LYS115,SER116,LYS117,LEU212 |
| HRV-A | ARG114,CYS115,PRO117,VAL118,PHE206,ASP207,SER208,ARG209,ARG311 |
| HRV-A2 | ARG114,CYS115,GLU116,PRO117,GLN185,MET186,VAL187,SER188,SER189,VAL190,THR191,PHE192,PHE206 |
| HRV-B14 | LEU127,HIS129,THR223,THR227,LEU228,SER229,PRO230,PHE243,ASP246 |
| HRV-C | ARG117,THR118,GLU119,PRO120,VAL121,THR210,SER211,ASN212 |
| Echovirus E11 | ILE21,ALA22,ILE23,LYS24,ILE25,PHE83,TYR86,CYS87,LYS89,TYR90 |
| Echovirus E30 | VAL187,CYS191,ALA229,PRO230,THR231,VAL232,SER233,ASP234,SER235,ALA237,LEU238,ARG241 |

6）HBB

| Enteroviruses | Amino acids |
| --- | --- |
| PV-1 | LYS49,GLN52,GLN59,MET187,LYS188,CYS191,GLN192,VAL232,ALA233,HIS234,ALA237,LEU238 |
| PV-2 | CYS12,ASN13,LYS16,GLY17,LEU18,VAL21,SER22,GLU150,ASN151,THR152,LYS168,GLN169,GLN170,GLY171,ASN216 |
| PV-3 | GLU19,TRP20,ASN23,LYS24,LYS27,ILE29,GLU97,ALA98,LYS103,LEU104,THR107,ILE108,TYR111,LYS168,GLN169,THR214 |
| CV-A9 | VAL40,LYS41,GLU42,LYS43,PHE46,GLN256,ASN257,LYS259,ILE260,ASN261,MET262,PRO263 |
| CV-A10 | VAL101,LEU104,GLU105,MET108,ASN109,MET112,LYS149,TYR150,HIS151,VAL171,LYS216,TYR314 |
| CV-A24 | GIN113,PHE114,LYS115,LYS117,HIS118,ARG119,ILE120,PHE213,THR214,SER215,ASP216,LYS314,ALA318,GLY321,ASN322,GLU325 |
| HRV-A2 | LYS112,ARG114,CYS115,GLU116,PRO117,VAL118,ASP207,SER208,ARG209,ASP307,ARG311,VAL314,ASP315,THR318 |
| EchovirusE11 | MET108,TYR111,LYS115,CYS118,ARG119,ILE120,GLN169,ALA171,THR214,PRO216,TYR314,HIS318 |
| EchovirusE30 | MET18,TRP20,ILE21,LYS24,TYR82,PHE83,TYR86,CYS87,TYR90,VAL101,ASN151,SER152,SER153,LYS168,GLN170 |

7）Hydantoin

| Enteroviruses | Amino acids |
| --- | --- |
| EV-A71 | TYR111,LYS115,ARG119,ILE120,VAL197,ASP198,SER212,PHE213,THR214 |
| EV-D70 | ARG119,ILE120,GLU121,PRO122,TYR213,THR214,SER215,PRO216,TYR315 |
| EV-D68 | ARG16,GLY17,TRP20,GLU71,GLN72,GLN73,ALA75,LEU76 |
| PV-1 | LYS49,GLU57,GLN59,ILE60,ILE63,HIS64,GLN192,VAL197,GLU198,PHE199,ILE200 |
| PV-2 | ARG119,ILE120,GLU121,PRO122,THR214,SER215,ASN216,ARG314 |
| PV-3 | GLU19,TRP20,ASN23,LYS24,THR107,GLN169,THR214 |
| CV-A6 | ARG119,ILE120,GLU121,PRO122,PHE213,THR214,SER215,LYS216,TYR314 |
| CV-A9 | ARG119,ILE120,GLU121,PRO122,GLN192,MET193,VAL194,SER195,VAL197,ASP198,PHE199,LEU212,PHE213,THR214 |
| CV-A16 | TRP31,ASN48,ASN49,LYS51,GLN52,LEU55,GLN70,LEU73,GLU74,PHE77 |
| CV-A21 | GLU97,ALA98,ARG99,ARG100,GLU121,THR196,ARG317 |
| CV-A24 | LYS115,LYS117,HIS118,ARG119,ILE120,GLY321,GLU325 |
| CV-B3 | LEU141,ARG144,SER145,GLU148,ASN277,PHE278,LYS279,LYS280 |
| HRV-A | ARG114,CYS115,GLU116,PRO117,PHE206,ASP207,SER208,ARG209,ARG311 |
| HRV-A2 | ARG114,CYS115,GLU116,PRO117,VAL118,ASP207,SER208,ARG209,ASP307 |
| HRV-B | GLU20,ILE188,CYS192,PRO230,PRO231,ILE233,ASN235,PRO236,ALA238,LEU239,ARG242 |
| HRV-B14 | LEU127,HIS129,THR223,THR227,LEU228,SER229,PRO230,VAL240,PHE243,ASP246 |
| HRV-C | ARG117,THR118,GLU119,PRO120,VAL121,ASN212,SER311,ARG314,ARG315 |
| Echovirus E11 | TYR111,LYS115,ARG119,ILE120,PRO122,THR214,SER215,PRO216,TYR314,HIS318 |
| Echovirus E30 | LEU126,HIS128,THR222,SER226,ILE227,ASN228,ALA229,PHE242 |

8）Compound 12a

| Enteroviruses | Amino acids |
| --- | --- |
| EV-A71 | LEU5,LYS7,PHE8,ASN9,PHE28,ASN79,LEU83,HIS85,PHE86,ARG144,ASP148,LYS149,PHE278,LYS279 |
| EV-D68 | ASN79,TYR82,TYR83,TYR86,CYS87,TYR90,GLU148,LYS149,PRO276,ASN278,TYR279,LYS280,ARG281 |
| PV-1 | LYS49,GLN52,GLU57,GLN59,ILE60,MET187,LYS188,CYS191,GLN192,SER195,VAL197,PHE199,VAL232,LEU238,ARG241 |
| PV-2 | ASN13,GLY17,LEU18,LEU104,THR107,ILE108,ASN109,LYS115,ARG119,ILE120,THR152,GLN169,GLN170,GLU171,THR214,SER215,ASN216,ARG314 |
| PV-3 | TRP4,PHE8,LYS24,ILE25,PHE28,ILE36,PRO38,PHE90,ARG144,ALA147,GLU148,ASN151,THR152,SER153,THR154,GLN170 |
| CV-A6 | TYR82,HIS85,PHE86,LYS89,ILE141,ARG144,ALA145,ASP148,LYS149,LEU269,ASN277,PHE278,LYS279,ARG280,CYS281,SER282,VAL285,VAL307 |
| CV-A21 | PHE90,TYR94,GLU97,ASP236,ASN239,ARG240,PHE242,ALA243,PHE244,ASP245,ASP294,HIS296,THR297 |
| CV-B3 | TYR111,ILE112,PHE114,LYS115,SER116,LYS117,CYS118,ARG119,ILE120,VAL197,ASP198,PHE199,LEU212,PHE213,THR214 |
| RV-B | ILE26,LEU29,ILE30,ILE33,ILE56,GLN60,ILE109,GLN119,ARG120,THR121,GLU122,SER197,LYS318,ILE321,THR322,LEU325,GLU326 |
| HRV-B14 | ASP272,CYS273,HIS274,GLN275,PRO276,SER277,CYS282,VAL286.CYS287,GLY288,LYS289,SER303,ASP305,GLN306,THR309 |
| Echovirus E30 | TRP20,ILE21,LYS24,ILE25,PHE28,PHE83,CYS87,TYR90,VAL101,MET108,ASN151,SER152,SER153,LYS168,GLN170 |

9）Compound 12b

| Enteroviruses | Amino acids |
| --- | --- |
| EV-A71 | MET76,ASN79,ILE141,ARG144,ALA145,ASP148,LYS149,LEU269,ASN277,PHE278,LYS279,ARG280,CYS281,SER282,VAL285 |
| EV-D68 | ILE120,GLU121,PRO122,VAL123,CYS124,VAL194,ARG240,ARG241,LYS243,GLU314,TYR315,THR317,ARG318,THR321 |
| PV-1 | VAL21,LYS24,ILE36,ILE37,PRO38,GLN39,GLU45,PHE46,VAL47,ILE112,GLN113,HIS118,ARG119,THR196 |
| PV-2 | ASN13,GLY17,LEU18,VAL21,SER22,LEU104,THR107,ILE108,ASN109,GLU150,ASN151,THR152,LYS168,GLN169,GLN170,GLU171,THR214, ASN216 |
| PV-3 | LYS24,ILE25,ILE36,SER84,SER87,LYS88,PHE90,ARG144,ALA147,GLU148,ASN151,THR152,SER153,GLN170 |
| CV-A21 | GLU57,ASN58,ILE60,ALA61,HIS64,ASN78,ASN79,TRP82,LEU83,GLN86,PHE90,TYR94,ASP236,ASN239,ARG240,HIS296 |
| CV-A24 | GLN113,PHE114,LYS115,ARG119,ILE120,ASP216,LYS314,ALA318,ASN319,GLY321,ASN322,GLU325 |
| CV-B3 | TYR111,ILE112,PHE114,LYS115,SER116,LYS117,CYS118,ARG119,ILE120,SER196,VAL197,ASP198,PHE199,LEU212,PHE213,THR214 |
| HRV-A | GLU10,PHE28,ILE29,TRP31,ALA39,GLN40,LEU41,TYR45,GLU48,LEU49,LYS50,LEU55 |
| HRV-A2 | LYS112,ARG114,CYS115,GLU116,PRO117,VAL118,ASP207,SER208,ARG209,ASP307,LYS308,ARG311 |
| HRV-B | ILE26,LEU29,ILE30,ILE33,ILE56,GLN60,ILE109,GLN119,ARG120,THR121,GLU122,SER197,LYS318,THR322,LEU325,GLU326 |
| EchovirusE30 | MET18,TRP20,ILE21,LYS24,ILE25,PHE83,TYR86,CYS87,MET108,ASN151,SER152,SER153,LYS168,GLN169,GLN170 |

10）Compound 19b

| Enteroviruses | Amino acids |
| --- | --- |
| EV-A71 | LEU5,LYS7,PHE8,ASN9,PHE28,ASN79,LEU83,HIS85,PHE86,ARG144,ALA145,ASP148,LYS149,PHE278,LYS279 |
| EV-D68 | ILE120,GLU121,PRO122,VAL123,CYS124,VAL194,ARG240,ARG241,LYS243,GLU314,TYR315,THR317,ARG318,THR321 |
| PV-1 | VAL21,LYS24,ILE36,ILE37,PRO38,GLN39,PHE46,VAL47,ILE112,GLN113,HIS118,ARG119,THR196 |
| PV-2 | CYS12,ASN13,LYS16,GLY17,LEU18,VAL21,SER22,ASN109,GLU150,THR152,LYS168,GLN169,GLN170,GLU171,THR214, ASN216 |
| PV-3 | TRP4,PHE8,ILE25,ILE36,ILE37,PRO38,SER84,GLN86,SER87,LYS88,PHE90,ARG144,ALA147,GLU148,ASN151,THR152,SER153,THR154 |
| CV-A6 | TYR82,HIS85,PHE86,LYS89,GLY140,ILE141,ARG144,ALA145,ASP148,LEU269,PHE278,LYS279,ARG280,CYS281,SER282,VAL285 |
| CV-A10 | ASN109,MET112,GLN113,LYS115,ARG119,ILE120,GLN169,PHE213,THR214,SER215,LYS216,TYR314 |
| CV-A16 | LYS16,THR96,GLU97,LYS99,ARG100,ALA103,ARG107,ARG144,ASP148,HIS151,SER152,SER153 |
| CV-A21 | GLU57,ILE60,ALA61,HIS64,ASN78,ASN79,TRP82,LEU83,GLN86,PHE90,TYR94,ASP236,ASN239,ARG240,HIS296 |
| CV-B3 | TYR111,PHE114,SER116,CYS118,ARG119,ILE120,PRO122,YHR214,SER215,PRO216,TYR314,HIS318,GLY321,THR322,GLU325 |
| HRV-B | ILE26,LEU29,ILE30,ILE33,ILE56,GLN60,GLN119,ARG120,THR121,GLU122,SER197,LYS318,ILE321,THR322,LEU325,GLU326 |
| HRV-B14 | ASP272,CYS273,HIS274,GLN275,PRO276,SER277,CYS282,VAL286.CYS287,LYS289,SER303,ASP305,GLN306 |
| Echovirus E30 | MET18,TRP20,ILE21,LYS24,ILE25,PHE83,TYR86,CYS87,MET108,GLU148,ASN151,SER152,SER153,LYS168,GLN169,GLN170 |

11）Compound19d

| Enteroviruses | Amino acids |
| --- | --- |
| EV-A71 | LEU5,LYS7,PHE8,ASN9,PHE28,ASN79,LEU83,HIS85,PHE86,ARG144,ALA145,ASP148,LYS149,PHE278,LYS279,ARG280 |
| EV-D68 | GLU121,PRO122,VAL123,VAL194,ARG240,ARG241,PHE242,LYS243,ARG297,ARG318 |
| PV-1 | VAL47,THR48,LYS49,GLN52,GLU57,GLN59,LYS188,CYS191,GLN192,SER195,VAL197,PHE199,ARG241 |
| PV-2 | ILE25,ILE29,LEU32,GLN33,ASP42,LYS43,LYS49,TRP82,ILE85,LYS88,ARG144,ALA147,GLU148,THR152,SER153,THR154,LYS279 |
| CV-A10 | ASN109,MET112,GLN113,LYS115,SER116,LYS117,ARG119,ILE120,GLN169,THR214,LYS216,TYR314 |
| CV-A21 | GLU57,ILE60,ALA61,HIS64,ASN79,TRP82,LEU83,GLN86,PHE90,TYR94,SER235,ASP236,ASN239,ARG240,HIS296 |
| CV-B3 | MET18,LYS24,LYS27,PHE28,TRP31,LEU32,CYS87,TYR90,ALA91,TYR94,SER153,VAL154,TYR155,SER156,ASP165,GLY166,GLN170 |
| HRV-B | LYS106,ILE109,VAL110,MET113,ARG120,THR121,GLN170,GLU172,THR215,ASN217,TYR315,MET319 |
| HRV-B14 | ASP272,HIS274,GLN275,PRO276,SER277, CYS287,GLY288, LYS289,SER303,ASP305,GLN306 |
| Echovirus E30 | MET18,TRP20,ILE21LYS24,ILE25,PHE83,TYR86,CYS87,TYR90,VAL101,MET108,ASN151,SER152,SER153,LYS168 |

12）Metrifudil

| Enteroviruses | Amino acids |
| --- | --- |
| EV-A71 | LEU5,LYS7,PHE8,ASN9,PHE28,MET76,ASN79,HIS85,PHE86,ARG144,ASP148,LEU269,PHE278,LYS279,ARG280,CYS281,SER282,VAL285 |
| PV-1 | LYS43,PHE46,VAL47,LYS49,GLN52,GLU57,GLN59,GLU121,MET187,LYS188,CYS191,GLN192,VAL194,SER195,THR196,VAL197,VAL232,ARG240,ARG241 |
| PV-2 | GLY17,LEU18,VAL21,SER22,LYS115,ARG119,ILE120,GLU121,PRO122,ASN151,THR152,LYS168,GLN169,GLN170,GLY171,PHE213,THR214,ASN216,ARG314 |
| PV-3 | PRO38,GLN39,ALA40,ARG41,ASP42,LEU44,VAL47,THR48,TRP82,SER84,ILE85,SER87,LYS88,PHE90.SER153 |
| CV-A16 | LEU18,GLU19,TRP20,ILE21,ILE25,MET76,ASN79,VAL80,LEU83 |
| CV-A21 | HIS64,SER69,GLN73,GLU74,ASN78,ASN79,ARG81,TRP82,PHE90,HIS225,SER235,ASP236,ASN239,ASP245,ARG295,HIS296,ARG298 |
| CV-A24 | GLN113,PHE114,LYS115,LYS117,HIS118,ARG119.ILE120,GLU121,YHR196,ASP216,ARG317,ALA318,ILE320,GLY321,ASN322,MET324,GLU325 |
| HRV-B | LYS106,ILE109,VAL110,MET113,GLN114,LYS116,ARG120,THR121,GLN170,GLU172,THR215,SER216,ASN217,TYR315,MET319 |
| Echovirus E30 | ILE21,PHE83,TYR86,CYS87,LYS89,TYR90,ALA91,LEU93,ALA98,VAL101,PHE102,GLU105,GLU148,ASN151,SER152,SER153,LYS168,GLN170,GLU208 |

13）MRL-1237

| Enteroviruses | Amino acids |
| --- | --- |
| EV-A71 | LEU5,LYS7,PHE8,ASN9,PHE28,ALA75,ASN79,LEU83,HIS85,PHE86,ARG144,LYS279,ARG280 |
| PV-1 | PHE46,VAL47,LYS49,GLU57,GLN59,ILE60,CYS191,GLN192,SER195,VAL197,PHE199,VAL232,HIS234,ALA237,LEU238,ARG241 |
| CV-A10 | VAL101,LEU104,GLU105,MET108,ASN109,MET112,TYR150,HIS151,VAL171,LYS216,TYR314,SER315,SER318 |
| CV-A21 | ILE60,HIS64,ASN79,TRP82,LEU83,GLN86,PHE90,TYR94,SER235,ASP236,ASN239,ARG240,HIS296 |
| CV-A24 | GLN113,PHE114,LYS115,LYS117,HIS118,ARG119,ILE120,PRO122,PHE213,THR214,SER215,ASP216,LYS314,ALA318,GLY321,ASN322,GLU325 |
| CV-B3 | GLN26,ILE29,GLU30,LYS33,VAL34,LEU141,ARG144,SER145,GLU148,VAL266,ASN277,PHE278,LYS279,LYS280,CYS282,,LEU284,VAL285 |

14）Quinoline analogs 10a

| Enteroviruses | Amino acids |
| --- | --- |
| EV-A71 | ASN181,PRO182,ASP183,GLY184,LYS185,MET187,SER188,CYS191,PRO230,THR231,VAL232,SER233,ILE238,ARG241 |
| EV-D68 | ILE120,GLU121,PRO122,VAL123,CYS124,VAL194,ARG240,ARG241,PHE242,LYS243,ARG297,GLU314 ,ARG318,THR321 |
| PV-1 | LYS49,MET55,GLU57,GLN59,ILE60,ILE63,HIS64,MET187,LYS188,CYS191,GLN192,SER195,VAL197,GLU198,PHE199,ILE200,VAL232,ALA237,LEU238,ARG241 |
| CV-A21 | ASN239,ARG240,PHE242,ALA243,PHE244,ASP245,MET246,ASP294,HIS296,THR297,GLN299,PHE301,LEU309,ARG316 |
| HRV-C | PHE28,ILE29,GLN71,LYS72,LEU74,MET75,LEU78,ASN149,ILE150TYR151,SER152,LEU153,PRO154,PRO155,MET170,ASP172 |
| Echovirus E30 | GLU10,ASN13,ALA14,GLU19,TRP20,ALA22,ILE23,GLN26,LYS27,GLU30,ILE36,LYS41,GLU45,PHE46,ARG49,GLN52,LEU53 |

15）Quinoline analogs 12a

| Enteroviruses | Amino acids |
| --- | --- |
| EV-D68 | ILE120,GLU121,PRO122,VAL123,CYS124,VAL194,ARG240,ARG241,PHE242,LYS243,ARG297,GLU314,TYR315, ARG318 |
| PV-1 | LYS49,GLU57,GLN59,ILE60,ILE63,HIS64,LYS188,CYS191,GLN192,SER195,GLU197,GLU198,PHE199,ILE200,VAL232,LEU238,ARG241 |
| PV-2 | MET251,CYS269,ASN271,HIS273,GLN274,PRO275,ALA276,CYS281,VAL285,CYS286,GLY287,LYS288,GLN291,SER302,ASP304,GLN305 |
| CV-A16 | TRP20,ILE21,ILE25,ALA75,MET76,GLY78,ASN79,VAL80,TYR82,LEU83,PHE86,LEU137,ILE141,ALA267,LEU269,SER282,LEU284, |
| CV-A21 | GLU57,ILE60,ALA61,HIS64,ASN78,ASN79,ARG81,TRP82,LEU83,GLN86,PHE90,TYR94,SER235,ASP236,ASN239,ARG240 |
| HRV-A | TRP20,ILE21,LYS24,PHE87,LEU90,TYR91,GLU94,ASN232,ARG233,ARG234,PHE235,PHE236,LEU237,ASN289,GLU306,ARG310 |
| HRV-A2 | PRO89,LEU90,GLU94,ALA95,ARG97,ILE98,LEU101,ASP153,PRO154,LYS155,THR176,ASP178,ASP179,THR181,LEU182,GLN185,PHE192,PRO195 |
| HRV-B | ILE26,LEU29,ILE56,GLN60,THR63,ILE66,TYR112,PHE115,SER117,GLN119,SER197,THR322,LEU325,GLU326,PHE329,GLN330 |
| HRV-B14 | LYS28,ILE30,GLU31,LYS34,MET87,SER88,PHE91,ALA92,PHE94,TYR95,ARG120,THR121,GLU122,SER197,ARG241,ASP314,SER317,LYS318,ILE321 |
| HRV-C | PHE28,LEU32,LEU36,GLN71,LEU74,MET75,LEU78,THR138,ASN149,ILE150,TYR151,SER152,LEU153,PRO154,PRO155,MET170 |
| Echovirus E30 | MET18,TRP20,ILE21,LYS24,PHE83,TYR86,CYS87,LYS89,TYR90,ALA91,VAL101,MET108,ASN151,SER152,SER153,LYS168,GLN169,GLN170 |

16）Quinoline analogs 12c

| Enteroviruses | Amino acids |
| --- | --- |
| EV-D68 | GLU10,ASN13,ARG16,ASP19,TRP20,GLN23,LYS24,ILE25,LYS27,PRO54,ILE56,GLU57,GLN59,VAL60,LEU76 |
| CV-A21 | GLN73,PHE90,LEU126,HIS128,THR222,HIS225,SER226,ILE227,ALA228,PRO229,ASP236,ASN239,ARG240,PHE242,ASP245,ARG295,HIS296 |

17）N^6^-benzyladenosine

| Enteroviruses | Amino acids |
| --- | --- |
| EV-A71 | LYS33,ILE36,GLU45,LEU47,LEU53,LEU56,GLU57,ASN58,ILE60,SER61,GLU64,LEU269,CYS270,SER271,ASN273,LYS279,ARG280,CYS281 |
| EV-D68 | PHE46,CYS87,TYR90,ALA91,TYR94,ARG100,ILE104,LYS149,LEU150,GLY151,TYR315,ARG316,ASN319,SER320,ASP323 |
| PV-1 | VAL21,LYS24,PHE28,ILE36,ILE37,PRO38,GLN39,LEU44,GLU45,PHE46,VAL47,ILE112,GLN113,HIS118,THR196,VAL197 |
| CV-A6 | LYS27,TRP31,LYS35,ALA39,ASN79,VAL80,SER81,TYR82,PHE86,LEU137,GLY140,ILE141,ALA263,GLY264,ALA267,LEU284 |
| CV-A10 | PRO54,LEU55,MET56,GLU57,ASN58,GLN59,SER81,TYR82,ALA84,HIS85,PHE86,LYS89 |
| CV-A21 | ASN78,ARG81,TRP82,PHE90,PRO229,SER235,ASP236,ASN239,ARG240,PHE242,ALA243,PHE244,ASP245,ASP294,HIS296,THR297 |
| HRV-A | LYS24,LYS86,PHE87,LEU90,TYR91,GLU94,GLU116,PRO117,VAL118,ALA119,ASN232,ARG233,ARG234,PHE235,PHE236,LEU237,ASP238,ASN289,GLU306,ARG310 |
| HRV-A2 | PRO89,LEU90,GLU94,ARG97,ILE98,LEU101,ASP153,PRO154,LYS155,THR176,ASP178,THR181,LEU182,GLN185,PHE192,PRO195 |
| HRV-B | LYS106,ILE109,VAL110,MET113,GLN114,LYS116,ARG120,THR121,GLN170,GLU172,THR215,ASN217,TYR315,MET319 |
| Echovirus E11 | TRP20,GLN26,ILE29,LEU32,LYS33,ILE36,GLN59,ILE63,PRO68,SER69,GLN70,SER71,MET262,PRO263,VAL266 |
| Echovirus E30 | MET18,TRP20,ILE21,LYS24,VAL80,TYR82,PHE83,ALA84,TYR86,CYS87,ARG144,SER145,ALA147,GLU148,ASN151,SER152,SER153 |

18）Pirlindole

| Enteroviruses | Amino acids |
| --- | --- |
| EV-A71 | SYS179,ASN181,ASP183,GLY184,LYS185,MET187,SER188,ILE227,ILE228,PRO230,VAL232 |
| EV-D68 | ILE120,GLU121,PRO122,VAL123,CYS124,VAL194,ARG240,ARG241,PHE242,LYS243,GLU314,TYR315, ARG318 |
| PV-2 | ASN13,GLY17,LEU18,VAL21,SER22,LEU104,THR107,ILE108,ASN109,GLU150,THR152,LYS168,GLN169,GLN170,GLY171THR214,ASN216 |
| PV-3 | ILE25,ILE36,ILE37,PRO38,SER84,SER87,LYS88,PHE90,ARG144,ALA147,GLU148,ASN151,THR152,SER153,THR154 |
| CV-A16 | LYS24,ILE29,TRP31,GLN52,LEU55,LEU56,GLN59,SER66,SER69,GLN70,LEU73,GLU74 |
| CV-B3 | TYR111,ILE112,PHE114,SER116,CYS118,ARG119,ILE120,GLU121,PRO122,LEU212,PHE213,THR214,SER215,PRO216,TYR314,HIS318 |
| HRV-B | ILE26,LEU29,ILE30,ILE33,ILE56,GLN60,GLN119,ARG120,THR121,GLU122,SER197,ASN317,LYS318,ILE321,THR322,LEU325 |
| Echovirus E11 | TYR111,PHE114,LYS115,SER116,CYS118,ARG119,ILE120,THR214,SER215,PRO216,TYR314,HIS318,GLU325 |

19）R523062

| Enteroviruses | Amino acids |
| --- | --- |
| EV-A71 | LEU5,LYS7,PHE8,ASN9,PHE28,ILE29,ASP30,ASN79,LEU83,HIS85,PHE86,ARG144,ASP148,LYS149,LYS279 |
| EV-D68 | LEU15,ARG16,GLY17,LEU18,TRP20,LEU21,GLN72,ALA75,LEU76,ASN79,TYR83,MET268,TYR279,ARG281,ILE286 |
| PV-1 | LYS49,GLN52,GLU57,GLN59,ILE60,MET187,LYS188,CYS191,GLN192,SER195,VAL197,PHE199,ALA237,LEU238,ARG241 |
| PV-3 | LYS24,ILE25,ILE29,SER84,GLN86,SER87,LYS88,PHE90,ARG144,ALA147,GLU148,ASN151,THR152,SER153,GLN170 |
| CV-A6 | TYR82,HIS85,PHE86,LYS98,ILE141,ARG144,ALA145,ASP148,LEU269,ASN277,PHE278,LYS279,ARG280,CYS281,SER282 |
| CV-A10 | ASN109,MET112,GLN113,LYS115,ARG119,ILE120,SER152,GLN169,VAL171,THR214,LYS216 |
| CV-A21 | HIS64,ASN79,TRP82,LEU83,GLN86,PHE90,TYR94,PRO229,SER235,ASP236,ASN239,ARG240 |
| CV-A24 | GLN113,PHE114,LYS115,LYS117,HIS118,ARG119,ILE120,THR214,ASP216,LYS314,ALA318,GLY321,GLU325 |
| CV-B3 | ALA14,CYS15,MET18,LYS24,LYS27,PHE28,TRP31,LEU32,PHE83,CYS97,TYR90,ALA91,TYR94,SER156,LEU157,PRO158,PRO159 |
| HRV-A2 | LYS112,ARG114,CYS115,GLU116,PRO117,VAL118,ASP207,SER208,ARG209,ASP307,ARG311 |
| HRV-B | ILE26,LEU29,ILE30,ILE56,GLN60,ILE109.GLN119,ARG120,THR121,GLU122,SER197,LYS318,ILE321,THR322,LEU325,GLU326 |
| HRV-C | GLN71,LEU74,MET75,LEU78,THR138,ILE150,SER152,LEU153,PRO154,PRO155,MET170,MET171,ASP172,ASP173 |
| Echovirus E30 | ILE21,LYS24,ILE25,PHE83,TYR86,CYS87,MET108,GLU148,ASN151,SER152,SER153,LYS168,GLN169,GLN170 |

20）Dibucaine derivatives 6i

| Enteroviruses | Amino acids |
| --- | --- |
| EV-A71 | PRO159,ASP160,PRO161,ASP177,CYS179,GLN180,ASN181,PRO182,ASP183,GLY184,LYS185,ASP186,MET187, |
| PV-1 | PHE46,LYS49,GLU57,GLN59,ILE60,GLU121,CYS188,CYS191,GLN192,SER195,THR196,VAL197,GLU198,PHE199,ILE200,VAL232,LEU238,ARG240,ARG241 |
| PV-2 | ILE25,ILE29,LEU32,ASP42,LYS43,LYS49,TRP82,LEU83,ILE85,LYS88,ARG144,ALA147,GLU148,ASN151,THR152,SER153,THR154,LYS279 |
| CV-A6 | TYR82,HIS85,PHE86,ARG88,LYS89,PRO92,GLY140,ILE141,ARG144,ALA145,ASP148,ASN277,PHE278,LYS279 |
| CV-A10 | PRO54,LEU55,MET56,GLU57,ASN58,GLN59,ASN62,SER66,ALA67,LEU73,TYR82,LEU83,ALA84,HIS85,PHE86,ARG88,LYS89 |
| CV-A21 | GLU57,ILE60,ALA61,HIS64,ASN79,TRP82,LEU83,GLN86,PHE90,TYR94,SER235,ASP236,ASN239,ARG240 |
| CV-B3 | LYS7,GLU10,MET11,ASN13,ALA14,CYS15,MET18,PHE83,CYS87,PRO158,PRO159,ASP160,PRO161,ASP162,HIS163,ASP165,GLN180 |
| HRV-A | TRP20,LYS24,LEU62,LYS86,PHE87,LEU90,TYR91,GLU94,ASN232,ARG233,PHE235,PHE236,LEU237,ASN289,THR290,TYR294,ASN302,GLU306,ARG310 |
| HRV-B | ILE22,ILE26,THR63,ILE66,TYR112,PHE115,SER117,LYS118,GLN119,SER197,VAL198,ASP199,GLU326,PHE329,GLN330 |
| HRV-C | PHE28,PHE70,GLN71,LEU74,MET75,LEU78,SER134,ILE150,TYR151,SER152,LEU153,PRO154,PRO155 |
| Echovirus E30 | TRP20,ILE21,LYS24,ILE25,PHE28,PHE83,TYR86,CYS87,LYS89,TYR90,MET108,ASN151,SER152,SER153,LYS168,GLN170 |

21）TBZE-029

| Enteroviruses | Amino acids |
| --- | --- |
| PV-1 | THR48,LYS49,LYS51,GLN52,GLN59,LYS188,CYS191,GLN192,SER195,VAL197,VAL232,ALA233,HIS234,ALA237,LEU238,ARG241 |
| PV-3 | TRP4,PHE8,LYS24,ILE25,PHE28,ILE29,ILE36,ILE37,PRO38,LYS88,PHE90,GLU148,ASN151,THR152,SER153,GLN170 |
| CV-A6 | MET112,LYS115,HIS118,ARG119,ILE120,PRO122,PHE213,THR214,SER215,LYS216,TYR314,SER318,GLY321,ASN322,GLU325 |
| CV-A10 | VAL101,LEU104,GLU105,MET108,MET112,ASP148,LYS149,TYR150,HIS151,VAL171,LYS216,TYR314 |
| CV-A16 | SER22,SER23,LYS24,ILE29,TRP31,LEU55,SER69,GLN70,LEU73,MET76 |
| CV-A21 | NONE |
| CV-B3 | TYR111,ILE112,PHE114,SER116,CYS118,ARG119,ILE120,ASP198,LEU212,PHE213,THR214,TYR314,HIS318 |
| HRV-B | ILE102,LEU105,LYS106,TYR150,PHE151,GLU172,TYR315,LYS316,MET319 |
| HRV-C | ARG117,THR118,GLU119,PRO120,VAL121,ASN212,SER311,ARG314,ARG315,ILE317,HIS318,THR319 |
| Echovirus E11 | MET108,TYR111,PHE114,LYS115,SER116,CYS118,ARG119,ILE120,GLU121,GLN169,THR214,PRO216,TYR314,HIS318,GLU325 |
| Echovirus E30 | MET18,TRP20,ILE21,LYS24,ILE25,TYR82,PHE83,TYR86,CYS87,VAL101,LEU104,GLU105,MET108,ASN151,SER152,SER153 |

22）Zuclopenthixol

| Enteroviruses | Amino acids |
| --- | --- |
| PV-1 | CYS12,ASN13,TRP20,VAL21,PHE28,ARG41,LYS49,CYS191,SER195,VAL232,ALA233,HIS234,ALA237,LEU238,ARG240,ARG241 |
| PV-3 | TRP4,PHE8,ILE25,PHE28,ILE36,PRO38,LEU83,ARG144,ALA147,GLU148,ASN151,THR152,SER153,THR154 |
| CV-A10 | LYS7,VAL101,LEU104,GLU105,MET108,ASN109,MET112,TYR150,HIS151,SER152,VAL171,LYS216,TYR314,SER315,SER318 |
| CV-B3 | TYR111,ILE112,PHE114,LYS115,SER116,LYS117,CYS118,ARG119,ILE120,ASP198,PHE199,LEU212,THR214,HIS318,GLY321,THR322,GLU325 |
| HRV-C | ARG117,THR118,GLU119,THR192,THR193,THR210,ASN212,ARG314,ARG315,ILE317,HIS318,THR319,ASN322 |
