# Supplementary material for: Structure Prediction and Potential Inhibitors Docking of Enterovirus 2C Proteins
Source: Front Microbiol. 2022 Apr 29;13:856574. doi: 10.3389/fmicb.2022.856574 (PMC9100428; doi:10.3389/fmicb.2022.856574)

Supporting information

Figure S1. Overview of the 2C protein structures from enteroviruses by trRosetta server. (A) EV-A71 (B) EV-D70 (C) EV-D68 (D) PV-1 (E) PV-2 (F) PV-3 (G) CV-A6 (H) CV-A9 (I) CV-A10 (J) CV-A16 (K) CV-A21 (L) CV-A24 (M) CV-B3 (N) RV-A (O) HRV-A2 (P) RV-B (Q) HRV-B14 (I) RV-C (S) Echovirus E11 (T) Echovirus E30.

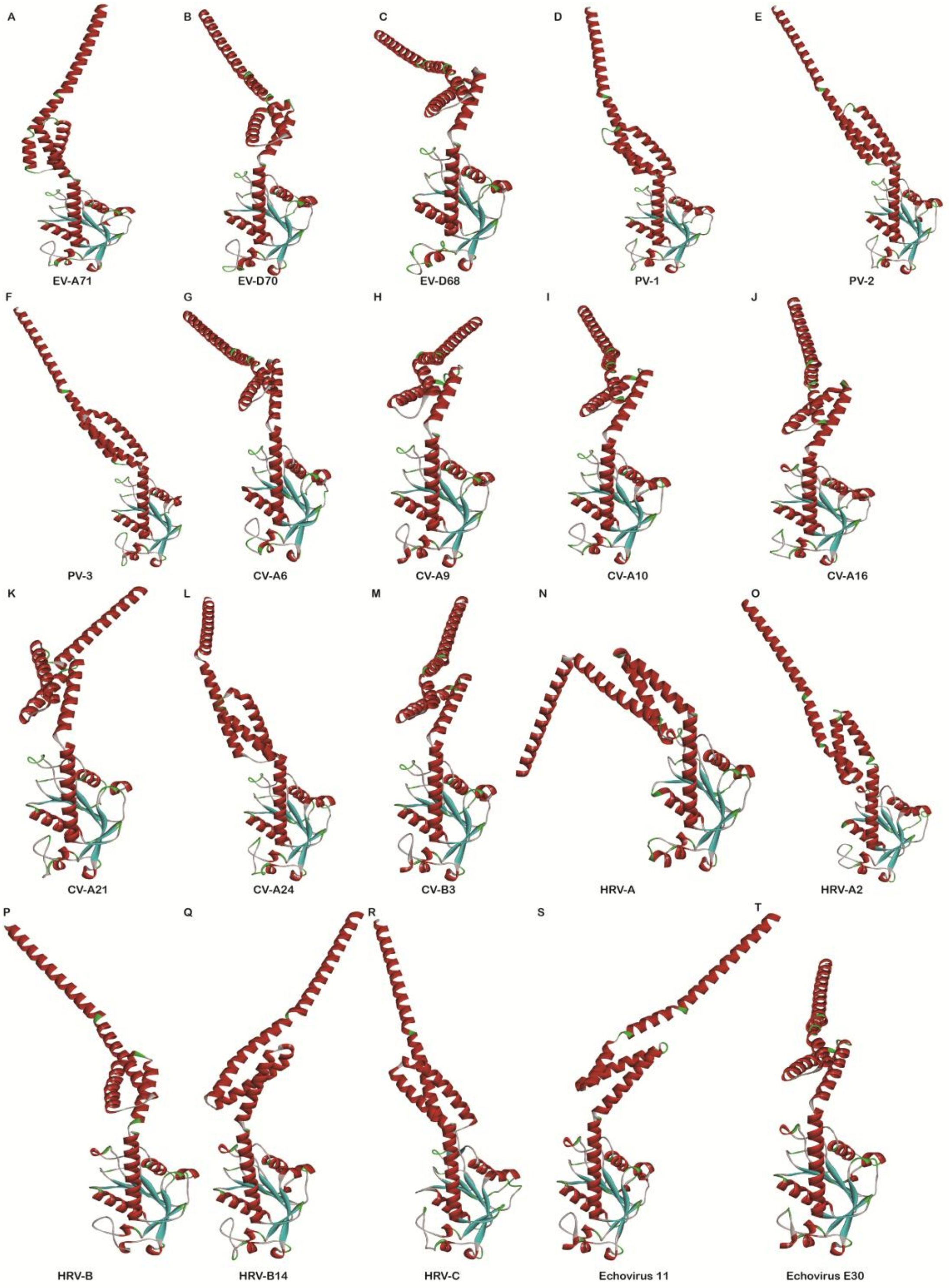

Figure S2. The poses with minimum free energy of the compound Thiophene-2-carboxylic acid benzyl-pyridin-2-yl-amide(R523062) along with its corresponding interactions plots within the active sites of 2C proteins.

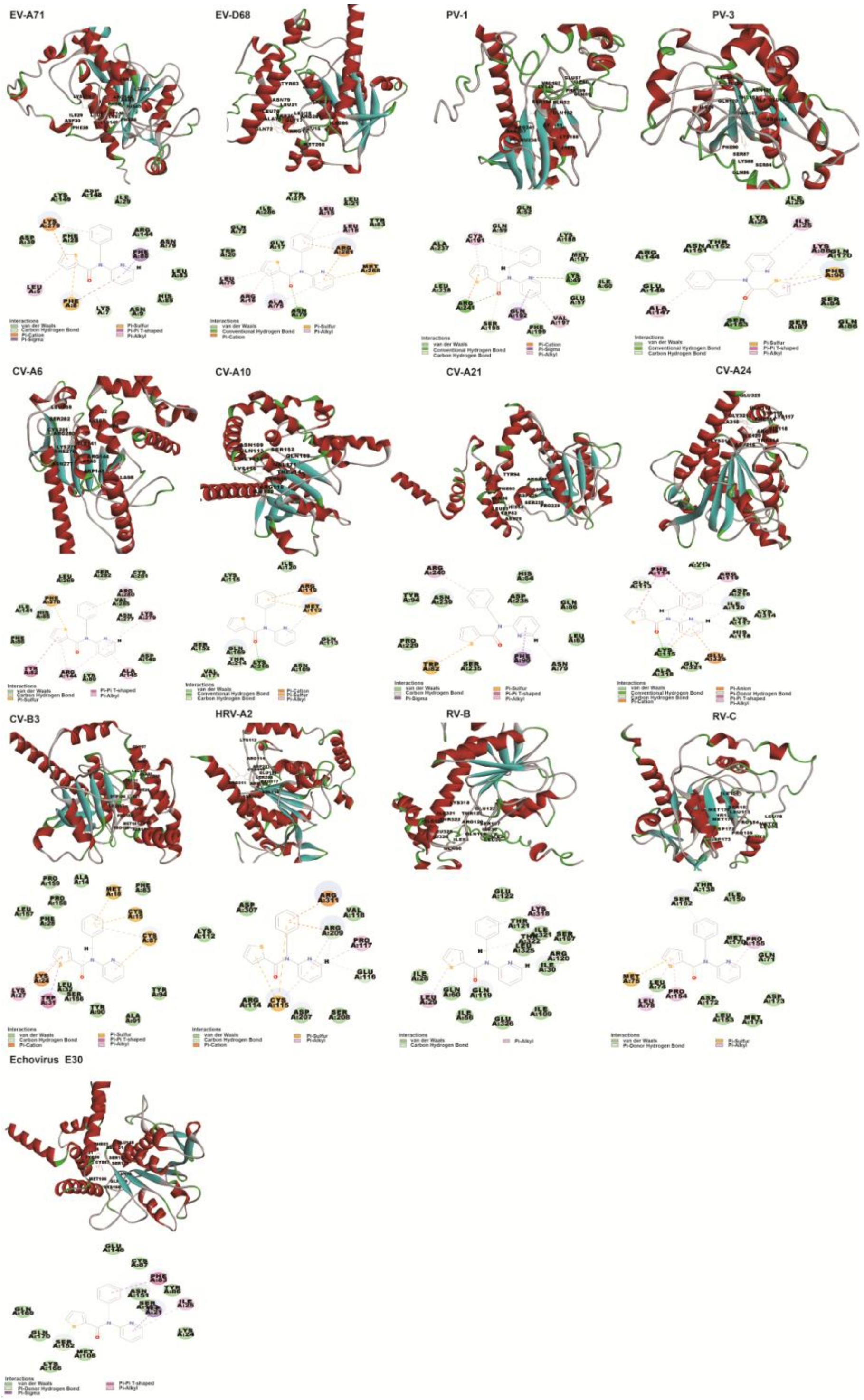

Figure S3. The poses with minimum free energy of the compound N-(4-Fluorobenzyl)-N-phenylfuran-2-carboxamide (compound 19b) along with its corresponding interactions plots within the active sites of 2C proteins.

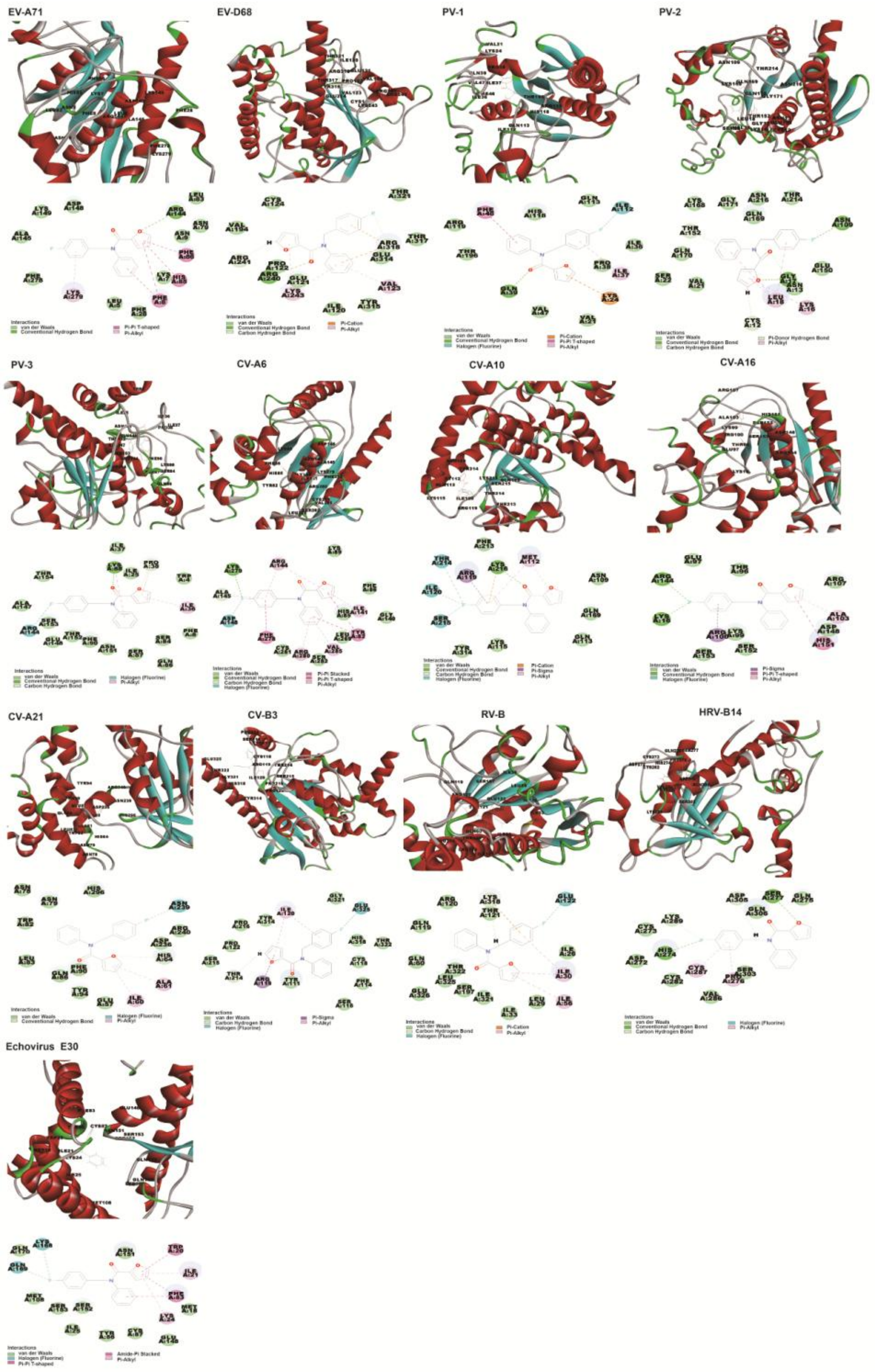

Figure S4. The poses with minimum free energy of the compound N-(4-Fluorobenzyl)-N-phenyl-1H-pyrrole-3-carboxamide (compound 12b) along with its corresponding interactions plots within the active sites of 2C proteins.

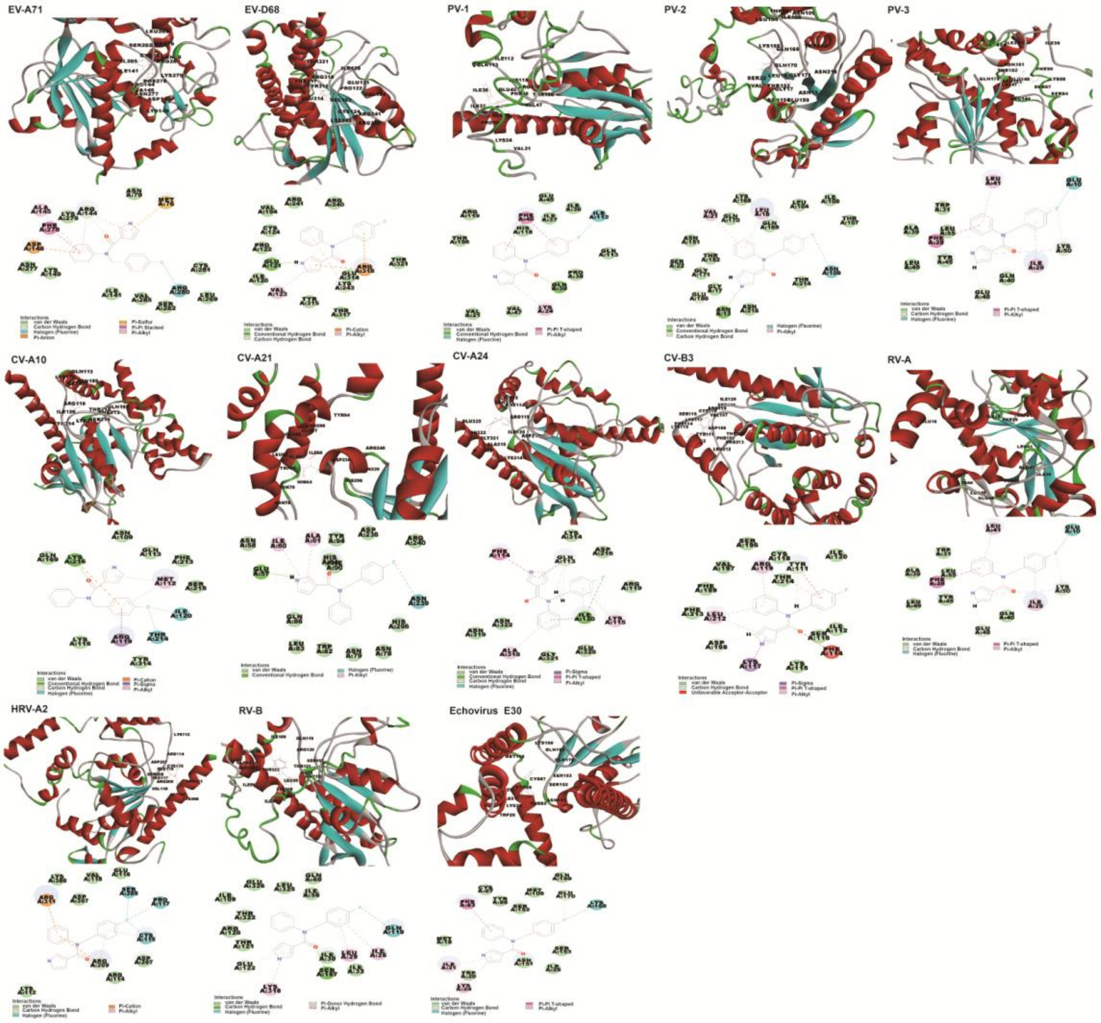

Figure S5. The poses with minimum free energy of the compound N-(4-Fluorobenzyl)-N-(4-methoxyphenyl)-1H-pyrrole-3-carboxamide (compound 12a) along with its corresponding interactions plots within the active sites of 2C proteins.

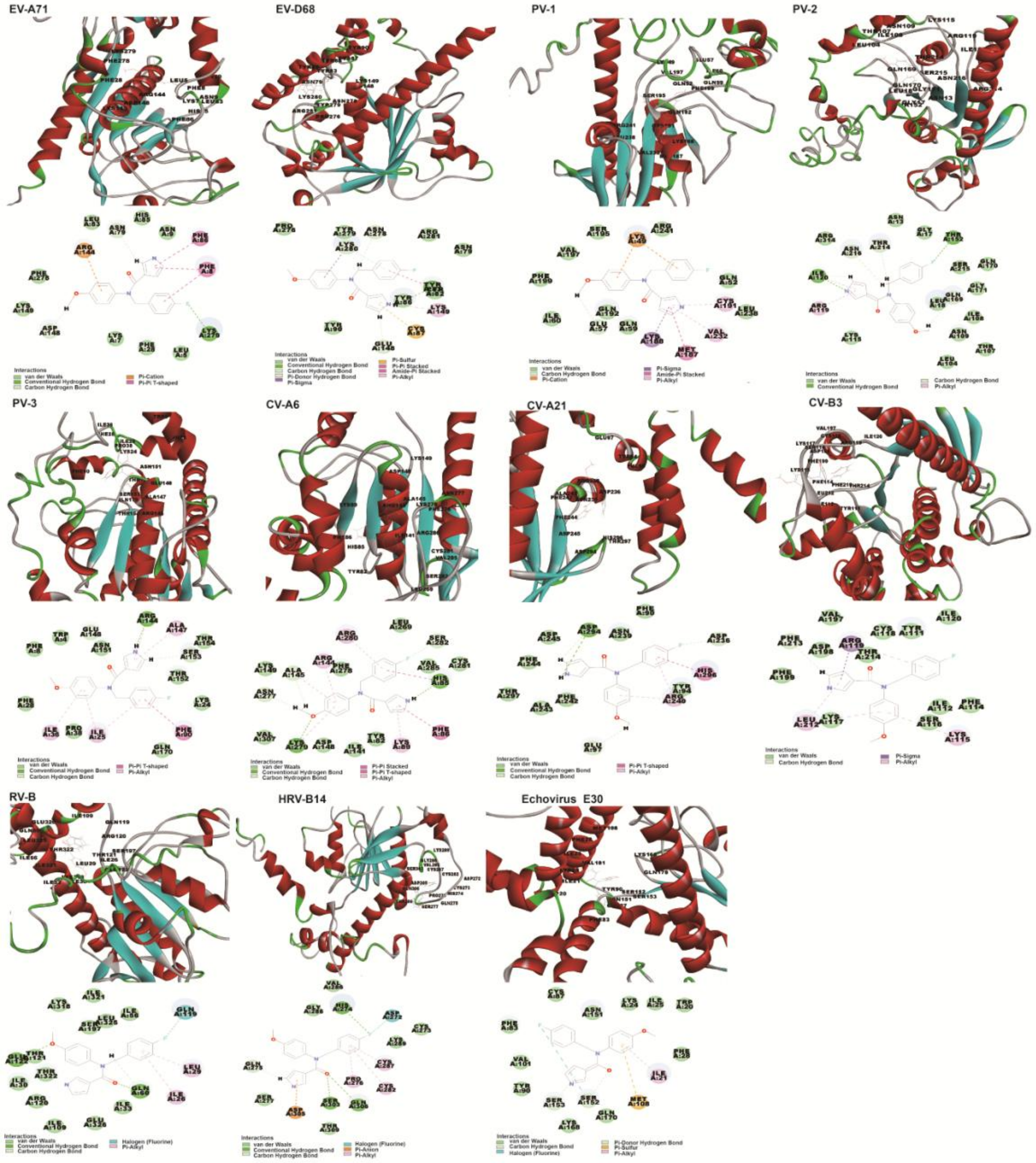

Figure S6. The poses with minimum free energy of the compound N-[2-(dimethylamino)ethyl]-2-(thiophen-2-yl) quinolone-4-carboxamide (quinoline analogs 12a) along with its corresponding interactions plots within the active sites of 2C proteins.

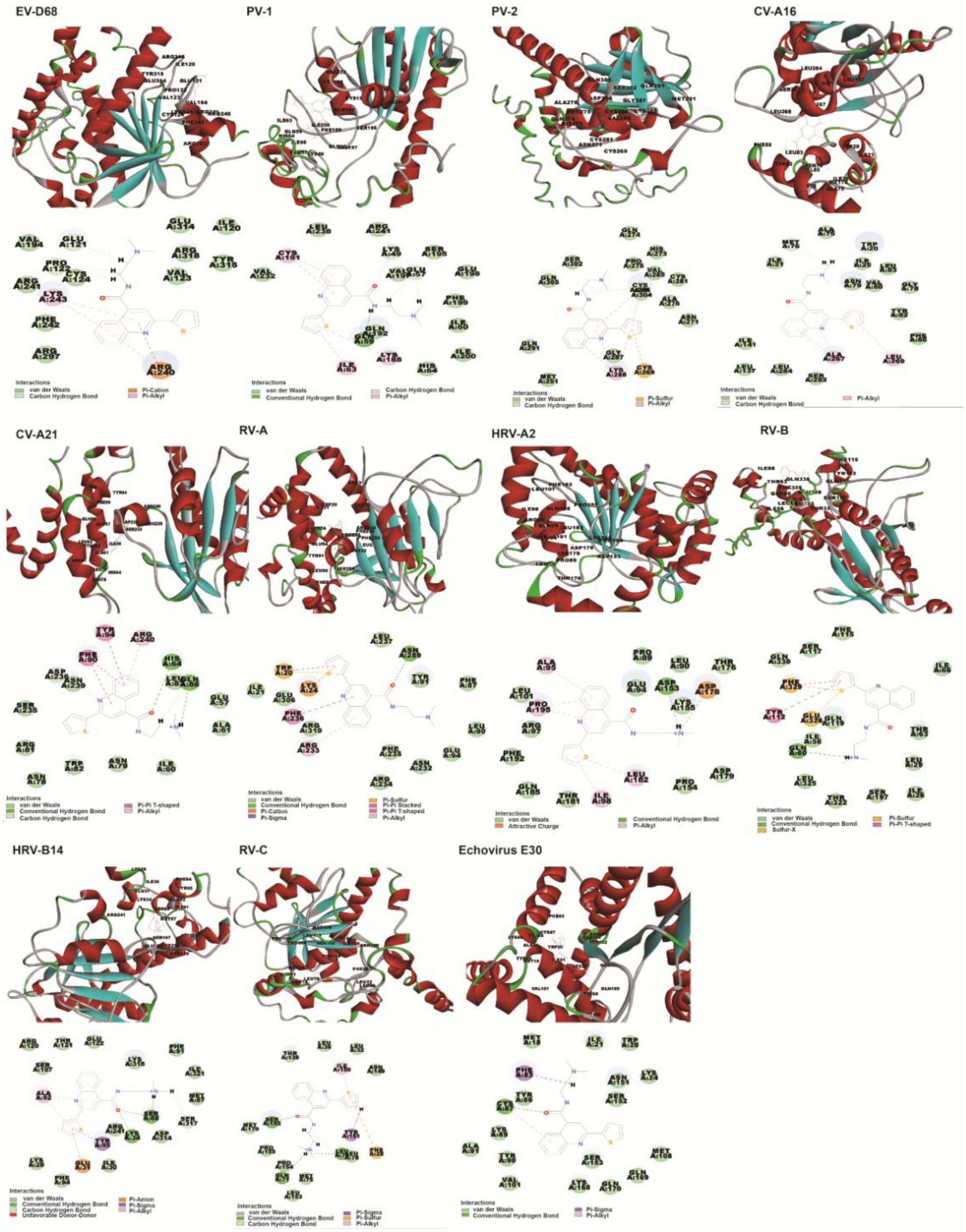

Figure S7. The poses with minimum free energy of the compound N6-benzyladenosine along with its corresponding interactions plots within the active sites of 2C proteins.

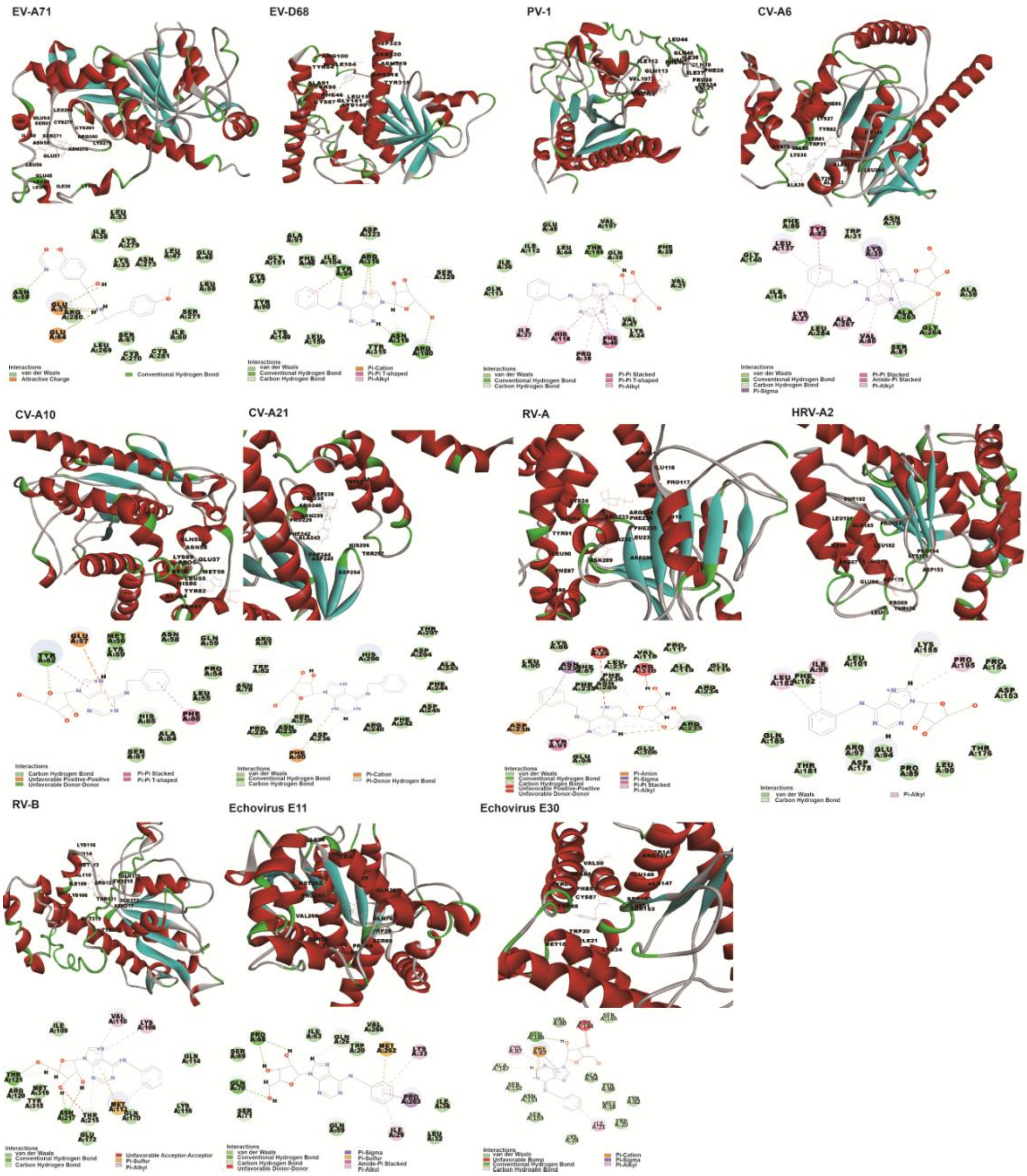

Figure S8. The poses with minimum free energy of the compound 2-Butoxy-N-(2-(dimethylamino) ethyl)quinoline-4-carboxamide (dibucaine derivatives 6i) along with its corresponding interactions plots within the active sites of 2C proteins.

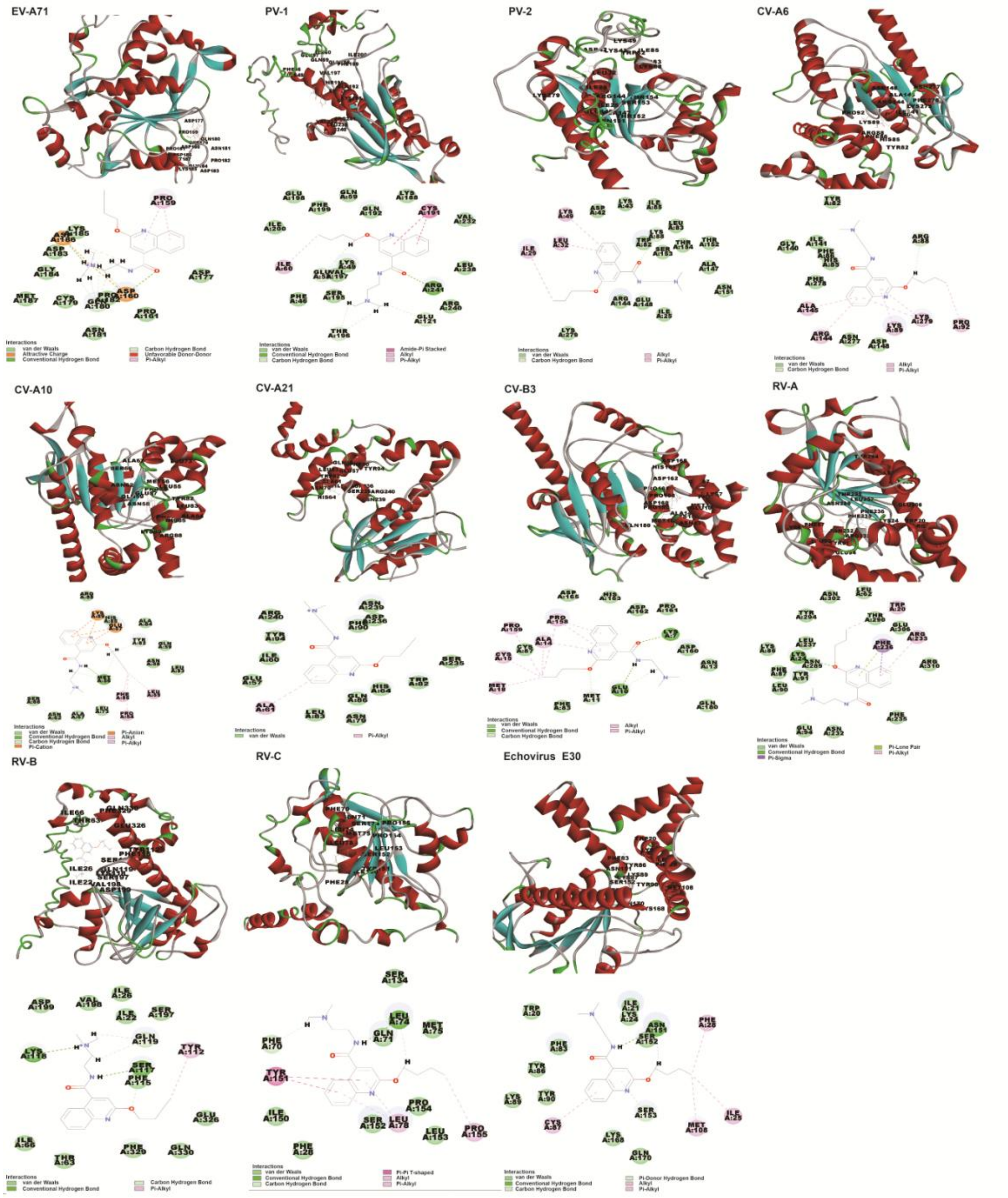

Figure S9. The poses with minimum free energy of the compound TBZE-029 along with its corresponding interactions plots within the active sites of 2C proteins.

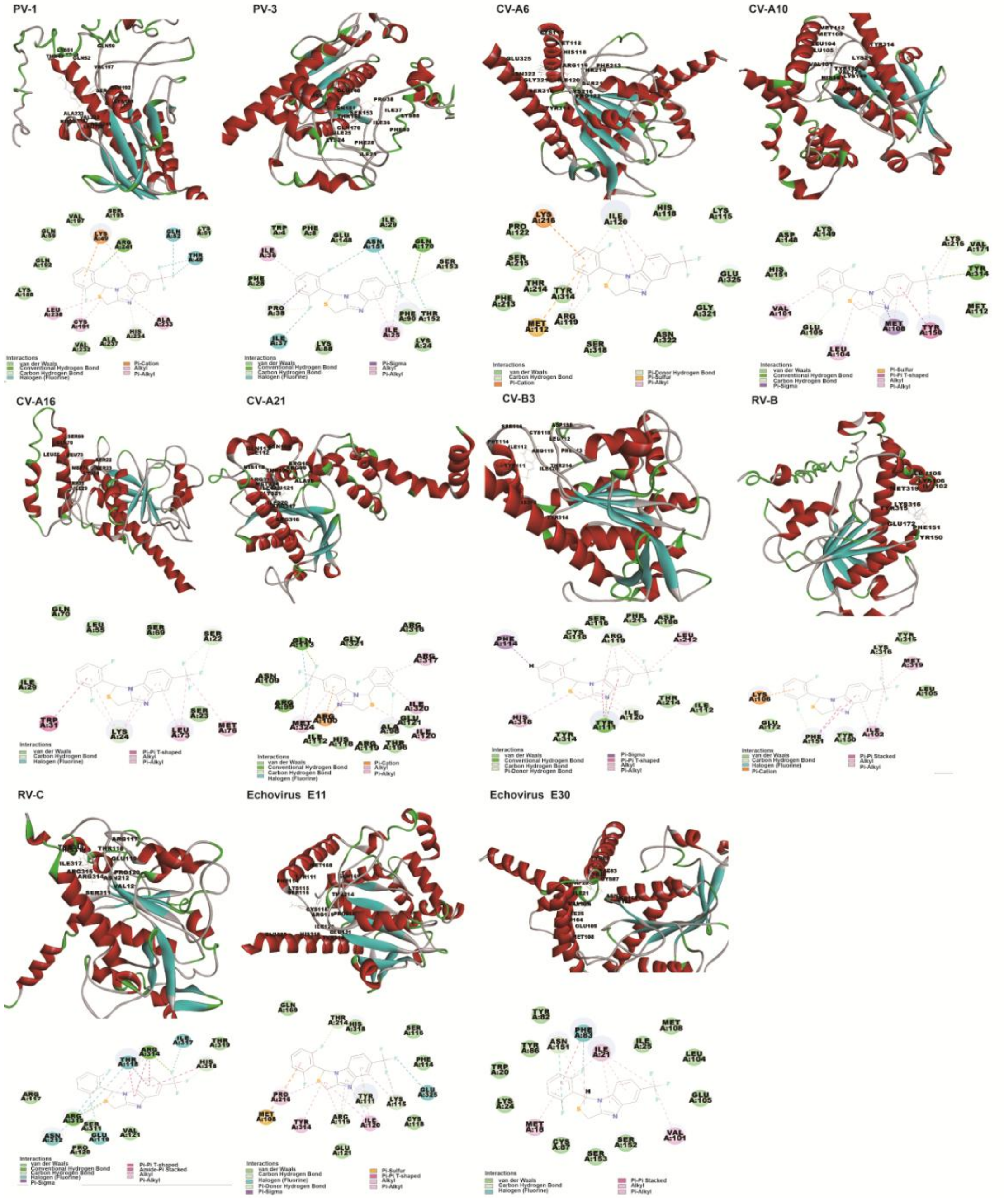

Figure S10. The poses with minimum free energy of the compound 1-(3-phenyl-3-(4-(trifluoromethyl) phenoxy)propyl)guanidine (fluoxetine analogue 2b) along with its corresponding interactions plots within the active sites of 2C proteins.

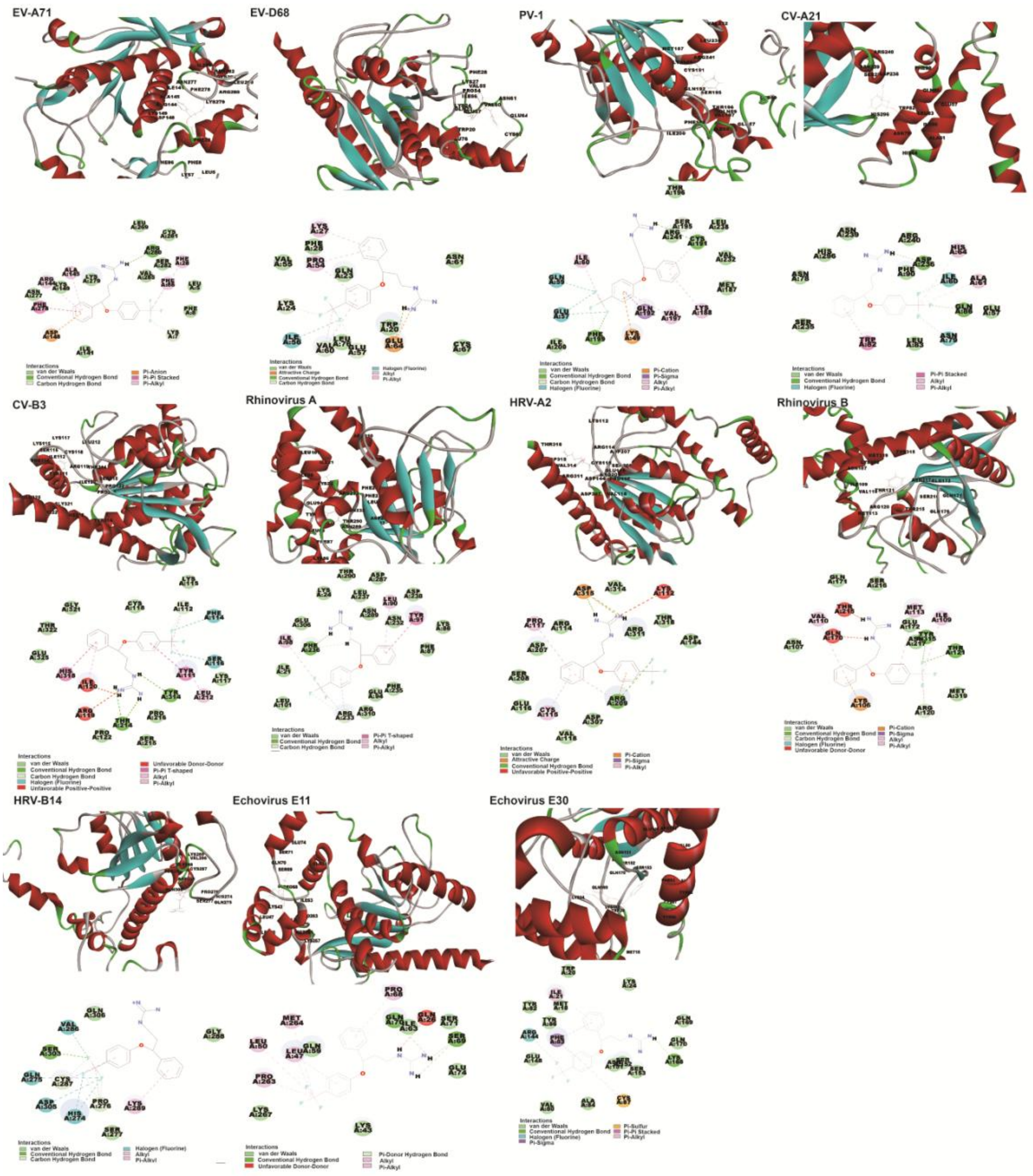

Figure S11. The poses with minimum free energy of the compound N-phenyl-N-(4-(trifluoromethyl)benzyl)furan-2-carboxamide (compound 19d) along with its corresponding interactions plots within the active sites of 2C proteins.

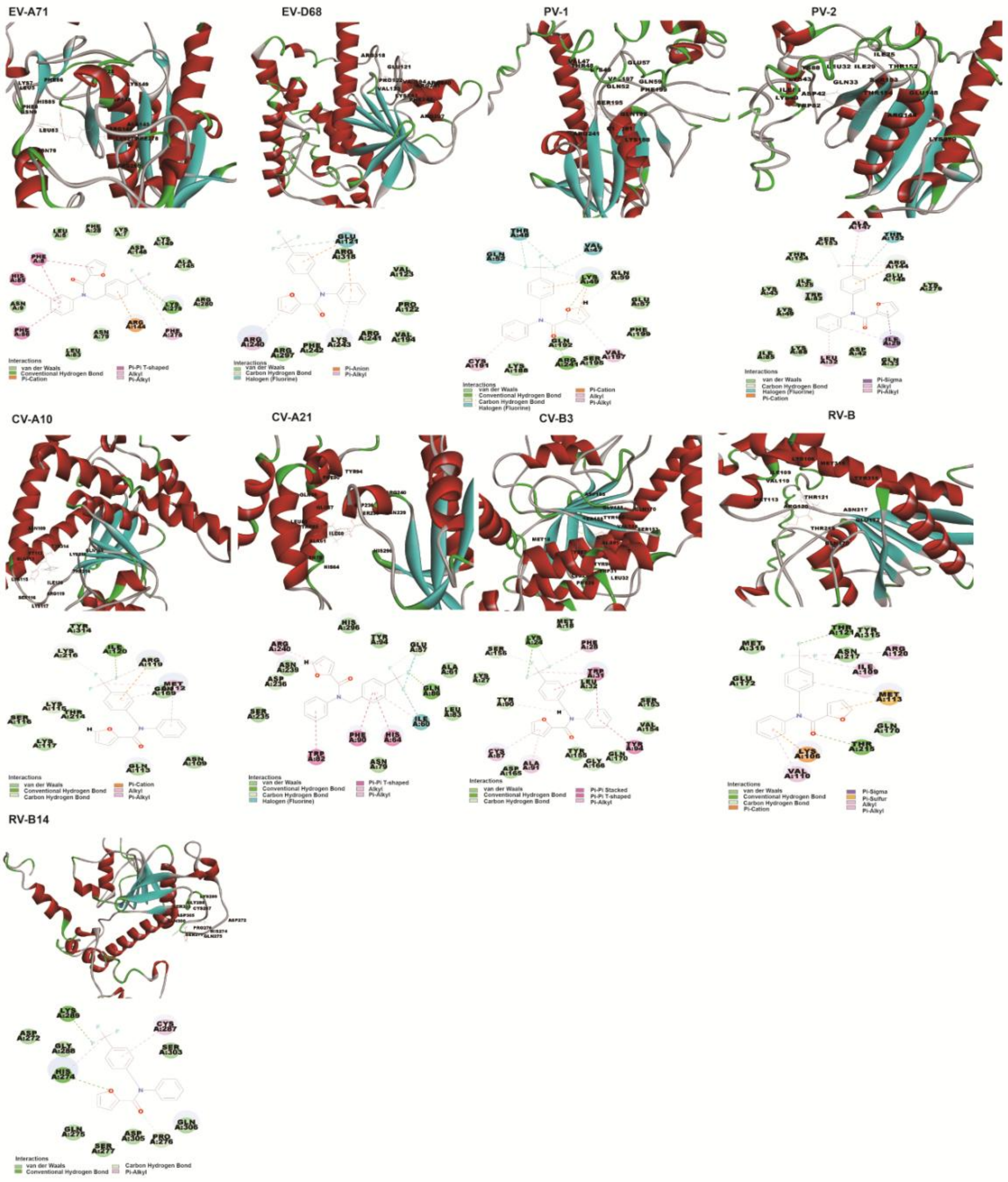

Figure S12. The poses with minimum free energy of the compound dibucaine along with its corresponding interactions plots within the active sites of 2C proteins.

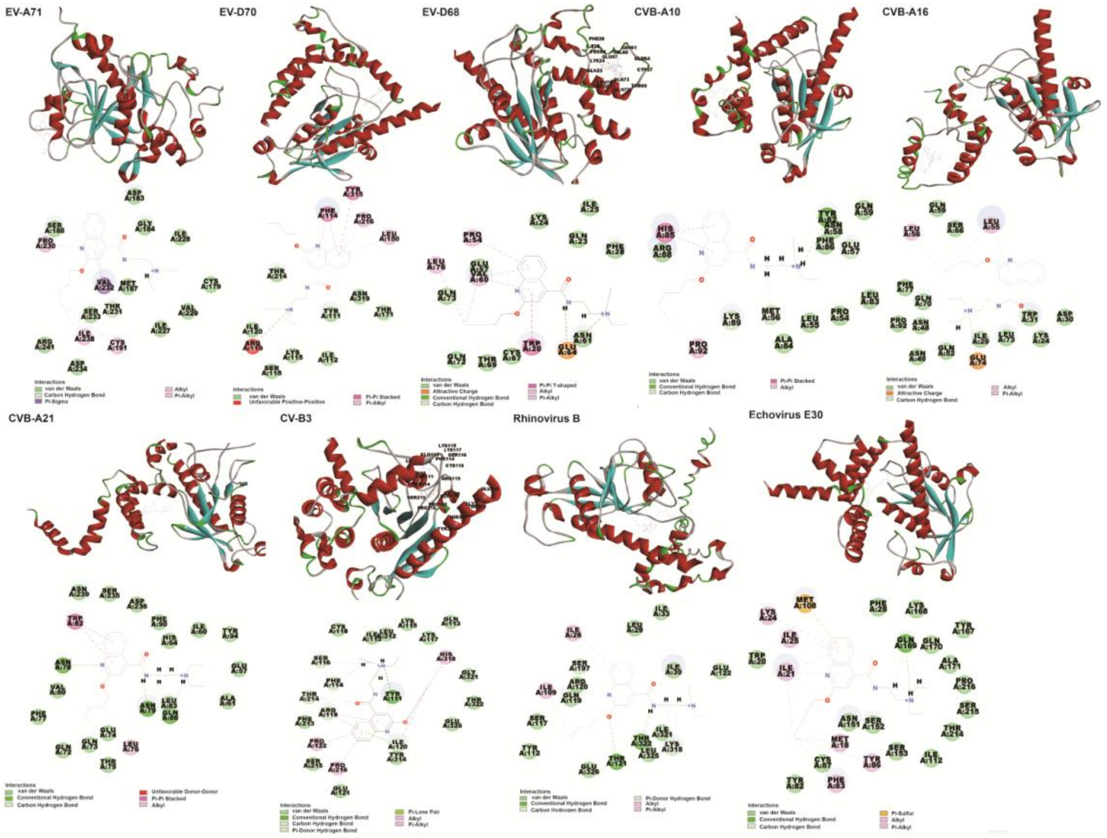

Figure S13. The poses with minimum free energy of the compound HBB along with its corresponding interactions plots within the active sites of 2C proteins.

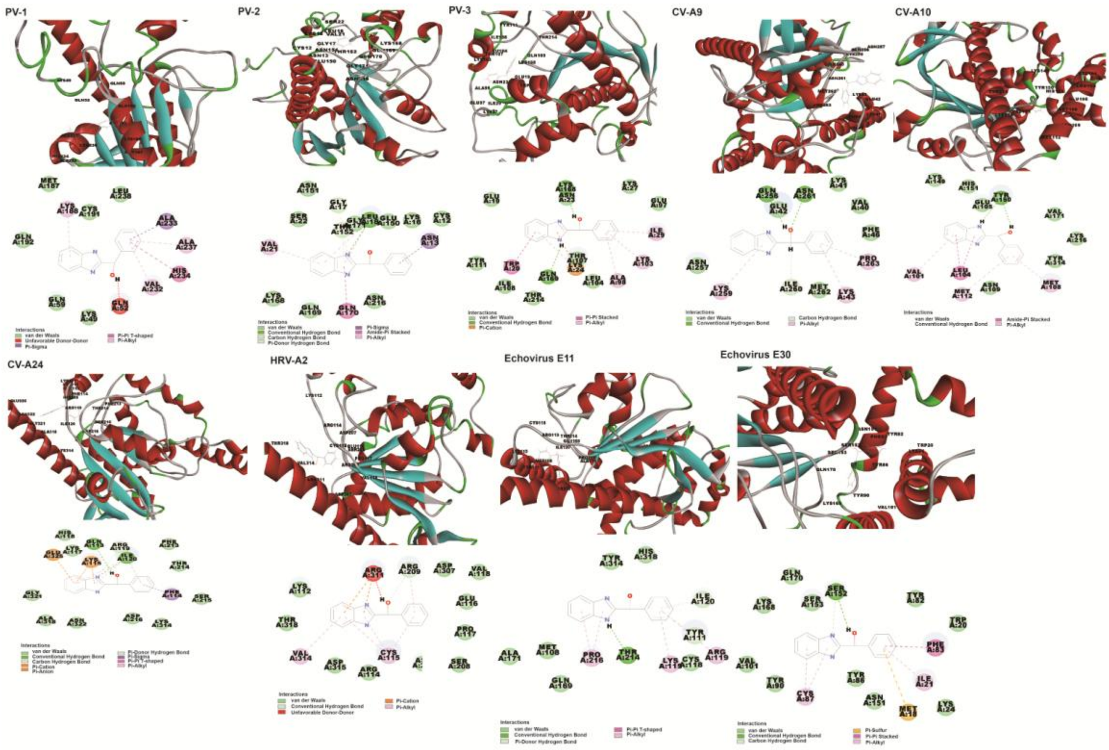

Figure S14. The poses with minimum free energy of the compound metrifudil along with its corresponding interactions plots within the active sites of 2C proteins.

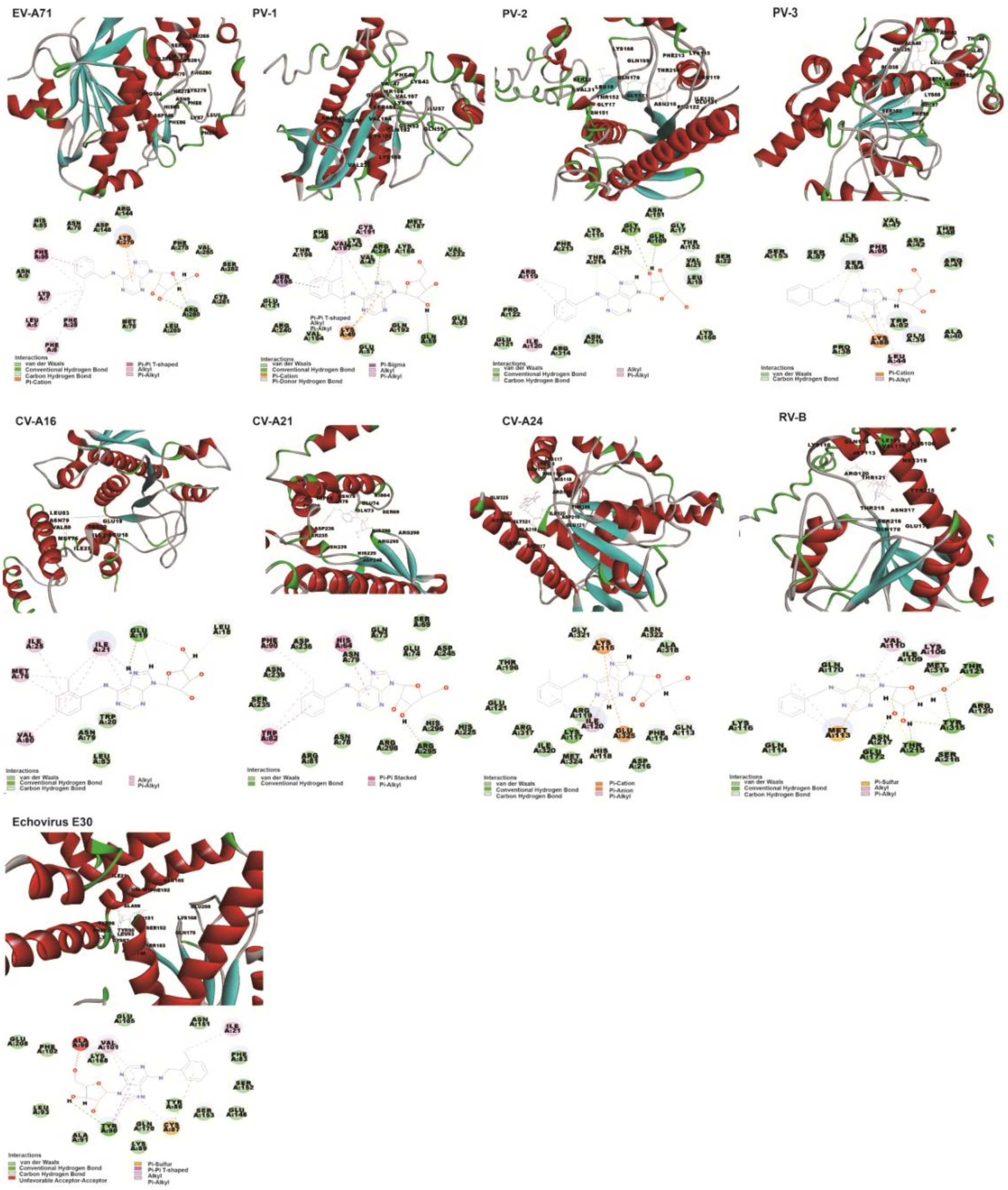

Figure S15. The poses with minimum free energy of the compound pirlindole along with its corresponding interactions plots within the active sites of 2C proteins.

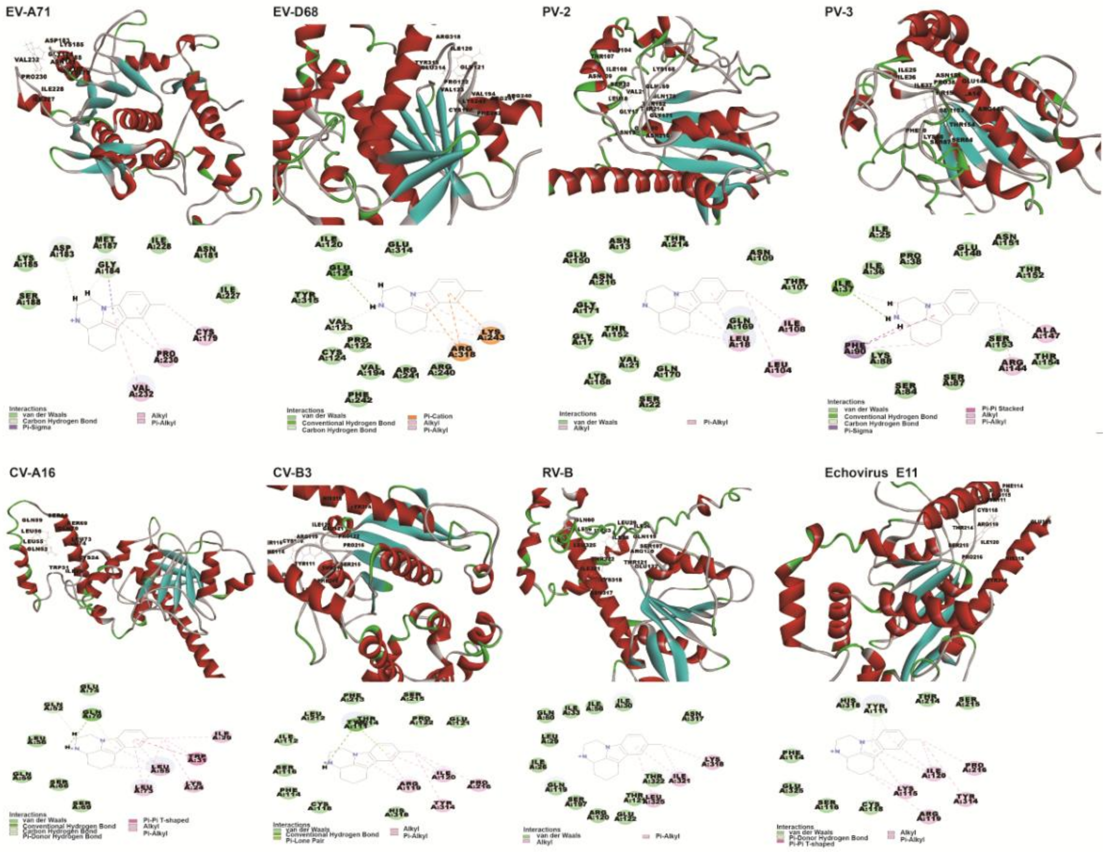

Figure S16. The poses with minimum free energy of the compound MRL-1237 along with its corresponding interactions plots within the active sites of 2C proteins.

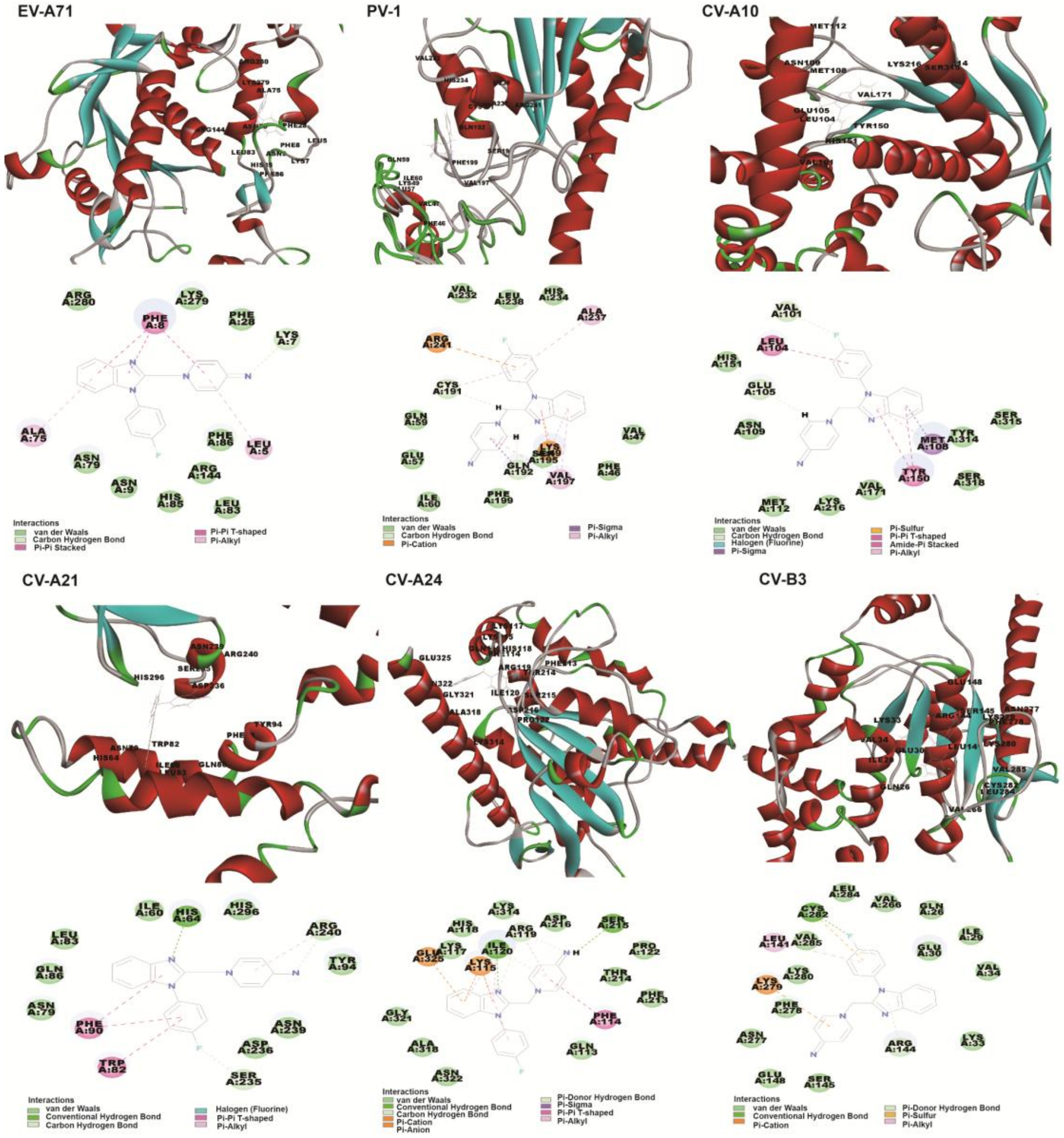

Figure S17. The poses with minimum free energy of the compound N-(2-(dimethylamino) ethyl)-2-phenylquinoline-4-carboxamide (quinoline analogs 10a) along with its corresponding interactions plots within the active sites of 2C proteins.

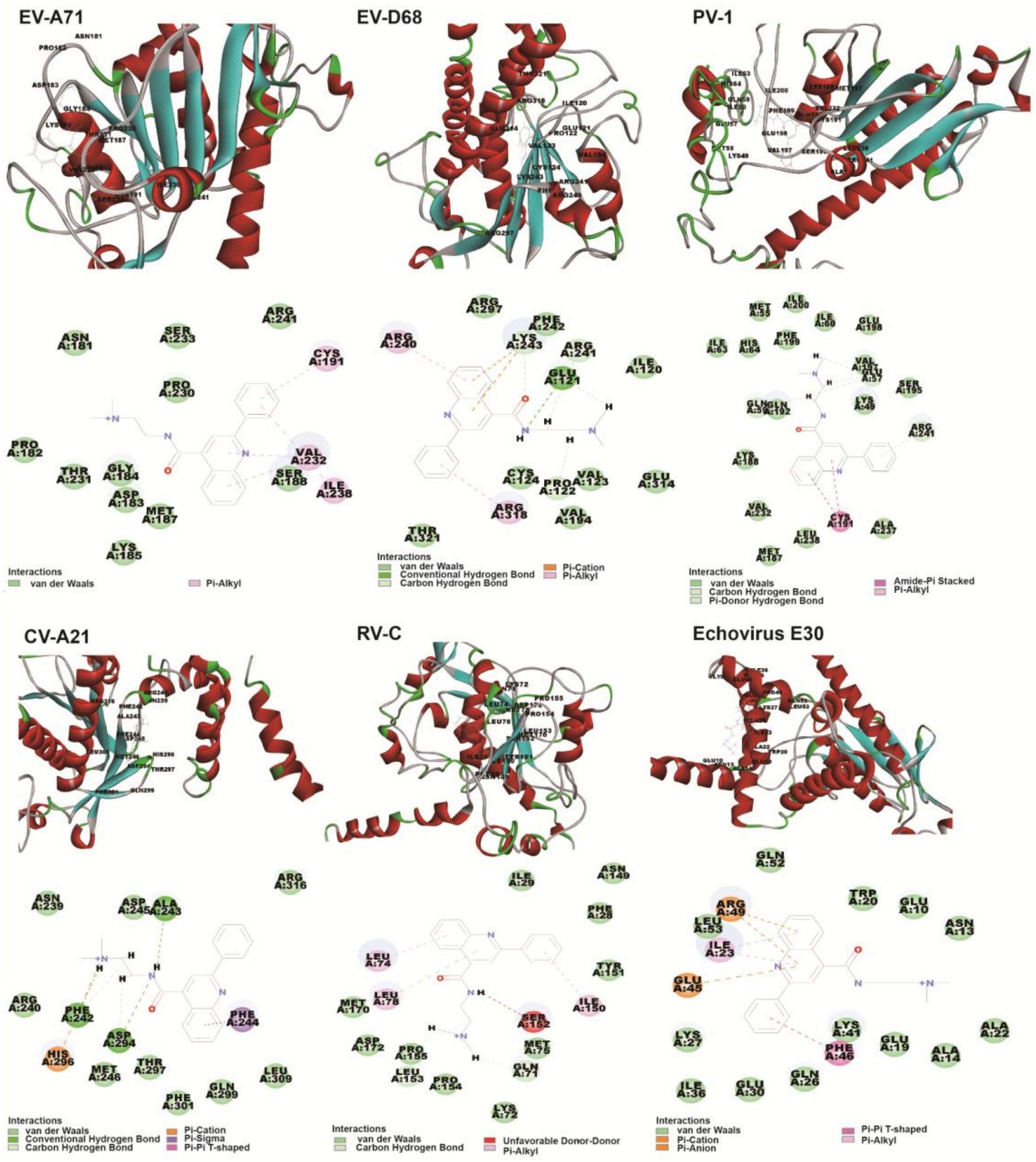

Figure S18. The poses with minimum free energy of the compound zuclopenthixol along with its corresponding interactions plots within the active sites of 2C proteins.

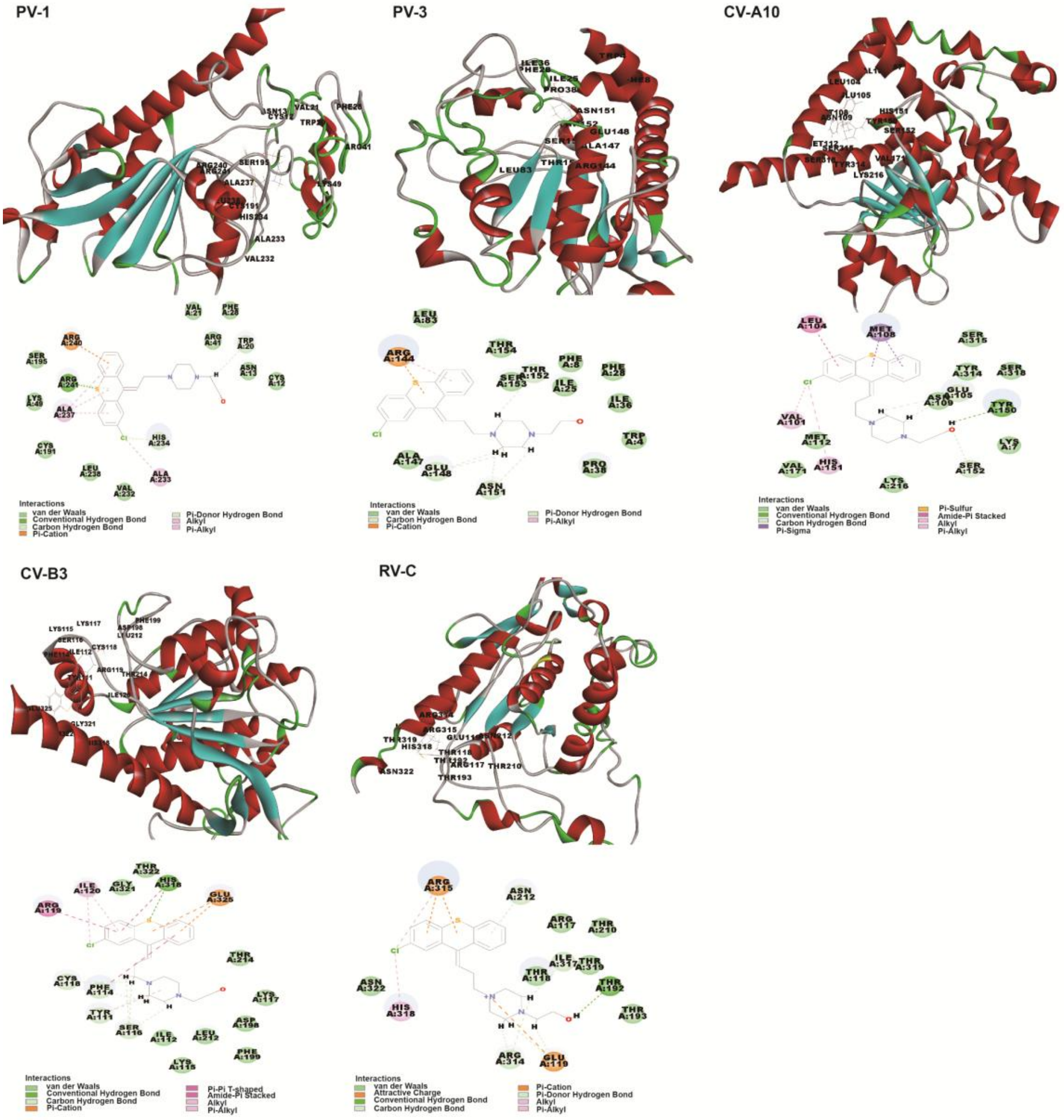

Figure S19. The poses with minimum free energy of the compound fluoxetine along with its corresponding interactions plots within the active sites of 2C proteins.

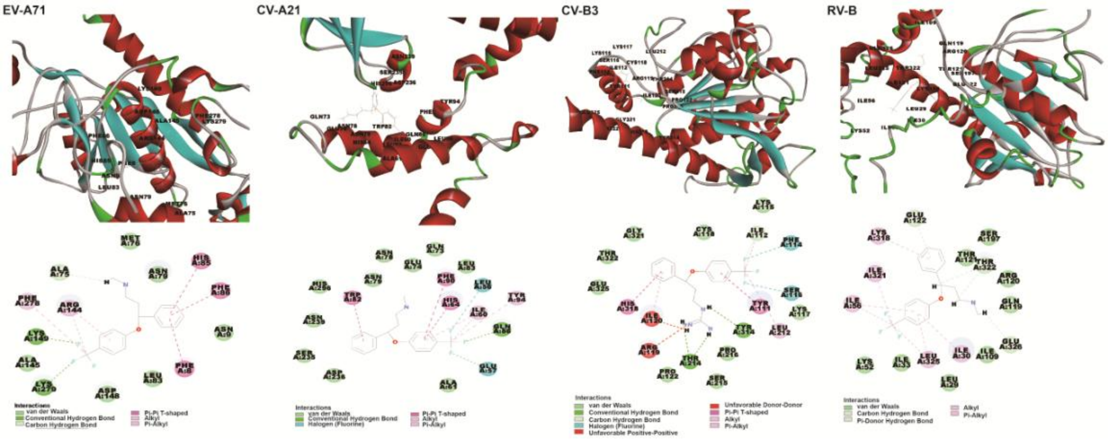

Figure S20. The poses with minimum free energy of the compound fluoxetine HCl along with its corresponding interactions plots within the active sites of 2C proteins.

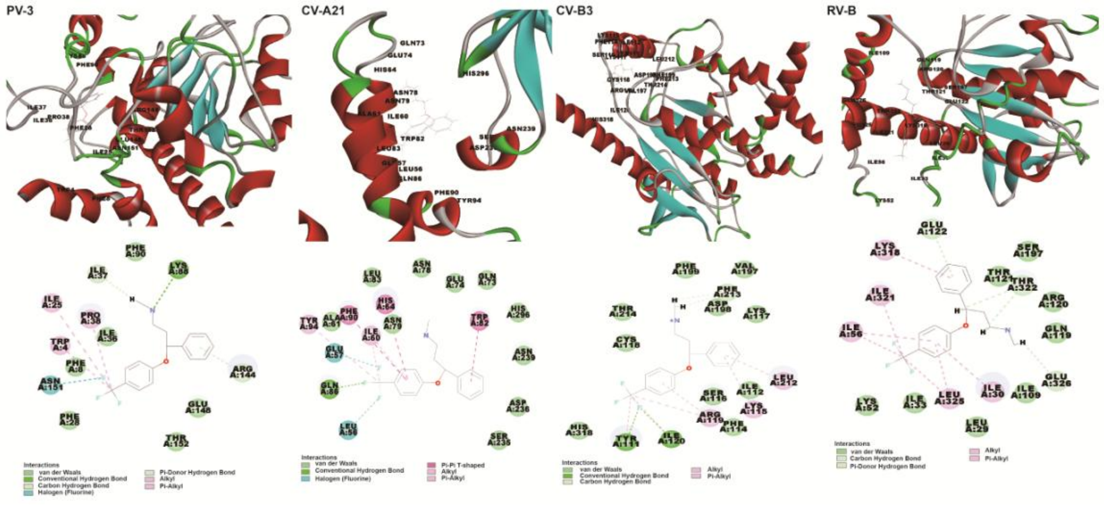

Figure S21. The poses with minimum free energy of the compound N-[2-(pyrrolidin-1-yl)ethyl]-2-(thiophen-2-yl) quinolone-4-carboxamide (quinoline analogs 12c) along with its corresponding interactions plots within the active sites of 2C proteins.

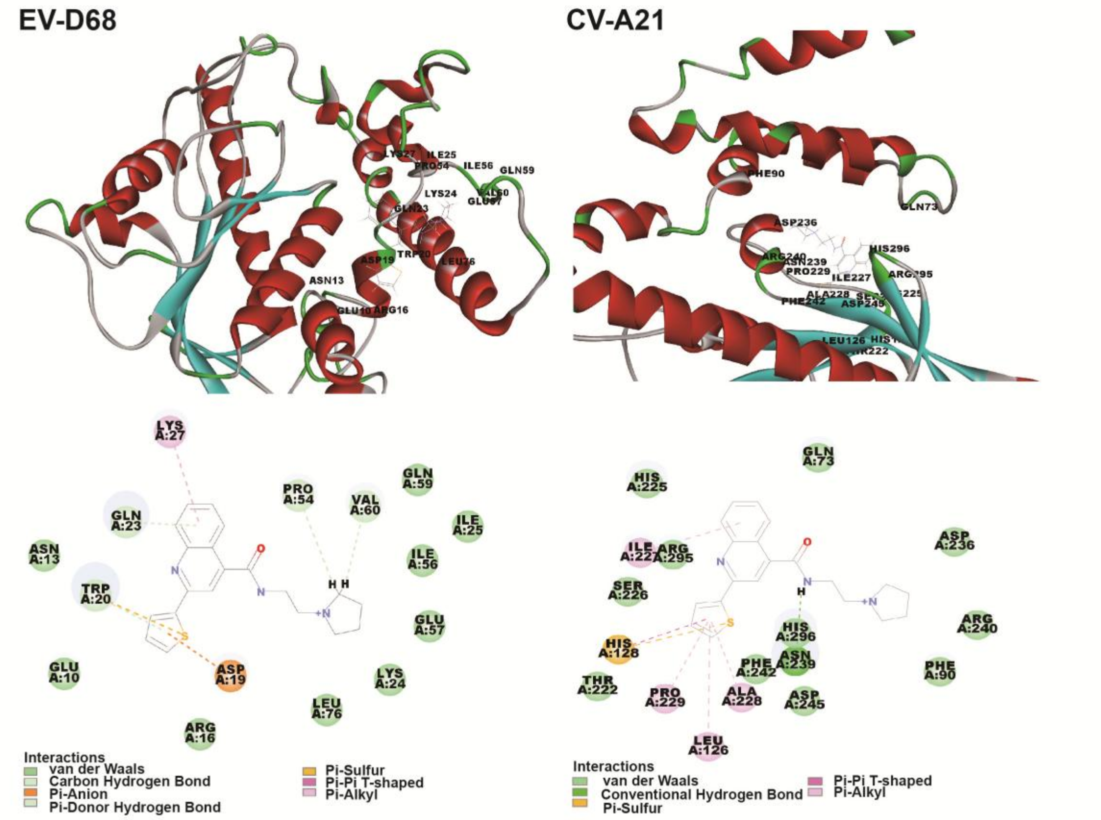

Supplement: Supplementary file 7 [file Data_Sheet_1.PDF]
